# Memantine before Mastectomy Prevents Post-Surgery Pain: A Randomized, Blinded Clinical Trial in Surgical Patients

**DOI:** 10.1371/journal.pone.0152741

**Published:** 2016-04-06

**Authors:** Véronique Morel, Dominique Joly, Christine Villatte, Claude Dubray, Xavier Durando, Laurence Daulhac, Catherine Coudert, Delphine Roux, Bruno Pereira, Gisèle Pickering

**Affiliations:** 1 CHU Clermont-Ferrand, Inserm CIC 1405, Centre de Pharmacologie Clinique, F-63003 Clermont-Ferrand, France; 2 Centre Jean Perrin, Centre de Lutte contre le Cancer, 58 rue Montalembert, F-63000 Clermont-Ferrand, France; 3 Clermont Université, Université d’Auvergne, Pharmacologie Fondamentale et Clinique de la Douleur, Laboratoire de Pharmacologie, Facultés de Médecine/Pharmacie, F-63000 Clermont-Ferrand, France; 4 Inserm, U1107 Neuro-Dol, F-63001 Clermont-Ferrand, France; 5 CHU Clermont-Ferrand, Pharmacie Hospitalière, secteur Recherche Clinique - 58, rue Montalembert, F-63003 Clermont-Ferrand, France; 6 CHU de Clermont-Ferrand, Délégation Recherche Clinique & Innovation - Villa annexe IFSI, 58 Rue Montalembert, F-63003 Clermont-Ferrand cedex, France; Cardiff University, UNITED KINGDOM

## Abstract

**Background:**

Neuropathic pain following surgical treatment for breast cancer with or without chemotherapy is a clinical burden and patients frequently report cognitive, emotional and quality of life impairment. A preclinical study recently showed that memantine administered before surgery may prevent neuropathic pain development and cognitive dysfunction. With a translational approach, a clinical trial has been carried out to evaluate whether memantine administered before and after mastectomy could prevent the development of neuropathic pain, the impairment of cognition and quality of life.

**Method:**

A randomized, pilot clinical trial included 40 women undergoing mastectomy in the Oncology Department, University Hospital, Clermont-Ferrand, France. Memantine (5 to 20 mg/day; n = 20) or placebo (n = 20) was administered for four weeks starting two weeks before surgery. The primary endpoint was pain intensity measured on a (0–10) numerical rating scale at three months post-mastectomy.

**Results:**

Data analyses were performed using mixed models and the tests were two-sided, with a type I error set at α = 0.05. Compared with placebo, patients receiving memantine showed at three months a significant difference in post-mastectomy pain intensity, less rescue analgesia and a better emotional state. An improvement of pain symptoms induced by cancer chemotherapy was also reported.

**Conclusions:**

This study shows for the first time the beneficial effect of memantine to prevent post-mastectomy pain development and to diminish chemotherapy-induced pain symptoms. The lesser analgesic consumption and better well-being of patients for at least six months after treatment suggests that memantine could be an interesting therapeutic option to diminish the burden of breast cancer therapy.

**Trial Registration:**

Clinicaltrials.gov NCT01536314

## Introduction

Although surgery, radiotherapy and chemotherapy have dramatically increased the life expectancy of patients suffering from breast cancer [1], these treatments may also induce neuropathic pain. In the course of breast surgery, 20–68% of patients report burning and shooting pain localized in the anterior chest, arm and axilla with numbness and pressure sensation [2]. Chronic pain is usually defined as pain lasting longer than 2 to 3 months [3]. Mastectomy is known to generate neuropathic pain in 30.7% patients at 3 months, 25.7% at 6 months [3], 42% at 5 years [4] and 37% at 9 years post-mastectomy [5]. Cancer chemotherapy is also well known to induce pain with neuropathic characteristics in 25–50% of patients [6]. Neuropathic pain is also associated with fatigue, psychosocial distress, diminished quality of life and impaired cognition, and chemotherapy is reported to have a deleterious impact on cognitive-affective processes [7, 8]. All these factors impact negatively on the ability to cope with pain and lead to a potential risk of non-resilience [9]. Recommended drugs such as antidepressants, antiepileptics or opioids may be inefficient or poorly tolerated with their own side-effects [10], especially at central level [11], adding even more to the burden of the disease.

When patients face therapeutic failure with classical drugs, ketamine, a *N*-methyl-D-aspartate (NMDA) receptor (r) antagonist, may be an alternative, but serious side-effects limit its clinical use [12]. The efficacy of memantine, another NMDAr antagonist usually prescribed in Alzheimer’s disease, is also controverted in neuropathic pain alleviation [13–17] but is far better tolerated than ketamine [18]. NMDAr has a pivotal role in central plastic changes and in spinal/cortical potentiation contributing to chronic pain, especially *via* its NR2B-subunit [19]. However, all published studies have so far targeted NMDAr when chronic pain and central sensitization are fully established, with active pain-associated protein expression downstream from NMDAr [20]. Rather than having a reactive attitude to pain by targeting the NMDAr after the insult, our approach is to develop a preventive attitude recommended in the "4P Medicine" [21]. Preemptive protocols with a number of analgesics have been tried to reduce post-surgery pain with contradictory results, and preemptive analgesia was never done earlier than on the day of surgery [22–27].

A recent preclinical study has reported for the first time that memantine administered for four days before surgery in a neuropathic pain model prevents the development of neuropathic pain symptoms and cognitive impairment [28]. Concomitantly, molecular biology showed no downstream protein expression of pTyr^1472^NR2B at spinal and cerebral level, confirming the preventive effect of memantine on central sensitization. This present clinical trial explores the preventive properties of oral memantine on pain and on the cognitive and quality of life impairment up to six months after mastectomy. Considering the poor efficacy of available drugs on post-operative and cancer-induced neuropathic pain, and the risks associated with comorbidity and multiple pharmacy [29], this novel pro-active approach tackling neuropathic pain before its genesis could be a treatment option for the millions of women who suffer from pain associated with breast cancer therapy.

## Materials and Methods

### Study design and patients

The methodology of the study has been detailed in a recent article [30].

We conducted a randomized, single-blind, placebo-controlled clinical trial in the University Oncology Hospital, Clermont-Ferrand, France. The study has been approved in December 2011 by the regional Ethics committee (CPP Sud-Est, France, number AU917) and registered on 16 February 2012 at http://www.clinicaltrials.gov (NCT01536314). The protocol for this trial and supporting CONSORT checklist (S1 and S2 Files) are available as supporting information. Women were eligible if they were at least 18 years old, with a diagnosis of breast cancer, programmed for mastectomy with or without axillary dissection, able to understand and willing to follow the study protocol. Exclusion criteria comprised contra-indications for memantine and hypertension, severe cardiac insufficiency or diabetes (Type I and II), alcohol addiction and treatment with specific drugs (amantadine, ketamine, dextromethorphan, L-Dopa, dopaminergic, anticholinergic agonists, barbituric, neuroleptic, IMAO, antispastic agents, dantrolen or baclofen, phenytoin, cimetidine, ranitidine, procainamide, quinidine, quinine, nicotine, hydrochlorothiazide, warfarine). Other exclusion criteria were childbearing age, no use of an effective contraceptive method, pregnancy or lactation, involvement in another clinical trial and inability to comply with the requirements of protocol.

Before giving informed consent, patients were informed that they would be participating voluntarily and that they could chose to withdraw at any time. Furthermore, the general aims, the different questionnaires and the pharmacological intervention of the study were explained to each participant.

Women were included two weeks before mastectomy (Baseline), in the course of their Anesthesiology visit. After clinical examination, pain, cognition, quality of life and sleep questionnaires were filled out and patients were randomized in two parallel groups: memantine (n = 20) or placebo (n = 20). Oral treatment was given for four weeks starting two weeks before surgery (S). Memantine was prescribed according to regional recommendations for Alzheimer disease. Follow-up clinical examination was performed 2 weeks (S+15 days), 3 and 6 months after mastectomy (M3 and M6). Patients were called once a week by phone to collect adverse events. A booklet for monitoring analgesic concomitant medications was completed daily by the patient for six months from the day of surgery.

Pain was evaluated with several tools: a (0–10) numerical rating scale (NRS), the Brief Pain Inventory (BPI) [31], the McGill pain questionnaire [32], the Neuropathic Pain questionnaire in four questions (DN4) [33] and the Neuropathic Pain Symptom Inventory (NPSI) [34]. Cognition was evaluated by the Trail Making Test (TMT) [35, 36] and the Digit Symbol Substitution Test (DSST) [37]. Sleep was assessed by the Leeds Sleep Questionnaire [38] and Quality of Life by the Short-Form health survey (SF-36) questionnaire [39, 40].

Furthermore, we differentiated neuropathic pain induced by surgery, "surgery DN4" focused on the surgery site, from neuropathic pain induced by chemotherapy, "chemotherapy DN4" focused on "hand and foot" distribution of chemotherapy-induced neuropathy.

### Intervention

#### Treatment group

The treatment group received memantine during one month, starting two weeks before surgery. Memantine was prescribed in increasing doses during the first two weeks before mastectomy and maintained at 20 mg daily during two weeks after surgery.

#### Control group

Patients received a daily dose of placebo (lactose) during one month starting two weeks before mastectomy.

The drugs used in the study (memantine and placebo) were prepared, conditioned and released in the hospital pharmacy by one qualified person according to good manufacturing principles. The number of tablets in each dispensed container was verified and recounted at the end of the treatment by two persons totally independent of the protocol.

### Outcomes measures

The primary outcome was to evaluate by NRS whether memantine, administered two weeks before and two weeks after mastectomy, could prevent pain development at three months post-mastectomy when compared with the placebo group.

The secondary outcomes were to evaluate at three and six months post-mastectomy the pain intensity, the analgesic concomitant medications, the impact of treatment on cognitive function, quality of life and sleep and the intensity of cancer chemotherapy-induced pain.

### Sample size

The estimated number [30] of patients required for the study was 40 (n = 20 in each group). The minimum difference in NRS pain evaluation between memantine and placebo groups at three months was 1.6 (standard deviation equals 1.5), estimated from published data [41, 42], with two-sided type I error α = 0.05 and β = 0.10.

### Randomization, allocation and masking of study groups

On the day of the visit, inclusion and exclusion criteria were verified and written informed consent was obtained by the physician. After clinical examination and pain assessment, a clinical nurse independent of the protocol obtained the randomization number from the hospital pharmacy and the patient was then randomized in the memantine or placebo group. Treatment allocation followed the order of a predetermined randomization list and was generated using random blocks. In order to maintain blinding, the physician who evaluated pain could not guess allocation at any time and would not meet the patient again in the course of the trial.

### Statistical analysis

Statistical analyses were performed with Stata software (version 13; StataCorp, College Station, US). The tests were two-sided, with a type I error set at α = 0.05. Concerning the primary outcome, comparison between the randomized groups was performed using an analysis of covariance with baseline score as a covariate as suggested by Vickers and Altman [43]. Concerning the secondary outcomes, the comparisons between the randomized groups were carried out using Student’s *t*-test or Mann-Whitney test when appropriate (normality verified by Shapiro-Wilk and homoscedasticity by Fisher-Snedecor tests) for quantitative parameters and using chi-squared or Fisher’s exact tests. For the primary endpoint, results were expressed as effect-size and 95% confidence interval. Concerning the analysis of repeated measures, random-effect models were considered, as it was usually proposed, to study fixed effects (group, time-points and interaction group × time) and taking into account between and within subject variability. The normality of residuals was checked for each model. When appropriate, a log transformation was proposed to achieve the normality of dependent variables. Subgroups analyses were planned and were performed with anticancer chemotherapy (yes/no) proposed as fixed effect in previous models. Residual normality was checked for all models. Finally, a sensitivity analysis was carried out to study the attrition bias and to characterize the statistical nature of missing data.

## Results

**Fig 1:**
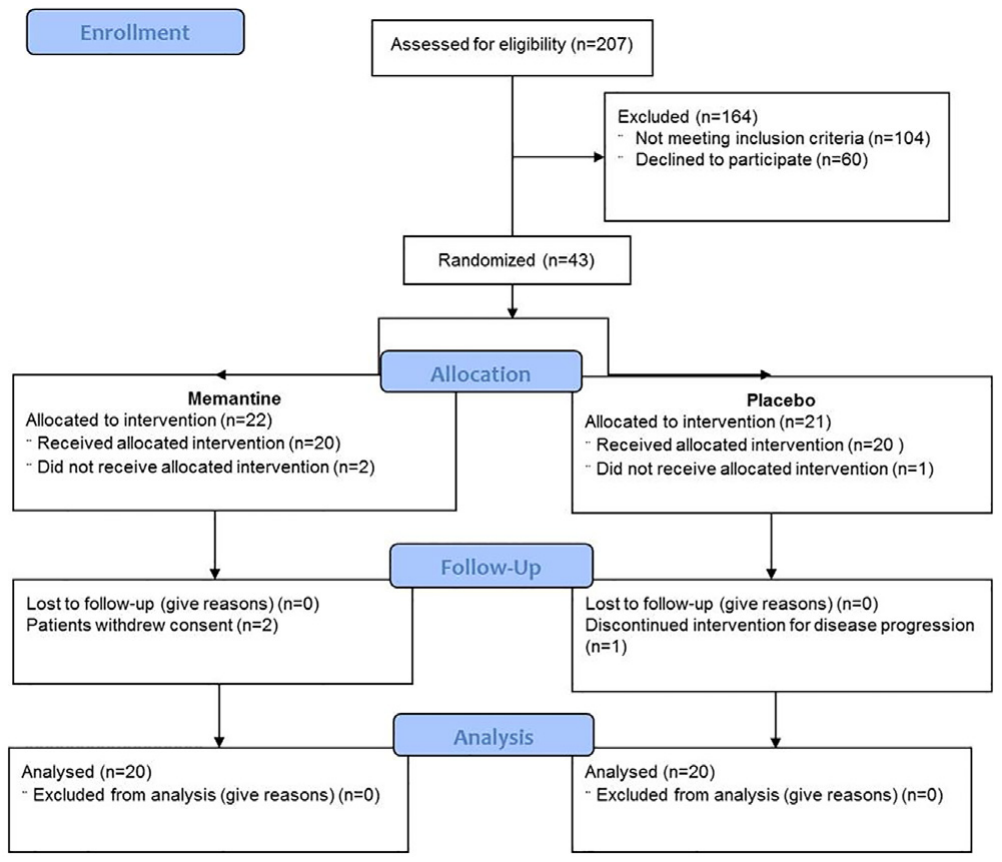
Flowchart of participants during the trial.

The investigators pre-screened 207 patients; 104 women refused to participate in the study, 60 did not meet the inclusion criteria and 43 gave written informed consent. These were randomized into the memantine or placebo group. Two patients withdrew before starting treatment and one interrupted the trial because surgery was postponed due to advanced disease. Out of 43 enrolled patients, 40 were analysed (n = 20 in the memantine group and n = 20 in the control group; Fig 1). The investigation was carried out from March 2012 to April 2013 for recruitment, and the follow-up finished in November 2013. No adverse events have been reported in this study and no patient dropped out during the six months after mastectomy.

Two hundred and seven patients have been assessed for eligibility; 104 women refused to participate in the study, 60 did not meet the inclusion criteria and 43 gave written informed consent. These were randomized into the memantine or placebo group. Two patients withdrew before starting treatment and one interrupted the trial because surgery was postponed due to advanced disease. Out of 43 enrolled patients, 40 were analysed (n = 20 in the memantine group and n = 20 in the control group).

Demographic and clinical data of the 40 participants, including age, previous chemotherapy, type of anticancer agents and axillary dissection are presented in Table 1.

**Table 1:** Demographics and clinical characteristics.

	General population	Memantine group	Placebo group	P-value
Demographic and clinical data	n = 40	n = 20	n = 20	
**Age (mean [min, max])**	54.4 [33, 71]	51.6 [33, 71]	57.3 [38, 70]	0,09
**Previous chemotherapy**	**n (%)**	**n (%)**	**n (%)**	
yes	21 (52.5)	11 (55.0)	10 (50.0)	0,75
**Type of chemotherapy**	**n (%)**	**n (%)**	**n (%)**	
Spindle poisons (*Taxotere*)	21 (100)	11 (55.0)	10 (50.0)	0,75
Anti-metabolites (*5-Fluorouracil*)	18 (85.7)	9 (45.0)	9 (45.0)	1,00
Alkylating agent (*Endoxan/Carboplatin*)	20 (95.2)	10 (50.0)	10 (50.0)	1,00
Intercalating agent (*Epirubicin*)	18 (85.7)	9 (45.0)	9 (45.0)	1,00
monoclonals Antibodies (*Herceptin*)	2 (5.0)	1 (5.0)	1 (5.0)	1,00
**Axillary dissection**	**n (%)**	**n (%)**	**n (%)**	
yes	19 (47.5)	9 (45.0)	10 (50.0)	0,75

The median age in both groups was 54.4 years (54.4 ± 10.4) at study entry, 21 (52.5%) had received previous chemotherapy; 19 (47.5%) had an axillary dissection. Anticancer chemotherapy included fluorouracil, epirubicin and cyclophosphamide (FEC) in 86% patients and Docetaxel in 100%. No statistically significant difference between groups in any sociodemographic or clinical variable was obtained, indicating that both groups were equivalent for the variables measured.

At M3 post-mastectomy, a significant difference in the primary outcome, NRS pain intensity, was recorded in the memantine group compared with the placebo group (placebo: 1.3 ± 1.8; memantine: 0.2 ± 0.4; p = 0.017) while at M6 post-mastectomy no significant difference was observed (placebo: 0.9 ± 2.0; memantine: 0.5 ± 0.8; p = 0.10). The effect size for the main end point was 0.76 (95% CI: [0.12; 1.40]).

**Fig 2:**
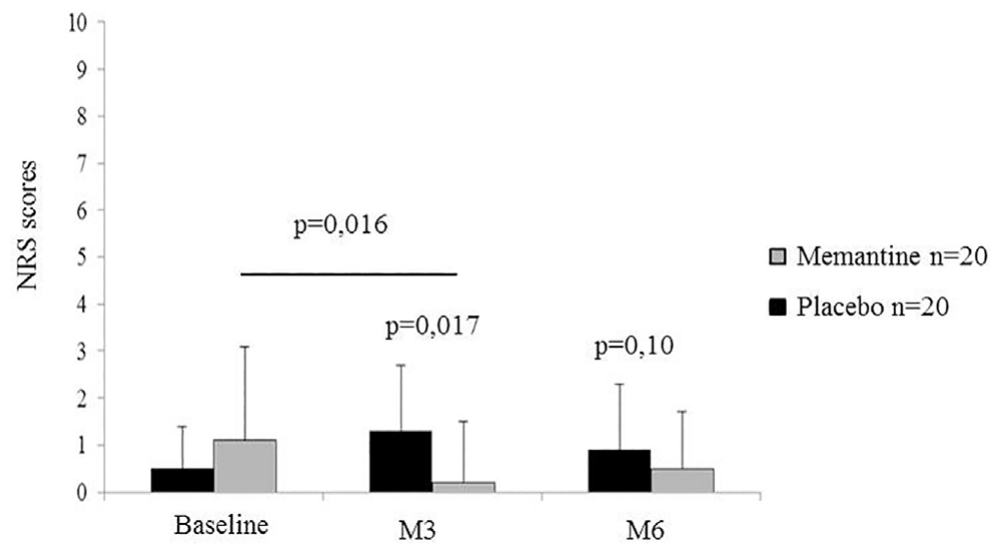
Effect of memantine on overall pain evaluated by numerical rating scale. A significant difference was obtained with the Numerical Rating Scale (NRS) at Month 3 post-mastectomy in the memantine group (n = 20) compared with the placebo group (n = 20) (p = 0.017). No significant difference was reported at Month 6 between the two groups. A significant decrease was also reported at M3 in the memantine group compared with baseline (p = 0.016) but such diminution in the same group was not observed at M6 post-mastectomy.

Regarding the secondary outcomes, a significant decrease of pain intensity was reported in the memantine group at M3 compared with baseline (memantine, Baseline: 1.2 ± 2.0; M3: 0.2 ± 0.4; p = 0.016) but such a decrease was not obtained at M6 post-mastectomy (Fig 2).

Concerning neuropathic pain induced by surgery (DN4 surgery), no significant difference was observed at M3 and M6 post-mastectomy between memantine and placebo groups. In the placebo group, 45% (n = 9) of patients at M3 and 30% (n = 6) at M6 had neuropathic pain while in the memantine group, 35% (n = 7) developed neuropathic pain at M3 and M6 (Table 2). In the placebo group, six had a DN4 score ≥ 4 with values between 5 to 8 while in the memantine group, only two patients had a score of 5.

**Table 2:** Effect of memantine on neuropathic pain induced by mastectomy.

	Follow-up	Placebo	Memantine	P-value
**DN4 surgery**	Baseline	1.1 ± 1.4	0.6 ± 1.2	0,18
	S	2.8 ± 1.9	3.0 ± 2.0	0,25
	S+15 days	3.4 ± 1.7	2.8 ± 2.1	0,87
	S+ 3 months	3.6 ± 2.1	2.8 ± 1.5	0,68
	S+ 6 months	2.6 ± 2.2	2.6 ± 1.7	0,37
**DN4 ≥ 4**	S+ 3 months	n = 9 (45%)	n = 7 (35%)	0,52
	S+ 6 months	n = 6 (30%)	n = 7 (35%)	0,74

Using the neuropathic pain questionnaire in four questions (DN4), no significant difference was observed between the memantine and placebo groups at Months 3 and 6. Concerning the proportion of patients who developed neuropathic pain or characteristics of neuropathic pain in the memantine and placebo groups at Months 3 and 6, no significant difference was obtained between two groups.

**Fig 3:**
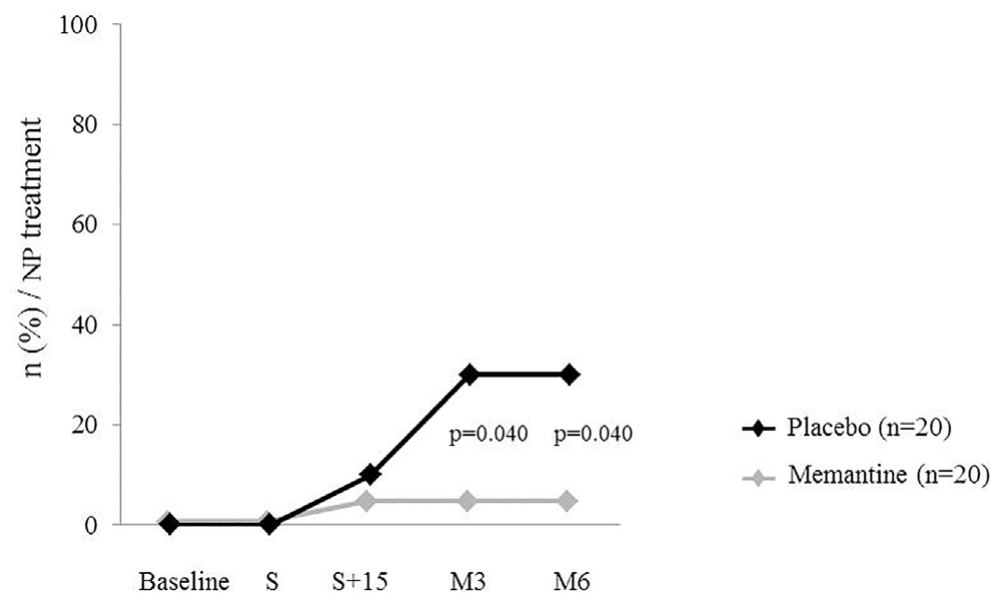
Effect of memantine on analgesics consumption. Number of patients n (%) being prescribed neuropathic pain analgesics. A significant increase in analgesics (especially antiepileptics) prescriptions was reported in the placebo group (n = 20) compared with the memantine group (n = 20) at Month 3 and maintained at Month 6 (p = 0.040).Over all time different was significant (p = 0.041).

Concerning analgesics, all patients were prescribed non-opioids (paracetamol, non-steroidal anti-inflammatory drugs), with opioids for two days post-mastectomy. At M3, there was a significant difference in neuropathic pain drug consumption (antiepileptics prescribed for pain) between both groups, (six patients in the placebo group (30%) and only one patient (5%) in the memantine group; p = 0.040) (Fig 3). This difference was maintained at M6 (p = 0.040) (Fig 3). The over all time difference was significant (p = 0.041). Patients in the memantine group reported not needing analgesics. Details of analgesic prescriptions are reported in Table 3.

**Table 3:** Detail of analgesics consumption after mastectomy.

	Total sample	Memantine group	Placebo group	P-value
	n = 40	n = 20	n = 20	
**S to S + 15 days**	n (%)	n (%)	n (%)	
**Step 1 analgesics** (Paracetamol, NSAIDs)	40 (100)	20 (100)	20 (100)	1,00
**Step 2 analgesics** (Tramadol)	16 (40.0)	7 (35.0)	9 (45.0)	0,52
**Step 3 analgesics** (Morphine)	30 (75.0)	15 (75.0)	15 (75.0)	1,00
**S + 15 days to S + 3 months**	n (%)	n (%)	n (%)	
**Step 1 analgesics** (Paracetamol, NSAIDs)	22 (55.0)	11 (55.0)	11 (55.0)	1,00
**Step 2 analgesics** (Tramadol)	9 (22.5)	5 (25.0)	4 (20.0)	0,71
**Step 3 analgesics** (Morphine)	1 (2.5)	0 (0.0)	1 (5.0)	>0,99
**S + 3 months to S + 6 months**	n (%)	n (%)	n (%)	
**Step 1 analgesics** (Paracetamol, NSAIDs)	9 (22.5)	3 (15.0)	6 (30.0)	0,26
**Step 2 analgesics** (Tramadol)	2 (5.0)	1 (5.0)	2 (10.0)	0,55
**Step 3 analgesics** (Morphine)	0 (0.0)	0 (0.0)	0 (0.0)	1,00

Analgesics are classified according to nociceptive pain treatment (Step 1: paracetamol, non-steroidal anti-inflammatory drugs (NSAIDs), opioids: Step 2: tramadol and Step 3: morphine. The periods are: between 1) the day of surgery (S) and 15 days post-mastectomy (S to S+15 days), 2) 15 days and 3 months post-surgery (S+15 days to S+3 months) and 3) 3 months and 6 months post-mastectomy (S+3 months to S+6 months). No significant difference was obtained in analgesic consumption between the placebo group (n = 20) and the memantine group (n = 20).

**Fig 4:**
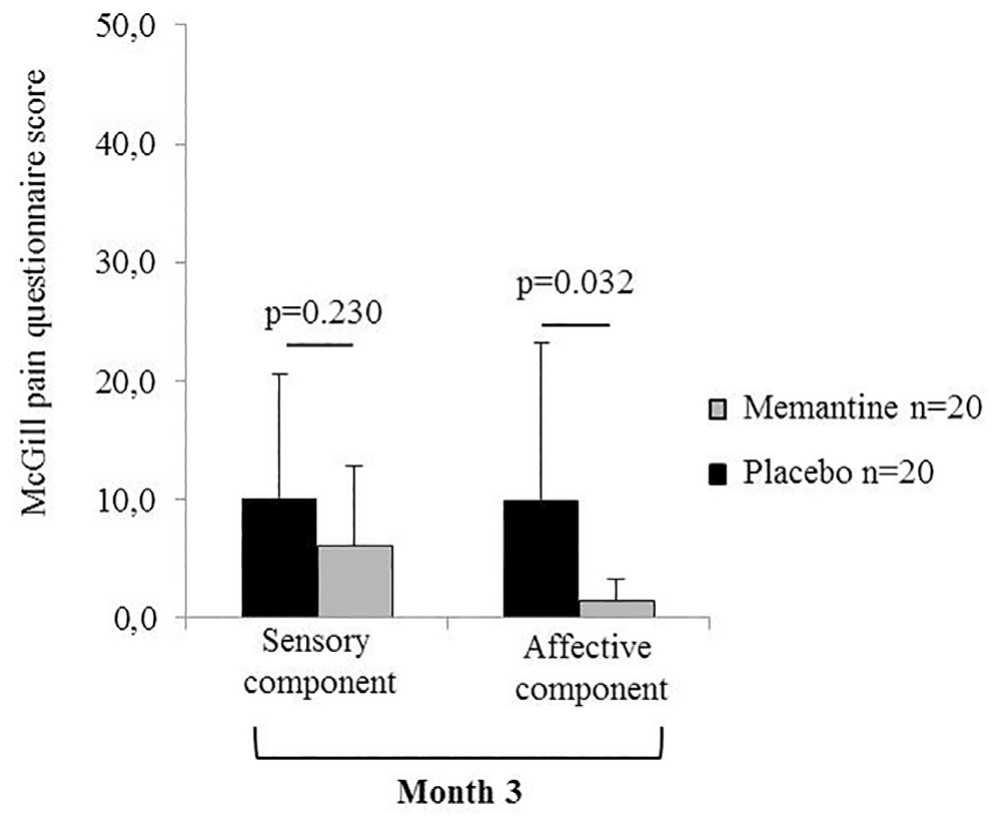
ffect of memantine on the affective component of pain evaluated by the McGill pain questionnaire. A significant difference was reported in the memantine group (n = 20) compared with the placebo group (n = 20) at Month 3 (p = 0.032).

In the McGill pain questionnaire, the affective component was improved at three months (placebo: 10.0 ± 13.2; memantine: 1.4 ± 1.9; p = 0.032) in the memantine group (Fig 4) while no significant difference was demonstrated in the other pain questionnaires (Table 4).

**Table 4:** Effect of memantine on the Neuropathic Pain Symptom Inventory (NPSI) and the Brief Pain Inventory questionnaires (BPI: pain severity; REM: Relation with other, Enjoyment of life, Mood; WAW: Walking, general Activity, Working; patient pain experience).

	Follow-up	Placebo	Memantine	P-value
**NPSI**	S+ 3 months	8.5 ± 12.2	5.2 ± 7.5	0,36
	S+ 6 months	5.4 ± 9.4	2.7 ± 3.1	0,47
**BPI**: Pain severity	S+ 3 months	0.9 ± 1.7	0.6 ± 1.2	0,47
	S+ 6 months	0.9 ± 2.1	0.6 ± 1.1	0,96
**BPI**: REM	S+ 3 months	0.6 ± 1.3	0.1 ± 0.4	0,38
	S+ 6 months	0.6 ± 1.6	0.2 ± 0.6	0,89
**BPI**: WAW	S+ 3 months	1.4 ± 2.3	1.2 ± 1.7	0,94
	S+ 6 months	0.9 ± 1.8	0.7 ± 1.5	0,96
**BPI**: Patient pain experience	S+ 3 months	1.0 ± 1.5	0.7 ± 1.0	0,66
	S+ 6 months	0.9 ± 2.1	0.5 ± 1.0	0,84

No significant difference was reported between the memantine and placebo groups.

**Fig 5:**
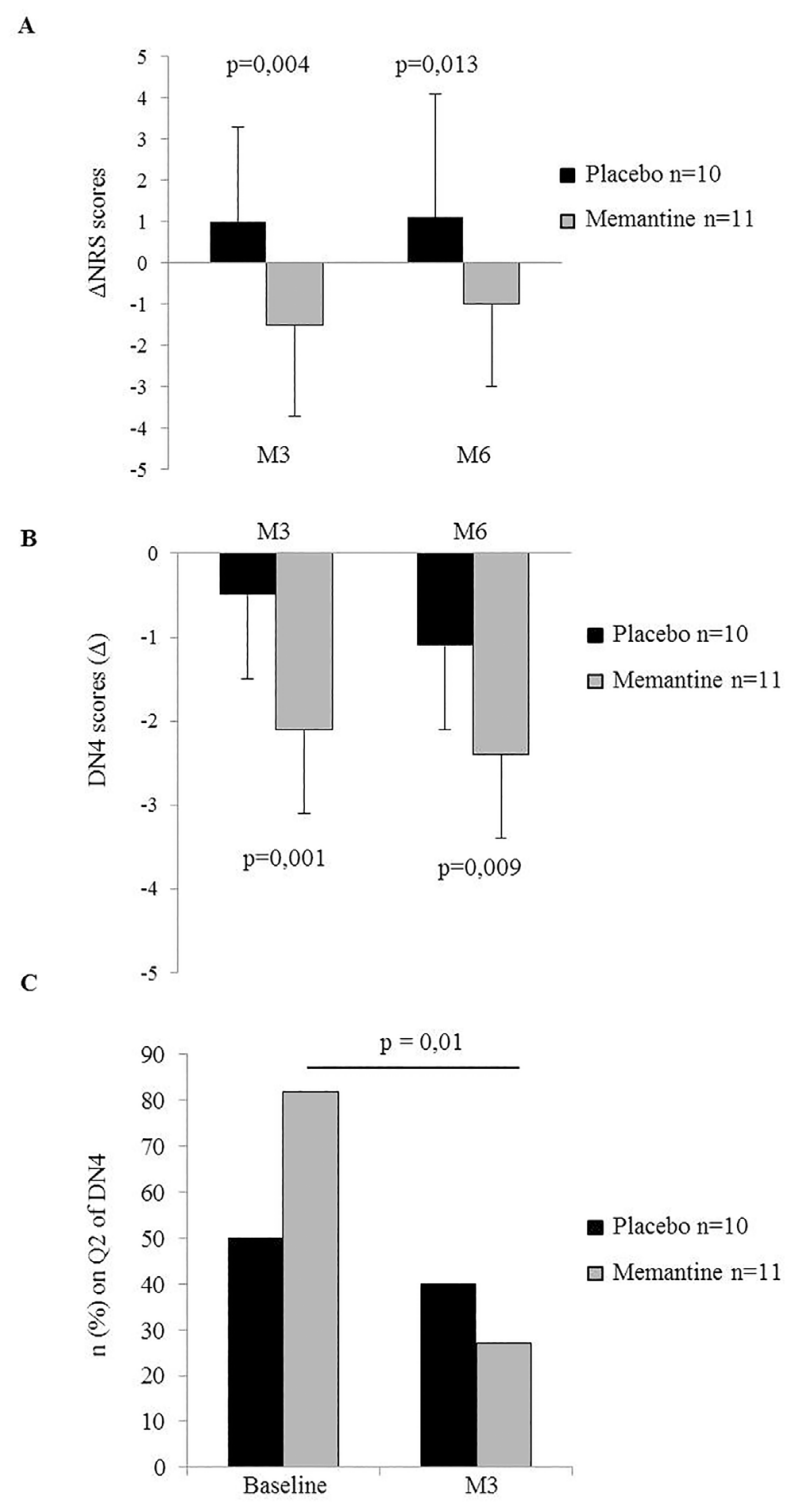
Effect of memantine on pain in patients who had chemotherapy. (A) ΔNRS score is the pain intensity difference between Month 3 or Month 6 and baseline. It is significant in the subgroup of chemotherapy which received memantine (n = 11) compared with placebo (n = 10) at Month 3 (p = 0.01) and at Month 6 (p = 0.01). (B) Neuropathic pain (ΔDN4 score) is the neuropathic pain score difference between Month 3 or Month 6 and baseline. Neuropathic pain score in four questions was significantly diminished in the memantine group at Month 3 (***p = 0.001) and at Month 6 (p = 0.009). (C) Number of patients n (%) who replied positively to question 2 (Q2) of DN4 (dysesthesias and paresthesias). In the memantine group, a decrease of 55% of dysesthesias and paresthesias was reported at Month 3 compared with the day of inclusion (Baseline) (p = 0.01).

Of the forty patients included in the study (n = 20 in each group of treatment), half of the patients had received chemotherapy before inclusion (memantine: n = 11; placebo: n = 10). In this subgroup, at M3 and M6, pain was significantly less intense in the memantine group (ΔNRS, M3, placebo: 1.0 ± 2.3; memantine: -1.5 ± 2.2; p = 0.004. M6, placebo: 1.2 ± 3.2; memantine: -1.2 ± 2.0; p = 0.013, Fig 5A). However, the interaction ‘anticancer chemotherapy x randomized’ was not statistically significant for this parameter (ΔNRS p = 0.06).

In the memantine group, characteristics of neuropathic pain induced by chemotherapy (DN4 chemotherapy) were significantly diminished (ΔDN4 chemotherapy, M3, placebo: -0.5 ± 0.8; memantine: -2.1 ± 1.6; p = 0.001. M6, placebo: -1.0 ± 1.3; memantine: -2.4 ± 2.0; p = 0.009, Fig 5B), but the interaction ‘anticancer chemotherapy x randomized’ was not statistically significant for this parameter (ΔDN4 chemotherapy, p = 0.73). Furthermore, a decrease of 55% of chemotherapy-induced paresthesia and dysesthesia was observed at M3 compared with the day of inclusion (Baseline, memantine: n = 9 (82%); M3: n = 3 (27%); p = 0.01) while in the placebo group, no difference was reported (Baseline, placebo: n = 5 (50%); M3: n = 4 (40%) p = 0.32) (Fig 5C).

Concerning cognitive tests (Table 5) and quality of life (Table 6), no significant difference was observed between placebo and memantine groups at M3 and M6.

**Table 5:** Effect of memantine on cognition.

	Follow-up	Placebo	Memantine	P-value
**DSST 90''**	Baseline	48.9 ± 9.5	53.8 ± 15.7	0,29
	S+ 3 months	54.6 ± 9.9	60.8 ± 15.9	0,78
	S+ 6 months	55.6 ± 10.8	58.6 ± 15.2	0,36
**DSST 120''**	Baseline	69.0 ± 13.7	72.9 ± 20.2	0,28
	S+ 3 months	74.5 ± 11.9	79.9 ± 15.8	0,82
	S+ 6 months	76.2 ± 13.6	78.8 ± 16.5	0,58
**TMT A**	Baseline	35.6 ± 11.3	35.5 ± 12.0	0,87
	S+ 3 months	29.8 ± 6.8	30.5 ± 10.7	0,48
	S+ 6 months	31.5 ± 7.9	32.9 ± 13.8	0,61
**TMT B**	Baseline	88.5 ± 29.3	68.9 ± 21.3	0,19
	S+ 3 months	82.7 ± 28.8	71.9 ± 39.3	0,26
	S+ 6 months	85.1 ± 26.3	63.6 ± 23.0	0,87

Means of the DSST (Digit Symbol Substitution Test) and TMT (Trail Making Test) scores were expressed in seconds. No significant difference was reported between the memantine and placebo groups, whatever the questionnaire used.

**Table 6:** Effect of memantine on quality of life by SF-36 (Short Form 36).

SF-36	Follow-up	Placebo	Memantine	P-value
Physical Health	Baseline	73.1 ± 17.5	72.0 ± 20.2	0,66
	S+ 3 months	65.8 ± 19.2	67.2 ± 19.6	0,81
	S+ 6 months	76.3 ± 19.5	76.7 ± 17.6	0,82
Mental Health	Baseline	70.2 ± 19.2	72.3 ± 18.2	0,70
	S+ 3 months	65.8 ± 19.2	75.0 ± 14.9	0,52
	S+ 6 months	76.3 ± 19.5	80.1 ± 14.2	0,87

No statistically significant difference was shown between the memantine and placebo treatment groups, in any of the domains.

Furthermore, no significant difference was demonstrated at M3 and M6 for the Leeds sleep questionnaire but for the item "behavior following wakefulness" at M6, where an improvement of this dimension was reported for memantine compared with baseline (Baseline, 6.8 ± 3.8 *versus* 4.6 ± 5.5; M6, 4.8 ± 4.9 *versus* 5.8 ± 3.8; p = 0.038) (Table 7).

**Table 7:** Effect of memantine on quality of sleep with Leeds sleep questionnaire.

Leed's sleep questionnaire	Follow-up	Placebo	Memantine	P-value
Sleep latency	Baseline	3.8 ± 6.8	3.5 ± 8.2	0,978
	S+ 3 months	4.0 ± 6.9	4.2 ± 7.7	0,813
	S+ 6 months	4.9 ± 5.6	6.3 ± 6.9	0,438
Quality of sleep	Baseline	1.1 ± 5.4	0.3 ± 6.2	0,734
	S+ 3 months	0.0 ± 5.8	1.6 ± 5.9	0,256
	S+ 6 months	1.0 ± 6.0	2.0 ± 5.0	0,372
Awakening from sleep	Baseline	7.9 ± 5.6	6.8 ± 6.2	0,614
	S+ 3 months	4.0 ± 5.2	6.3 ± 6.9	0,149
	S+ 6 months	6.2 ± 7.4	5.0 ± 5.1	0,983
Behavior following wakefulness	Baseline	6.8 ± 3.8	4.6 ± 5.5	0,190
	S+ 3 months	5.8 ± 3.9	5.2 ± 5.0	0,316
	S+ 6 months	4.8 ± 4.9	5.8 ± 3.8	0,038

A significant difference was observed at Month 6 between the memantine and placebo groups with the item "behavior following wakefulness" (p = 0.038). No significant difference was reported with other items.

## Discussion

This pilot trial proposes for the first time with the administration of the NMDAR blocker memantine before surgery, a successful therapeutic option to prevent pain and diminish neuropathic pain treatment. Three months post-mastectomy, patients in the memantine group reported significantly less pain than in the placebo group (p = 0.017) and only 5% needed neuropathic pain treatment compared with 30% in the placebo group (p = 0.04). No such a randomised successful intervention preventing neuropathic pain development after surgery has been shown so far in the literature. A number of analgesics have been tested to reduce pain after breast surgery but data of the literature are conflicting and focus on acute rather than long—term residual chronic pain [22–27].

The study also showed that patients coped better with pain, as shown by the beneficial effect of memantine on the emotional component of pain using the McGill pain questionnaire (p = 0.032), and they declared to the physician not to be bothered by pain. However, in both groups, a third of the patients had significant neuropathic pain scores (DN4≥4). This paradox between the presence of pain and the indifference of the patient to it may suggest a sensori-limbic dissociation in the effect of memantine, with features reminiscent of pain asymbolia [44]. Such a sensory-limbic dissociation has previously been suggested in patients with long-standing neuropathic pain, who were prescribed one month oral treatment with magnesium, a physiological blocker of NMDAr [45]. A key point of this trial was that the emotional component of pain was improved and patients were feeling better despite the presence of pain. NMDAr are largely distributed in the brain especially in the hippocampus [46], a pivotal area of memantine action for cognitive/memory processes in Alzheimer’s disease and also for initiation of long-term potentiation (LTP), in the anterior cingulate cortex (ACC) and in the forebrain with a probable impact on the affective quality of pain [47].

This clinical trial does confirm preclinical results where memantine given for several days before surgery has been shown to limit and even inhibit downstream protein expression and phosphorylation of tyrosine 1472 on the NR2B subunit of NMDAr in two structures of the limbic system, the hippocampus and the insular cortex [28]. These collective results suggest that circulating memantine prevents post-surgery pain by inhibiting the development of central sensitization in emotional memory of pain by blocking post-translational modifications such as protein phosphorylation on NMDAr of the limbic system, a major mechanism for the regulation of NMDAR. Furthermore, Li et al., 2014 [48], recently showed for the first time that the temporal precision of information within thalamic-cingulate pathways was decreased after peripheral injury in an animal neuropathic pain model. They also observed changes in neuronal properties involving the glutamatergic synaptic transmission that would reinforce the pivotal role of NMDAr in the interaction of cognition and neuropathic pain.

For the first time, this pilot trial also reports a curative effect of memantine on chemotherapy-induced neuropathic pain at M3 and M6, with a 55% reduction of dysesthesia and paresthesia symptoms in patients who had received chemotherapy before inclusion and had developed chemotherapy-induced typical polyneuropathy with "hand and foot" distribution [6]. Although this is a secondary endpoint, this finding of alleviation of pain will help to build future studies, considering that apart from the reduction or discontinuation of chemotherapy, no specific pain treatment option is today available and efficacious for these patients.

Likewise, cognition, sleep and quality of life were also studied in the trial, as these domains are often impaired in cancer patients because of multifactorial and intertwined causes including cancer itself [8], treatments and chronic pain [49, 50]. No significant difference was shown between the memantine and placebo groups for these secondary endpoints. Concerning cognition, about a hundred patients are usually necessary to show a significant difference between treated and controlled groups [51]. Although the large literature published on the impact of chemotherapy and surgery in breast cancer does not provide universal results, a number of publications agree with our results when using neuropsychological tests [52–53]. A recent meta-analysis [8] has shown that objective tests in a longitudinal study tend to show no impairment of cognition because of training with the repetition of tests and development of compensatory strategies by the patient [52]. Conversely, subjective tests or transversal data show diminished cognition in 20–75% patients [54] and are suggested to be more reliable than longitudinal data [9]. A study conducted in 348 subjects could detect no alterations in cognition in patients with breast cancer undergoing surgery. Although neuropsychological tests reported no damage, 60% of patients had impaired memory, concentration and vitality [55].

Similarly, assessment of sleep and quality of life, secondary endpoints, did not show any significant difference between both groups. This is certainly linked to the small sample size and the overall short duration of the study. Neuropathic pain is known to affect quality of life, functionality, and to precipitate functional decline especially in vulnerable patients [56], and future studies should focus on these aspects.

In conclusion, this innovative trial shows for the first time that pre-surgery memantine may prevent the occurrence of pain three months after mastectomy, and suggests that it may also reduce dysesthesia and paresthesia induced by chemotherapy. Memantine could be a valuable option for patients with breast cancer who are at risk of developing the double burden of post-mastectomy and post-chemotherapy neuropathic pain, both potentially long-lasting types of pain. This pilot trial will help to design future studies and these preliminary results will need to be extended and confirmed over a longer follow-up period, with different memantine dosages and with a larger assessment of pain, cognitive-emotional status and functionality in a larger population.

## Supporting Information

S1 File CONSORT Checklist. CONSORT Checklist of the present study.
(DOC)

S2 File Trial Protocol of the study. Prevention of post-mastectomy neuropathic pain with memantine: study protocol for a randomized controlled trial.
(PDF)
