# Supplementary material for: Memantine before Mastectomy Prevents Post-Surgery Pain: A Randomized, Blinded Clinical Trial in Surgical Patients
Source: PLoS One. 2016 Apr 6;11(4):e0152741. doi: 10.1371/journal.pone.0152741 (PMC4822967; doi:10.1371/journal.pone.0152741)
Supplement: S1 File — CONSORT Checklist of the present study. (DOC) [file pone.0152741.s001.doc]

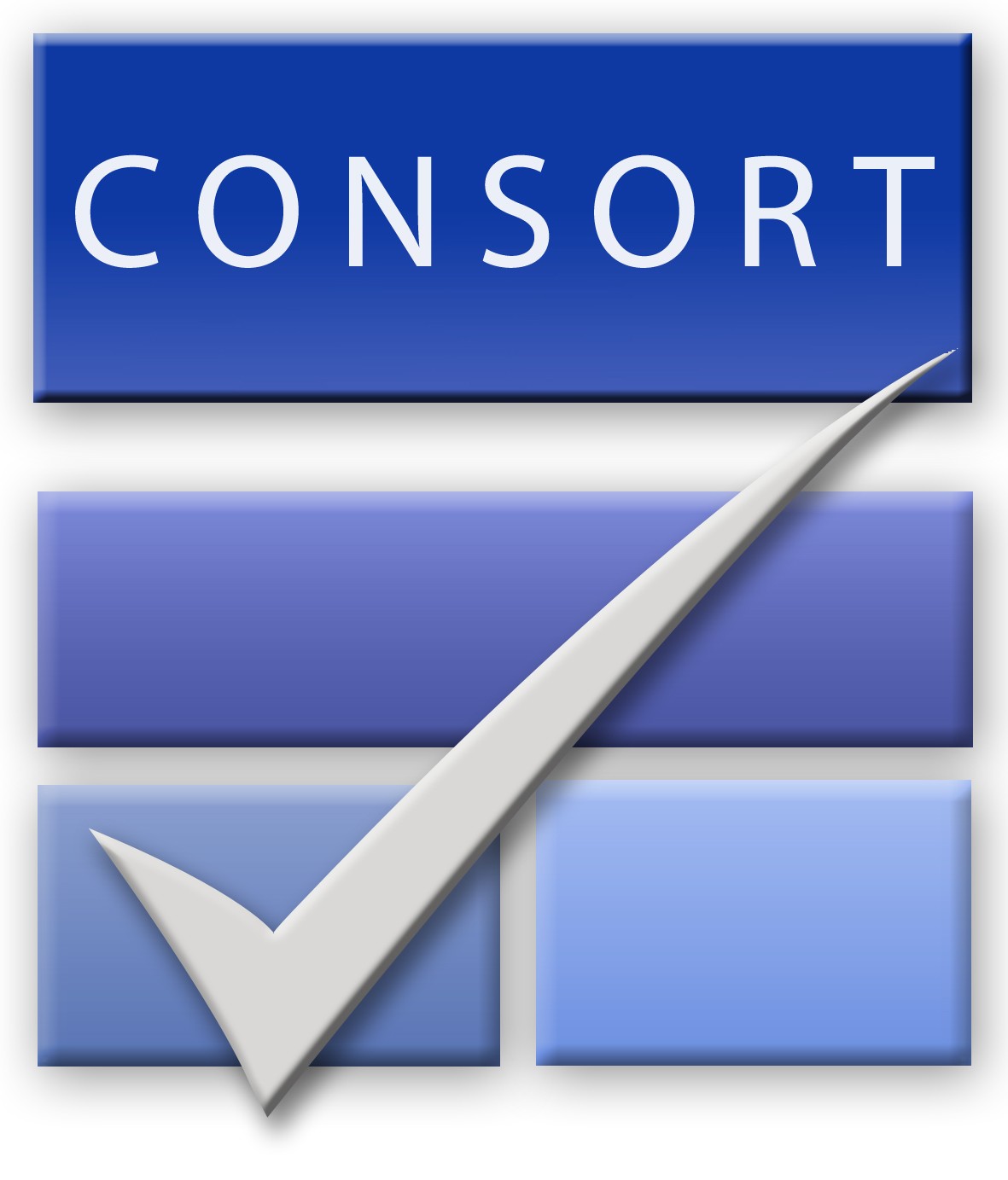
CONSORT 2010 checklist of information to include when reporting a randomised trial*

| Section/Topic | Item No | Checklist item | Reported on page No |
| --- | --- | --- | --- |
| Title and abstract | | | |
|  | 1a | Identification as a randomised trial in the title | Title p1 |
|  | 1b | Structured summary of trial design, methods, results, and conclusions (for specific guidance see CONSORT for abstracts) | Abstract p3 |
| Introduction | | | |
| Background and objectives | 2a | Scientific background and explanation of rationale | Introduction p5 |
|  | 2b | Specific objectives or hypotheses | Introduction p5-6 |
| Methods | | | |
| Trial design | 3a | Description of trial design (such as parallel, factorial) including allocation ratio | Study design and patients p7 |
|  | 3b | Important changes to methods after trial commencement (such as eligibility criteria), with reasons | Study design and patients p7 |
| Participants | 4a | Eligibility criteria for participants | Study design and patients p7 |
|  | 4b | Settings and locations where the data were collected | Study design and patients p7-8 |
| Interventions | 5 | The interventions for each group with sufficient details to allow replication, including how and when they were actually administered | Intervention (Treatment group and control group) p8-9 |
| Outcomes | 6a | Completely defined pre-specified primary and secondary outcome measures, including how and when they were assessed | Outcomes measures p9 |
|  | 6b | Any changes to trial outcomes after the trial commenced, with reasons | Outcomes measures p9 |
| Sample size | 7a | How sample size was determined | Sample size p9 |
|  | 7b | When applicable, explanation of any interim analyses and stopping guidelines | - |
| Randomisation: |  |  |  |
| Sequence generation | 8a | Method used to generate the random allocation sequence | Randomization, allocation and masking of study groups  Randomization p9 |
|  | 8b | Type of randomisation; details of any restriction (such as blocking and block size) | Randomization p9 |
| Allocation concealment mechanism | 9 | Mechanism used to implement the random allocation sequence (such as sequentially numbered containers), describing any steps taken to conceal the sequence until interventions were assigned | Randomization p9 |
| Implementation | 10 | Who generated the random allocation sequence, who enrolled participants, and who assigned participants to interventions | Randomization p10 |
| Blinding | 11a | If done, who was blinded after assignment to interventions (for example, participants, care providers, those assessing outcomes) and how | - |
|  | 11b | If relevant, description of the similarity of interventions | Statistical analysis p10 |
| Statistical methods | 12a | Statistical methods used to compare groups for primary and secondary outcomes | Statistical analysis p10 |
|  | 12b | Methods for additional analyses, such as subgroup analyses and adjusted analyses | - |
| Results | | | |
| Participant flow (a diagram is strongly recommended) | 13a | For each group, the numbers of participants who were randomly assigned, received intended treatment, and were analysed for the primary outcome | Results & Figure1 p11 |
|  | 13b | For each group, losses and exclusions after randomisation, together with reasons | Results & Figure 1 p11 |
| Recruitment | 14a | Dates defining the periods of recruitment and follow-up | Results p11 |
|  | 14b | Why the trial ended or was stopped | Results p11 |
| Baseline data | 15 | A table showing baseline demographic and clinical characteristics for each group | Results & Table 1 p11 |
| Numbers analysed | 16 | For each group, number of participants (denominator) included in each analysis and whether the analysis was by original assigned groups | - |
| Outcomes and estimation | 17a | For each primary and secondary outcome, results for each group, and the estimated effect size and its precision (such as 95% confidence interval) | Results p12-17  - |
|  | 17b | For binary outcomes, presentation of both absolute and relative effect sizes is recommended | - |
| Ancillary analyses | 18 | Results of any other analyses performed, including subgroup analyses and adjusted analyses, distinguishing pre-specified from exploratory | Results p12-17 |
| Harms | 19 | All important harms or unintended effects in each group (for specific guidance see CONSORT for harms) | - |
| Discussion | | | |
| Limitations | 20 | Trial limitations, addressing sources of potential bias, imprecision, and, if relevant, multiplicity of analyses | Discussion p18 |
| Generalisability | 21 | Generalisability (external validity, applicability) of the trial findings | Discussion p18 |
| Interpretation | 22 | Interpretation consistent with results, balancing benefits and harms, and considering other relevant evidence | Discussion p18-20 |
| Other information | | |  |
| Registration | 23 | Registration number and name of trial registry | Before the introduction p2 |
| Protocol | 24 | Where the full trial protocol can be accessed, if available | - |
| Funding | 25 | Sources of funding and other support (such as supply of drugs), role of funders | Before the introduction p2 |

*We strongly recommend reading this statement in conjunction with the CONSORT 2010 Explanation and Elaboration for important clarifications on all the items. If relevant, we also recommend reading CONSORT extensions for cluster randomised trials, non-inferiority and equivalence trials, non-pharmacological treatments, herbal interventions, and pragmatic trials. Additional extensions are forthcoming: for those and for up to date references relevant to this checklist, see [www.consort-statement.org](http://www.consort-statement.org/).
