# Supplementary material for: Memantine before Mastectomy Prevents Post-Surgery Pain: A Randomized, Blinded Clinical Trial in Surgical Patients
Source: PLoS One. 2016 Apr 6;11(4):e0152741. doi: 10.1371/journal.pone.0152741 (PMC4822967; doi:10.1371/journal.pone.0152741)
Supplement: S2 File — Prevention of post-mastectomy neuropathic pain with memantine: study protocol for a randomized controlled trial. (PDF) [file pone.0152741.s002.pdf]

Prevention of post-mastectomy neuropathic pain with memantine
A randomized, placebo-controlled, single-blind clinical trial

Short title: Prevention of neuropathic pain by memantine

Version: 3 **Date**: 04/01/2012

**Unique Protocol ID**:	**EudraCT's Number**	**Ethic committee's code**
RBHP 2011 PICKERING 3	2011-004030-33	AU 917

Sponsor :
C.H.U. de Clermont-Ferrand
58 Rue de Montalembert - 63000 Clermont-Ferrand

Principal Investigator:
Dr. Gisèle PICKERING (MCU, PH)
Centre de Pharmacologie Clinique - CIC / Inserm-1405 - Bât 3C
CHU de Clermont-Ferrand, 63000 Clermont-Ferrand

Collaborators:
Dr Xavier DURANDO / Dr Marie-Ange MOURET
Dr Christine VILLATTE / Dr Dominique JOLY
Centre de Lutte contre le Cancer - Jean Perrin
CHU de Clermont-Ferrand, 63000 Clermont-Ferrand

Pr Claude DUBRAY
Centre de Pharmacologie Clinique - CIC / Inserm-1405 - Bât 3C
CHU de Clermont-Ferrand, 63000 Clermont-Ferrand

Biostatistician:
Bruno Pereira, Docteur en Biostatistiques
Direction de la recherche clinique et de l'Innovation
CHU de Clermont-Ferrand, 63000 Clermont-Ferrand

Institutions involved in the trial:
Centre de Lutte contre le Cancer - Jean Perrin
CHU de Clermont-Ferrand, 63000 Clermont-Ferrand

Centre de Pharmacologie Clinique / CIC - Inserm-1405
CHU de Clermont-Ferrand, 63000 Clermont-Ferrand

SUMMARY

TITLE
Prevention of post-mastectomy neuropathic pain with memantine

OBJECTIVE
The primary objective of this study is to evaluate if memantine administered before and after mastectomy may prevent pain development at 3 months post-mastectomy when compared to the placebo group.

TREATMENT
Memantine or placebo (lactose) is given orally for four weeks starting two weeks before surgery. Memantine is given in increasing doses: 5 mg/day for 3 days; 10 mg/day for 3 days; 15 mg/day for 3 days and 20 mg/day for 5 days.
Placebo: Lactose

STUDY DESCRIPTION
A randomized, placebo-controlled, single-blind clinical trial will be performed in the Oncology Hospital, Clermont-Ferrand, France, in 40 women undergoing total mastectomy for breast cancer. Patients will either be allocated to memantine given in increasing doses (maximal dose set at 20mg/day reached to 10th day) or will have placebo (control group).

NUMBER OF PATIENTS
The number of subjects required is 40 chronic pain patients (20 in each group). The minimum δ difference in NS pain between memantine and placebo groups at 3 months is estimated at 1.6 and σ standard deviation at 1.5, estimated from published data of the literature [31, 32], with $\alpha = 0.05$ two-sided situation and $\beta = 0.10$.

ENDPOINTS

The primary endpoint is the pain intensity evaluation by numerical rating scale (NRS) in memantine and placebo groups at 3 months post-mastectomy. The scale ranges from 0 no pain to 10 maximal tolerable pain.

Secondary endpoint
Secondary endpoints are the evaluation of pain at the screening visit, at 2 weeks, 3 months and 6 months after mastectomy (NS, Brief pain Inventory, McGill pain questionnaire), neuropathic pain (Neuropathic Pain in 4 questions, Neuropathic Pain Symptom Inventory), cognition (Trail Making Test, Digit Symbol Substitution Test), sleep (Leeds sleep questionnaire), quality of life (Short-Form-36), anxiety and depression (Hospital Anxiety and Depression scale).

ELIGIBILITY

Inclusion criteria
- Patient aged 18 years and over,
- Patient with a diagnosis of breast cancer, scheduled for mastectomy,
- Patient able to understand and willing to follow the study protocol,
- Patient able to give an informed consent,
- Registration to the French Health Insurance system

Non-inclusion criteria

- Patient having contra-indications to memantine prescription:
  - Hypertension
  - Severe cardiac insufficiency
  - Stroke past-history
- Patient with diabetes (Type I and II),
- Patient treated with specific drugs (amantadine, ketamine, dextromethorphan, L-Dopa, dopaminergic, anticholinergic agonists, barbituric, neuroleptic, IMAO, antispastic agents, dantrolen or baclofen, phenytoin, cimetidine, ranitidine, procainamide, quinidine, quinine, nicotine, hydrochlorothiazide, warfarine),
- Patient in childbearing age with no use of an effective contraceptive method; pregnancy or lactation,
- Patient enrolled in another clinical trial,
- Patient with an inability to comply with the requirements of protocol,
- Patients under tutorship or curatorship,
- Patient not registered to the French Health Insurance system

STUDY DESIGN

Visit 1 – Announcement/Inclusion visit - (D_0-15):

Women with breast cancer are informed by their anesthetist 2 to 3 weeks before mastectomy (D_0-15). During this visit the physician will explain the objectives of the study, the different questionnaires and tests to be performed in order for the patient to give her informed consent to participate to the trial. If need be, additional time will be granted to the patient. Inclusion and exclusion criteria will be thoroughly assessed.

After signature of the written informed consent, a clinical examination will be performed and pain, cognition, quality of life, sleep, anxiety and depression questionnaires will be filled out by the patient.

Participants are randomized in two parallel groups: memantine (n=20) or placebo (n=20). Memantine or placebo (lactose) is given orally for four weeks starting two weeks before surgery. Memantine is given in increasing doses: 5 mg/day for 3 days; 10 mg/day for 3 days; 15 mg/day for 3 days and 20 mg/day for 5 days. Endpoints are reassessed 15 days (D_{+15}), 3 months (D_0+3 months) and 6 months (D_0+6 months) post-mastectomy.

In order to maintain a good compliance and to verify that women do not develop adverse events, patients are called once a week by phone. A booklet for monitoring is completed daily by the patient for 6 months from the day of surgery.

Day -15 to day 0: At home

Memantine will be given in increasing doses during the pre-operative period for 2 weeks. Every 3 days, a phone-call will be done by the study coordinator (Clinical Investigation Center, Pharmacology Department, Clermont-Ferrand, France) in order to collect adverse events.

Visit 2 - Surgery - Day 0

Scheduled mastectomy is performed according to hospital recommendations (Oncology Department, Jean Perrin Center, University hospital, Clermont-Ferrand).

Day 0 to Day 15

Memantine (20mg/day) is administered during 15 post-operative days. Pain will be reported every day on a 0-10 numerical rating scale in collaboration with health professionals according to the post-surgery protocol;: data will be reported daily in the monitoring booklet.
Analgesic concomitant medication and adverse events will be also reported daily in the monitoring booklet.

Visit 3 - Day 16:

The different questionnaires will be filled out (NRS, BPI, Mc McGill pain questionnaire, DN4, NPSI, TMT, DSST, SF-36, HAD and Leeds Sleep questionnaire).

Day 17 to 3 months: At home

Daily average pain assessment over the day with a numerical rating scale, analgesic concomitant medication and possible adverse events will be reported daily by the patient on the monitoring booklet.
From the time the patient is back at home, the study coordinator (Clinical Investigation Center, Pharmacology Department, Clermont-Ferrand, France) will give a weekly phone-call in order to identify possible adverse events. If necessary, the patient will come back to hospital to meet the medical staff.

Visit 4 – Following visit at 3 months

The patient will bring his daily monitoring booklet to the investigator physician in order to observe the evolution of the numerical rating scale, the analgesic concomitant medication and the probable adverse events reported since the day of surgery (D_0).
After clinical examination, the patient will rate his average pain (for 5 days before visit 4) on the numerical rating scale and fill out the following questionnaire and tests:

- Neuropathic pain in 4 questions (DN4),
- Neuropathic Pain Symptom Inventory (NPSI)
- Brief pain inventory (BPI),
- McGill pain questionnaire,
- Short-Form-36 questionnaire (SF-36),
- Leeds Sleep questionnaire,
- Trail Making Test (TMT),
- Digit Symbol Substitution Test (DSST).

A daily monitoring booklet will be given to the patient for the next and last visit at 6 months post-mastectomy.

Visit 5 – Following visit at 6 months

The patient will bring his daily monitoring booklet to the investigator physician in order to observe the evolution of the numerical rating scale, the analgesic concomitant medication and the probable adverse events reported since the day of surgery (D_0).
After a clinical examination, the patient will rate his average pain (for 5 days before visit 5) on the numerical rating scale and fill out the following questionnaire and tests:

- Neuropathic pain in 4 questions (DN4),
- Neuropathic Pain Symptom Inventory (NPSI)
- Brief pain inventory (BPI),
- McGill pain questionnaire,
- Short-Form-36 questionnaire (SF-36),
- Leeds Sleep questionnaire,
- Trail Making Test (TMT),
- Digit Symbol Substitution Test (DSST).

This visit is the visit of the end of the study.

TABLE OF CONTENTS

1. GENERAL INFORMATION 7
1.1. STUDY TITLE 7
1.2. STUDY IDENTIFYING NUMBER AND DATE 7
1.3. SPONSOR 7
1.4. COORDINATING INVESTIGATOR 7
1.5. COLLABORATORS 7
1.6. INSTITUTIONS INVOLVED IN THE TRIAL 7
1.7. DATA ANALYSES 8
BRUNO PEREIRA, DOCTEUR EN BIOSTATISTIQUES 8
1.8. ETHICS COMMITTEE 8
1.9. TIME TABLE 8
2. RATIONAL AND SCIENTIFIC JUSTIFICATION OF THE STUDY 9
2.1. LATEST STATE OF SCIENTIFIC KNOWLEDGE 9
2.2. OBJECTIVES 10
2.3. POTENTIAL BENEFITS AND RISKS, IF ANY, TO HUMAN SUBJECTS 10
2.4. EXPECTED BENEFITS 10
2.5. REFERENCES 11
3. OBJECTIVES OF THE STUDY 13
3.1. MAIN OBJECTIVE 13
3.2. SECONDARY OBJECTIVES 13
4. TRIAL DESIGN 13
4.1. EXPERIMENTAL DESIGN 13
5. DEFINITION OF STUDY POPULATION 13
5.1. INCLUSION CRITERIA 13
5.2. NON-INCLUSION CRITERIA 13
5.3. SUBJECT WITHDRAWAL CRITERIA 14
5.4. REPLACEMENT OF WITHDRAWN SUBJECTS 14
5.5. FOLLOW-UP OF WITHDRAWN SUBJECTS 14
5.6. EXCLUSION PERIOD AND PARTICIPATION TO OTHER CLINICAL TRIAL 15
THE SUBJECT IS NOT ALLOWED TO PARTICIPATE IN ANOTHER CLINICAL TRIAL FOR THE DURATION OF THIS PROTOCOL. IN ADDITION, THE EXCLUSION PERIOD DEFINED IN THIS STUDY IS TWO WEEKS, TIMEFRAME DURING WHICH THE SUBJECT CANNOT PARTICIPATE IN ANOTHER CLINICAL RESEARCH PROTOCOL. 15
5.7. COMPENSATION FOR VOLUNTEERS 15
5.8. RECRUITMENT PROCEDURE 15
6. METHOD 15
6.1. ENDPOINTS 15
6.1.1. PRIMARY ENDPOINT 15
6.1.2. SECONDARY ENDPOINTS 16
6.3. MEASURES TAKEN TO MINIMIZE/AVOID BIASES 23
7. PRACTICAL REALIZATION OF PROTOCOL 23
7.1. EXPECTED DURATION OF PARTICIPATION OF PATIENT AND DESCRIPTION OF THE CHRONOLOGY OF THE TRIAL 23
8. PRODUCT 23
8.1. DESCRIPTION OF TREATMENT 23
8.2. DOSAGE OF ADMINISTRATION AND DURATION OF TREATMENT 24
8.3. PRESENTATION OF PRODUCT 25
ALL PACKAGING, BLISTER PACKS, BAGS, BOXES WILL BE LABELED ACCORDING TO THE RULES RELATED TO THE BIOMEDICAL RESEARCH. 26
8.4. COMPLIANCE 26

8.5. DRUGS PERMITTED AND PROHIBITED DURING THE TRIAL....27
9. COLLECTED DATA....28
10. STATISTICAL CONSIDERATIONS....28
10.1. NUMBER OF PATIENTS TO BE INCLUDEDNOMBRE DE SUJETS A INCLURE....28
10.2. DATA ANALYSES....28
11. ASSESSMENT OF SAFETY....29
11.1 DEFINITIONS....29
11.2. ADVERSE EVENTS AND REPORTED PROCEDURES....30
11.4. TYPE AND DURATION OF THE FOLLOW-UP OF SUBJECTS AFTER ADVERSE EVENTS....32
12. DIRECT ACCESS TO SOURCE DATA DOCUMENT....32
13. QUALITY CONTROL AND QUALITY ASSURANCE....34
14. ETHICS CONSIDERATIONS....35
15. DATA HANDLING AND RECORD KEEPING....36
16. FINANCING AND INSURANCE....37
17. COMMUNICATION – PUBLICATION RULES....37
18. FEASABILITY OF THE STUDY....37
19. LIST OF ANNEXES....37

1. GENERAL INFORMATION

1.1. Study title

Prevention of post-mastectomy neuropathic pain with memantine

1.2. Study identifying number and date

Sponsor Code : RBHP 2011 PICKERING 3
EUDRACT number: 2011-004030-33

1.3. Sponsor

CHU de Clermont-Ferrand
58 rue Montalembert
63000 Clermont-Ferrand
Direction Générale Adjointe – Délégation Régionale à la Recherche Clinique
Tél : 04.73.751.195 / Fax : 04.73.754.730

1.4. Coordinating investigator

Dr. Gisèle PICKERING (MCU, PH)
Centre de Pharmacologie Clinique / Centre d'Investigation Clinique,
CHU de Clermont-Ferrand, 63000 Clermont-Ferrand

1.5. Collaborators

Dr Xavier DURANDO
Dr Marie-Ange MOURET
Dr Christine VILLATTE
Dr Dominique JOLY
Centre de lutte contre le Cancer - Jean Perrin
CHU de Clermont-Ferrand, 63000 Clermont-Ferrand

Pr Claude DUBRAY
Centre de Pharmacologie Clinique / Centre d'Investigation Clinique - Inserm 1405
CHU de Clermont-Ferrand, 63000 Clermont-Ferrand

1.6. Institutions involved in the trial

Centre de lutte contre le Cancer - Jean Perrin - CHU de Clermont-Ferrand, 63000 Clermont-Ferrand
Centre de Pharmacologie Clinique / CIC - Inserm 1405 - CHU de Clermont-Ferrand

1.7. Data analyses

Bruno PEREIRA, Docteur en Biostatistiques (PhD)
Direction de la Recherche Clinique et de l'Innovation
CHU de Clermont-Ferrand, 63000 Clermont-Ferrand

1.8. Ethics committee

Ethics committee (CPP Sud Est VI, France)

1.9. Time table

Date of submission to the Ethics Committee: 2ème semestre 2011
Date of the estimated to the Ethics Committee approval: 2ème semestre 2011
Date of submission to ANSM (French Drug Administration): 1^{èr} semestre 2012 – 1er semestre 2013
Date of the estimated to ANSM approval: 2ème semestre 2013
Date of the conduct of the study: 2ème semestre 2013

2. RATIONAL AND SCIENTIFIC JUSTIFICATION OF THE STUDY

2.1. Latest state of scientific knowledge

Medical treatment of neuropathic pain (NP) is still far from being satisfactory, with less than half the patients achieving significant benefit with any pharmacological drug (Finnerup et al., 2005). Several therapies have been developed for the treatment of NP but these methods are not equally effective for all NP patients. N-Methyl-D-Aspartate receptor (NMDAR) antagonists like ketamine, memantine or dextromethorphan are potential drugs for NP alleviation (Collins et al., 2010). Evidence suggests that NMDAR within the dorsal horn plays an important role in both inflammation and nerve injury-induced central sensitization (Bleakman et al., 2006). Activation of NMDAR is associated with abnormalities in the sensory (peripheral and central) system, resulting in neuronal excitation and abnormal pain manifestations (spontaneous pain, allodynia, hyperalgesia) (Petrenko et al., 2003). Blocking these receptors by antagonists leads to a reduction of pain (Zhou et al., 2011). A recent review of the literature including 28 randomized clinical trials (Collins et al., 2010) emphasizes the heterogeneity of doses used, the diversity of pathologies generating neuropathic pain (post-herpetic, post-amputation, diabetes...) and highlights the need to develop clinical trials of good methodological quality with NMDA antagonists. NMDAR antagonists, such as ketamine (Sang et al., 2002; Corell et al., 2004) are prescribed after therapeutic failure with classical treatment but these drugs have severe adverse events that limit their clinical use (Cvrcek et al., 2008). Another NMDA antagonist, memantine, prescribed in Alzheimer's disease to maintain cognitive function, has minimal side-effects at doses within the therapeutic range, probably because of its specific mechanism of action as it is an uncompetitive antagonist with moderate affinity, strong voltage-dependency and rapid unblocking kinetics (Parsons et al., 1999a; 1999b; Jonhson et al., 2006). Concerning NP alleviation, memantine shows controversial results in human studies (Sang et al., 2002; Eisenberg et al., 1998; Hackworth et al., 2008).

We recently demonstrated for the first time in an animal surgical NP model, that memantine prevents the development of NP symptoms and the impairment of spatial memory (Morel et al., 2013). With a translational approach, we present a clinical study where memantine (*vs* placebo) is administered 2 weeks before and 2 weeks after mastectomy in 40 women suffering from breast cancer. Confirmation of preclinical results in this clinical study would constitute a major step for NP prevention by memantine and maintenance of cognition and quality of life in these vulnerable patients.

2.2. Objectives

Main objective

To evaluate if memantine administered for four weeks starting two weeks before mastectomy may prevent post-surgery pain at 3 months post-surgery compared to the placebo group

Secondary objectives

To estimate at 3 and 6 months post-mastectomy the pain intensity, the analgesic concomitant medications, the impact of treatment (memantine/placebo) on cognitive function, quality of life, sleep, anxiety and depression.

2.3. Potential benefits and risks, if any, to human subjects

Benefits

Patients followed in this protocol will, if the results obtained in animals are confirmed to have an improvement in their pain induced by surgery, with potentially an overall improvement in their quality of life.

Risks

Memantine is prescribed in Alzheimer's disease and the chosen dose for this pathology is set at 20mg/day (according to the French Drug Agency Regulations, AMM). The expected side-effects of this molecule are dizziness, headache, constipation, somnolence and hypertension, and are described in the information consent. The intensity of these effects is low to moderate.

To prevent the occurrence of these adverse effects, it is recommended to titrate: 5 mg/day for 7 days; 10 mg/day for 7 days; 15 mg/day for 7 days and 20 mg/day for 7 days.

In this trial, the dose escalation levels are reduced to 3 days in order to respect the surgical management of patients (surgery are usually scheduled two weeks after the anesthetic visit. In this context, a phone call will be performed every three days (at the end of each level) in order to collect adverse events.

2.4. Expected benefits

This study will evaluate at 3 months and 6 months post-mastectomy if:

1. memantine may be prescribed preventively for neuropathic pain, when drugs prescribed today are very variable: type of drug, dosage and duration of administration,
2. it is possible to consider the extension of this prophylaxis to other surgery- induced neuropathic pain.

2.5. References

1. Finnerup NB, Otto M, McQuay HJ, Jensen TS, Sindrup SH. Algorythm for neuropathic pain treatment: an evidence based proposal. Pain 2005, 118(3):239-305.
2. Collins S, Sigtermans MJ, Dahan A, Zuurmond WW, Perez RS. NMDA receptor antagonists for the treatment of neuropathic pain. Pain Med. 2010, 11(11):1726-42.
3. Bleakman D, Alt A, Nisenbaum ES. Glutamate receptors and pain. Semin Cell Dev Biol. 2006, 17(5):592-604.
4. Petrenko AB, Yamakura T, Baba H, Shimoji K. The role of N-methyl-D-aspartate (NMDA) receptors in pain: a review. Anesth Analg. 2003, 97(4):1108-16.
5. Zhou HY, Chen SR, Pan HL. Targeting N-methyl-D-aspartate receptors for treatment of neuropathic pain. Expert Rev Clin Pharmacol. 2011, 4(3):379-88.
6. Sang CN, Booher S, Gilron I, Parada S, Max, MB. Dextromethorphan and memantine in painful diabetic neuropathy and postherpetic neuralgia: efficacy and dose-response trials. Anesthesiology 2002, 96(5):1053-1061.
7. Correll GE, Maleki J, Gracely EJ, Muir JJ, Harbut RE. Subanesthetic ketamine infusion therapy: a retrospective analysis of a novel therapeutic approach to complex regional pain syndrome. Pain Med. 2004, 5(3):263-275.
8. Cvrcek P. Side effects of ketamine in the long-term treatment of neuropathic pain. Pain Med. 2008, 9:253-257.
9. Parsons CG, Danysz W, Bartmann A, Spielmanns P, Frankiewicz T, Hesselink M, Eilbacher B, Quack G. Amino-alkyl-cyclohexanes are novel uncompetitive NMDA receptor antagonists with strong voltage-dependency and fast blocking kinetics: in vitro and in vivo characterization. Neuropharmacology 1999a, 38:85-108.
10. Parsons CG, Danysz W, Quack G. Memantine is a clinically well tolerated NMDA receptor antagonist-a review of preclinical data. Neuropharmacology 1999b, 38:735-767.

11. Jonhson JW, Kotermanski SE. Mechanism of action of memantine. Curr. Opin. Pharmacol. 2006, 6:61-67.

12. Eisenberg E, Kleiser A, Dortort A, Haim T, Yarnitsky D. The NMDA (N-methyl-D-aspartate) receptor antagonist memantine in the treatment of postherpetic neuralgia: a double-blind, placebo-controlled study. Eur. J. Pain 1998, 2:321-327.

13. Nikolajsen L, Gottrup H, Anders GD, Jensen TS. Memantine (a N-methyl-D-aspartate receptor antagonist) in the treatment of neuropathic pain after amputation or surgery: A randomized, double-blind, cross-over study. Anesth. Analg. 2000, 91:960-966.

14. Maier C, Dertwinkel R, Mansourian N, Hosbach I, Schwenkreis P, Senne I, Skipka G, Zenz M, Tegenthoff M. Efficacy of the NMDA-receptor antagonist memantine in patients with chronic phantom limb pain-results of a randomized double-blinded, placebo-controlled trial. Pain 2003, 103:277-283.

15. Hackworth RJ, Tokarz KA, Fowler IM, Wallace SC, Stedje-Larsen ET. Profound pain reduction after induction of memantine treatment in two patients with severe phantom limb pain. Anesth. Analg. 2008, 107:1377-1379.

16. Morel V, Etienne M, Wattiez AS, Dupuis A, Privat AM, Chalus M, Eschalier A, Daulhac L, and Pickering G. Memantine, a promising drug for the prevention of neuropathic pain in rat. Eur. J. Pharmacol. 2013, 721:382-390.

3. OBJECTIVES OF THE STUDY

3.1. Main objective

The primary objective of this study is to evaluate if memantine administered before and after mastectomy may prevent pain development at 3 months post-mastectomy when compared to the placebo group.

3.2. Secondary objectives

The secondary objectives are to estimate at 3 and 6 months post-mastectomy the pain intensity, the analgesic concomitant medications, the impact of treatment (memantine/placebo) on cognitive function, quality of life, sleep, anxiety and depression, the impact of cancer chemotherapy-induced pain and cognitive impairment.

4. TRIAL DESIGN

4.1. Experimental design

A randomized, placebo-controlled, single-blind clinical trial in the Oncology Hospital, Clermont-Ferrand, France, in 40 women undergoing total mastectomy for breast cancer. Patients will either be allocated to memantine given in increasing doses (maximal dose set at 20mg/day reached to 10th day) or will receive placebo (control group).

5. DEFINITION OF STUDY POPULATION

5.1. Inclusion criteria

- Patient aged 18 years and over,
- Patient with a diagnosis of breast cancer, scheduled for mastectomy,
- Patient able to understand and willing to follow the study protocol,
- Patient able to given an informed consent,
- Registration to the French Health Insurance system

5.2. Non-inclusion criteria

- Patient having contra-indications to memantine prescription:
  - Hypertension
  - Severe cardiac insufficiency

  - Stroke past history
- Patient with diabetes (Type I and II),
- Patient treated with specific drugs (amantadine, ketamine, dextromethorphan, L-Dopa, dopaminergic, anticholinergic agonists, barbituric, neuroleptic, IMAO, antispastic agents, dantrolen or baclofen, phenytoin, cimetidine, ranitidine, procainamide, quinidine, quinine, nicotine, hydrochlorothiazide, warfarine),
- Patient in childbearing age with no use of an effective contraceptive method; pregnancy or lactation,
- Patient enrolled in another clinical trial,
- Patient with an inability to comply with the requirements of protocol,
- Patients under tutorship or curatorship,
- Patient not registered to the French Health Insurance system.

5.3. Subject withdrawal criteria

Exclusion criteria during the trial:

- Major intolerance with the tested product,
- Occurrence of adverse events requiring to stop taking part to the trial estimated as such by the investigator.

Subjects may discontinue their participation in the study at any time.
The clinical Investigator may ask a subject to withdraw, if, in his opinion, it is in the best interest of the subject.
A subject may be withdrawn from the study for any of the following reasons:

- Withdrawal of consent - any subject may withdraw from the study at any time
- Significant deviation from the protocol
- Incidental illness
- Occurrence of a side adverse event

5.4. Replacement of withdrawn subjects

In case of withdrawal of a subject for any reason, the subject will be replaced, except if the reason is the occurrence of an adverse event.

5.5. Follow-up of withdrawn subjects

In case of withdrawal of a subject due to an adverse event, the follow-up of the subject will continue until the adverse event (or the sequels to the adverse event) is completely resolved.

5.6. Exclusion period and participation to other clinical trial

The subject is not allowed to participate in another clinical trial for the duration of this protocol. In addition, the exclusion period defined in this study is two weeks, timeframe during which the subject cannot participate in another clinical research protocol.

5.7. Compensation for volunteers

The subjects will receive 200€ of compensation for the entire participation.
In the case of a premature withdrawal, a calculation of the compensation will be made in proportion to protocol completion, as follows:

- withdraw after the first period of treatment (V2): 50€
- withdraw after the visit 3 (D_0+15 days): 100€
- withdraw after the visit 4 (D_0+3 months): 150€

5.8. Recruitment procedure

Women with breast cancer are informed by their anesthetist 2 to 3 weeks before mastectomy (D_0-15) in the Oncology Department (Jean Perrin). During this visit the physician will explain the objectives of the study, the different questionnaires and tests to be performed in order for the patient to give her informed consent to participate to the trial. If needed be, additional will be granted to the patient. Inclusion and exclusion criteria will be also verified.

After signature of the written informed consent, a clinical examination will be performed and pain, cognition, quality of life, sleep, anxiety and depression questionnaires will be filled out by the patient.
In order to maintain a good compliance and to verify that women do not develop adverse events, patients will be called once a week by phone. A booklet for monitoring will be completed daily by the patient for 6 months starting from the day of surgery (D_0).

6. METHOD

6.1. Endpoints

6.1.1. Primary endpoint

- **<u>Numerical rating scale:</u>**

The primary endpoint is the pain intensity evaluation by numerical scale (NS) in memantine and placebo groups at 3 months post-mastectomy. The scale ranges from 0 no pain to 10 maximal tolerable pain.

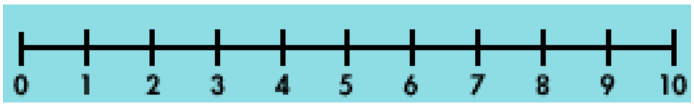

6.1.2. Secondary endpoints

Secondary endpoints are the evaluation of pain at the screening visit, at 2 weeks, 3 months and 6 months after mastectomy (NRS, Brief pain Inventory, McGill pain questionnaire), neuropathic pain (Neuropathic Pain in 4 questions, Neuropathic Pain Symptom Inventory), cognition (Trail Making Test, Digit Symbol Substitution Test), sleep (Leeds sleep questionnaire), quality of life (Short-Form-36), anxiety and depression (Hospital Anxiety and Depression scale).

***Pain assessment*:**

- **Numerical Rating Scale (NRS)**: The scale ranges from 0 no pain to 10 maximal tolerable pain.

- **The Brief Pain Inventory (BPI) questionnaire:** This self-administered questionnaire provides information on the intensity of pain, along with the degree to which the pain interferes with the everyday functioning of life including: mood, walking, general activity, relations with others, sleep, enjoyment of life.

- **The Mc McGill pain questionnaire:** This questionnaire allows to describe pain experienced during the last 48 hours. It has fifty eight qualifiers divided into sixteen items (A to P) . Each qualifier is rated from 0 to 4, where 0 = absent, 1 = low, 2 = moderate, 3 = strong, 4 = very strong. The score is divided between two subclasses, sensory subclass (items A to I) and emotional subclass (items J to P).

- **The Neuropathic pain assessment with Neuropathic pain in 4 questions (DN4):** DN4 is a clinical tool for the qualification of neuropathic pain. This questionnaire has four questions divided into 10 items related to the interview (ie, symptoms) and to the sensory examination (ie, signs). The investigator asks and examines the patient and notes a response "no" or "yes" for each item: "yes" is scored as "1" and "no" is scored as "0". The sum of scores gives the total score of the patient (/10). DN4 is considered as positive if the patient obtains a score of 4/10.

- **The Neuropathic pain Symptom Inventory (NPSI):** NPSI is a self-questionnaire and includes 10 pain descriptors. Intensity is rated on 0-10 numerical scales and two temporal items are designed to assess spontaneous ongoing pain duration and the number of pain paroxysms over 24h. This questionnaire discriminates 5 distinct clinically relevant dimensions: spontaneous burning pain, spontaneous deep pain, paroxysmal pain, evoked pain, and paresthesia/dysesthesia.

- **The analgesic concomitant medication** will be classified according to the pain treatment (Step 1: paracetamol, non-steroidal anti-inflammatory drugs (NSAIDs), Step 2: tramadol, codeine, lamaline, Step 3: morphine and opiates) and neuropathic pain treatments (antidepressants, antiepileptics, lidocaine plaster). Concerning opioid consumption, opioids taken for postoperative pain from will be differentiated from those prescribed for neuropathic pain. The analgesic consumption will be evaluated during 3 periods: between 1) the day of surgery and 15 days post-mastectomy (D_0 to D_0+15 days), 2) 15 days and 3 months post-surgery (D_0+15 to D_0+3 months) and 3) 3 months and 6 months post-mastectomy (D_0+3 months to D_0+6 months). Conversion of opioid doses will be done in order to harmonize the data according to international recommendations.

<u>Cognition assessment</u>:

- **The Trail Making Test (TMT):** This non-verbal cognitive test assesses the ability of speed, executive functions, attention, concentration, visual perceptual speed. The test takes place in two parts. In Part A, circles are numbered from 1 to 25 and the patient must connect with lines the numbers in ascending order (1-2-3-4, etc.). In Part B, the circles contain numbers from 1 to 13 and letters from A to L, the patient must connect the circles with lines but alternating numbers and letters (1A-2B -3C, etc.). The patient must connect the circles as quickly as possible for both parts of the test, without lifting the pen from the paper. The TMT B additionally provides an estimate of mental flexibility.

- **The Digit Symbol Substitution Test (DSST):** DSST is a neuropsychological, nonverbal test, which assesses cognitive deficit and brain damage associated with aging and/or depression. It also evaluates learning ability, concentration and attention. It consists in combining pairs of symbols and numbers as quickly as possible and the score is the correct number of symbols in the time allowed (e.g. 90 or 120 seconds).

<u>Quality of life assessment</u>:

- **The Short-Form-36 questionnaire (SF-36):** The SF-36 is a questionnaire evaluating the quality of life of patients. It is a multidimensional scale that assesses the health and quality of life. This scale can be performed in self-or hetero-questionnaire with 36 items including nine dimensions: physical function (PF), role physical (RP), bodily pain (BP), general health (GH), vitality (VT), social functioning (SF), role emotional (RE), mental health (MH) and health thinking (HT).

Sleep assessment:

- **The Leeds sleep evaluation questionnaire** is a standardized self-administered questionnaire composed of ten visual analogue scales that relate to four aspects of sleep efficiency:

  - Quality of sleep, getting to sleep (visual scales 1, 2 and 3),
  - Sleep quality (visual scales 4 and 5),
  - awakening from sleep (visual scales 6, 7 and 8),
  - behavior following wakefulness (visual scales 9 and 10).

6.2. Description of the research methodology

A randomized, placebo-controlled, single-blind clinical trial in patients diagnosed with breast cancer who require total mastectomy, will either be allocated to memantine given in increasing doses (maximal dose set at 20mg/day reached to 10th day) or will have placebo (control group).

STUDY DESIGN

Visit 1 – Announcement/Inclusion visit - (D_0-15):

Women with breast cancer are informed by their anesthetist 2 to 3 weeks before mastectomy (D_0-15). During this visit the physician will explain the objectives of the study, the different questionnaires and tests to be performed in order for the patient to give her informed consent to participate to the trial. If need be, additional time will be granted to the patient. Inclusion and exclusion criteria will be thoroughly assessed.

After signature of the written informed consent, a clinical examination will be performed and pain, cognition, quality of life, sleep, anxiety and depression questionnaires will be filled out by the patient.

Participants are randomized in two parallel groups: memantine (n=20) or placebo (n=20). Memantine or placebo (lactose) is given orally for four weeks starting two weeks before surgery. Memantine is given in increasing doses: 5 mg/day for 3 days; 10 mg/day for 3 days; 15 mg/day for 3 days and 20 mg/day for 5 days. Endpoints are reassessed 15 days (D_{+15}), 3 months (D_0+3 months) and 6 months (D_0+6 months) post-mastectomy.

In order to maintain a good compliance and to verify that women do not develop adverse events, patients are called once a week by phone. A booklet for monitoring is completed daily by the patient for 6 months from the day of surgery.

Day -15 to day 0: At home

Memantine will be given in increasing doses during the pre-operative period for 2 weeks. Every 3 days, a phone-call will be done by the study coordinator (Clinical Investigation Center, Pharmacology Department, Clermont-Ferrand, France) in order to collect adverse events.

Visit 2 - Surgery - Day 0

Scheduled mastectomy is performed according to hospital recommendations (Oncology Department, Jean Perrin Center, University hospital, Clermont-Ferrand).

Day 0 to Day 15

Memantine (20mg/day) is administered during 15 post-operative days. Pain assessment will be reported every day on a 0-10 numerical rating scale in collaboration with health professionals according to the post-surgery protocol; data will be reported daily in the monitoring booklet.
Analgesic concomitant medication and adverse events will be also reported daily in the monitoring booklet.

Visit 3 - Day 16:

The different questionnaires will be filled out (NRS, BPI, Mc McGill pain questionnaire, DN4, NPSI, TMT, DSST, SF-36 and Leeds Sleep questionnaire).

Day 17 to 3 months: At home

Daily average pain assessment over the day with a numerical rating scale, analgesic concomitant medication and possible adverse events will be reported daily by the patient on the monitoring booklet.
From the time the patient is back at home, the study coordinator (Clinical Investigation Center, Pharmacology Department, Clermont-Ferrand, France) will give a weekly phone-call in order to identify possible adverse events. If necessary, the patient will come back to hospital to meet the medical staff.

Visit 4 – Follow-up visit at 3 months

The patient will bring his daily monitoring booklet to the investigator physician in order to observe the evolution of the numerical rating scale, the analgesic concomitant medication and the probable adverse events reported since the day of surgery (D_0).
After clinical examination, the patient will rate his average pain (for 5 days before visit 4) on the numerical rating scale and fill out the following questionnaire and tests:

- Neuropathic pain in 4 questions (DN4),
- Neuropathic Pain Symptom Inventory (NPSI)
- Brief pain inventory (BPI),
- McGill pain questionnaire,
- Short-Form-36 questionnaire (SF-36),
- Leeds Sleep questionnaire,
- Trail Making Test (TMT),
- Digit Symbol Substitution Test (DSST).

A daily monitoring booklet will be given to the patient for the next and last visit at 6 months post-mastectomy.

Visit 5 – Follow-up visit at 6 months

The patient will bring his daily monitoring booklet to the investigator physician in order to observe the evolution of the numerical rating scale, the analgesic concomitant medication and the probable adverse events reported since the day of surgery (D_0).
After a clinical examination, the patient will rate his average pain (for 5 days before visit 5) on the numerical rating scale and fill out the following questionnaire and tests:

- Neuropathic pain in 4 questions (DN4),
- Neuropathic Pain Symptom Inventory (NPSI)
- Brief pain inventory (BPI),
- McGill pain questionnaire,

- Short-Form-36 questionnaire (SF-36),
- Leeds Sleep questionnaire,
- Trail Making Test (TMT),
- Digit Symbol Substitution Test (DSST).

This visit is the visit of end of study.

Study design:

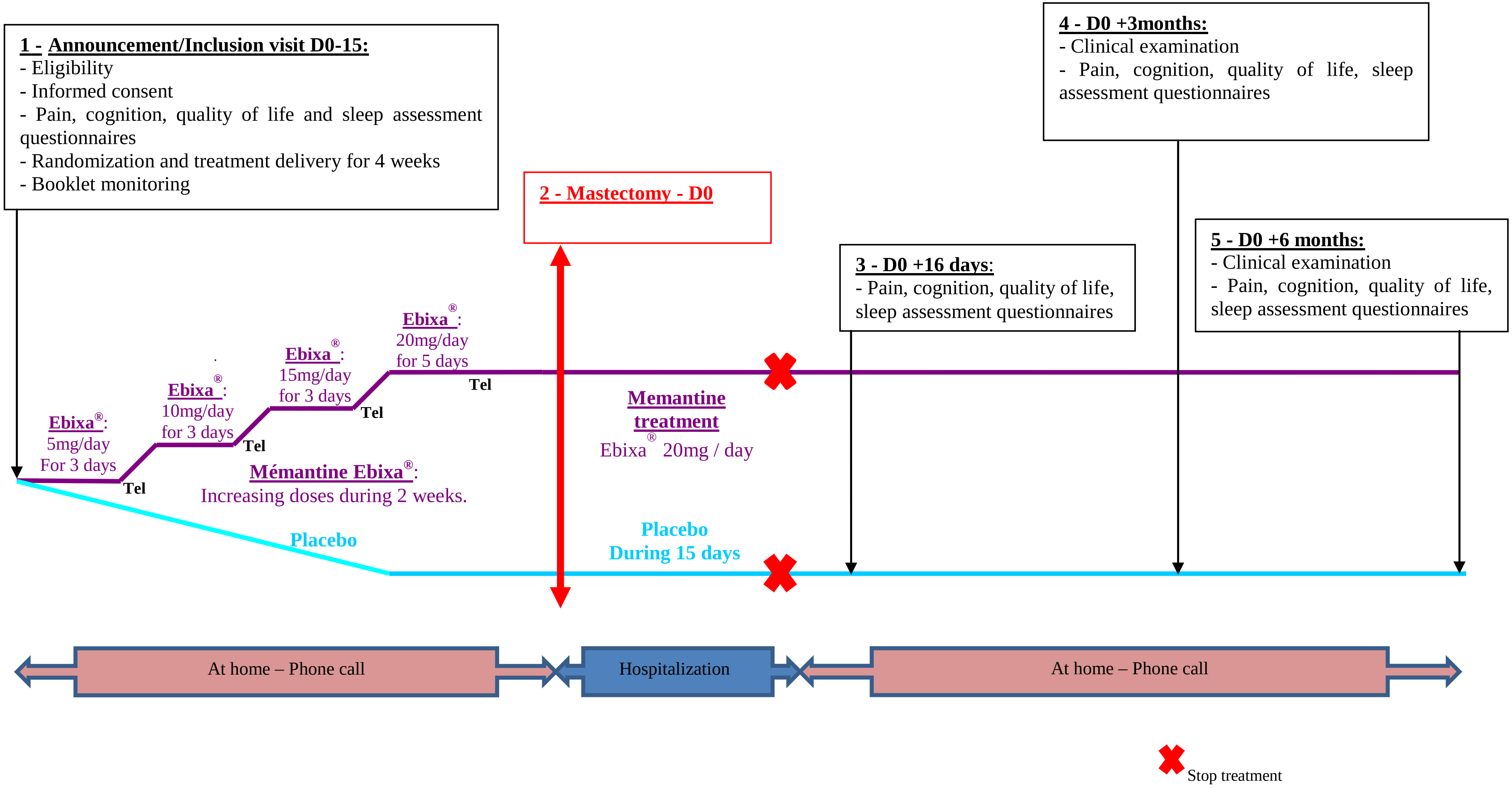

Summary of assessment for one patient:

Visits	Annoucement	Surgery	Hospitalization	Follow-up	Follow-up	Follow-up
Days of visit	D0-15	D0	D0 to D0+15	D0+16	D0 +3months	D0 +6months
Center	CJP	CJP	CJP	CJP or CIC	CIC	CIC
Informed consent	+					
Checking inclusion and non-inclusion criteria	+					
Clinical examination	+	+			+	+
Mastectomy		+				
Hospitalization			+			
Filling out questionnaires:						
- Leeds, SF36, TMT A and B, DSST	+					
- DN4, NPSI, TMT A and B, DSST				+		
- DN4, QCD, QDSA, Leeds, SF36, NPSI, TMT A and B, DSST					+	+
Treatment pre and post-surgery	+					
Phone-call – adverse event collection	+	+		+	+	+

.

6.3. Measures taken to minimize/avoid biases

Patients will be randomized in two groups as follow:

- Memantine during 28 days
- Placebo during 28 days

Treatment allocation follows a predetermined randomization list and is carried out by a person totally independent from the protocol. The randomization sequence is generated using random blocks.

7. PRACTICAL REALIZATION OF PROTOCOL

7.1. Expected duration of participation of patient and description of the chronology of the trial

The duration of the treatment will be 28 days. The total and maximal duration of participation per patient will be 7 months.

The protocol will include 5 visits (D0-15, D0, D0+15, D0+3months and D0+6months) and 1 hospitalization post-operative-period per patient.

The total duration of the study is estimated at 2 years.

The date of the end of the study will be forwarded to the competent authority and the CPP within 90 days. In the event of premature withdrawal, the information will be sent within 15 days to the competent authority and the CPP.

8. PRODUCT

8.1. Description of treatment

Memantine: Memantine has been used since the seventies. Its structure is similar to that of amantadine or Mantadix®, that is used as anti-Parkinson and against influenza.

Pharmacologically, memantine was first considered as a muscle relaxant and the non-competitive antagonist of NMDA glutamate receptors. Memantine has been experimentally and clinically tested in a variety of neurological disorders including Parkinson's disease. Its activity in Alzheimer's disease has been demonstrated in a controlled study in 1991, the results were confirmed and clarified by several other studies. Memantine is in addition to anticholinesterase as therapeutic possibility of Alzheimer's disease.

8.2. Dosage of administration and duration of treatment

Memantine group: product used: Ebixa® 10 mg and 20 mg - tablets

It is given in increasing doses for the pre-operative period: 5 mg/day for 3 days, 10mg/day for 3 days, 15mg/day for 3 days, 20mg/day for 5 days to the post-operative period (D_0+15).

Step1: Ebixa® 5 mg/day for 3 days: 1/2 tablet of Ebixa® 10 mg, taken in the morning for 3 days

Step 2: Ebixa® 10 mg/day for 3 days: 1 tablet of Ebixa® 10 mg, taken at the morning for 3 days

Step 3: Ebixa® 15 mg/day for 3 days: 1.5 tablet of Ebixa® 10 mg taken at the morning for 3 days

Step 4: Ebixa® 20 mg/day for 5 days: 1 tablet of Ebixa® 20 mg taken at the morning for 5 days

Post-operative period : Ebixa® 20 mg/day for 15 days : 1 tablet of Ebixa® 20 mg taken at the morning to 15 days after surgery (D_0+15).

Placebo : lactose

Summary memantine prescription

	Morning
Step 1	
Ebixa® 5 mg	1/2 tb Ebixa® 10 mg
Step 2	
Ebixa® 10 mg	1 tb Ebixa® 10 mg
step 3	
Ebixa® 15 mg	1,5 tb Ebixa® 10 mg
Step 4 (post-operative period)	
Ebixa® 20 mg	1 tb Ebixa® 20 mg

Placebo:

During the entire study (28 days), one placebo capsule (lactose powder) taken in the morning.

Summary Placebo prescription:

	Morning
Pre- and post-operative period	
Powder of Lactose	1 capsule of placebo

8.3. Presentation of the drugs

Memantine:

The Ebixa® tablets remain in their original blister, the amount required for each session is packed in a bag with appropriate labeling. The step bags 1, 2, 3, 4 and postoperative weeks will be put in a cardboard box for home use by the patient during the two weeks before surgery and for the postoperatively period, during hospitalization.

Placebo:

Capsules: Size 0 and color (blue) will be filled with lactose powder. The capsule will then be prepared in blisters; the necessary quantity of placebo capsules for each period will be packed in a bag labeled with the corresponding period. The bags of preoperative and postoperative periods will be put in a cardboard box for the entire duration of the study.

Presentation of product :

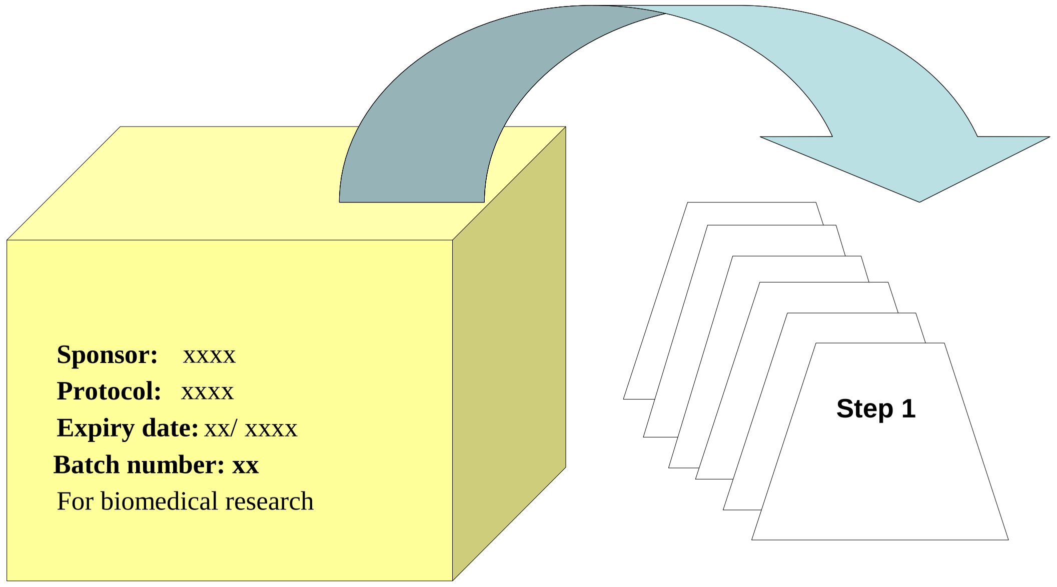

All Packaging, blister packs, bags, boxes will be labeled according to the rules related to the Biomedical Research.

Presentation of labels:

Subject code: _ _ _ / _ _ / _ _ _ / _ _
Sponsor : CHU de Clermont-Ferrand
Protocol : xxxxxxxxxx
Eudract number : xxxxxxxxx

DOSE - EBIXA®
Step1: Ebixa® 5 mg/day for 3 days : 1/2 tablet of Ebixa® 10 mg, taken in the morning for 3 days
Step 2: Ebixa® 10 mg/day for 3 days: 1 tablet of Ebixa® 10 mg, taken at the morning for 3 days
Step 3: Ebixa® 15 mg/day for 3 days: 1.5 tablet of Ebixa® 10 mg taken at the morning for 3 days
Step 4: Ebixa® 20 mg/day for 5 days: 1 tablet of Ebixa® 20 mg taken at the morning for 5 days
Post-operative period : Ebixa® 20 mg/day for 15 days : 1 tablet of Ebixa® 20 mg taken at the morning to 15 days after surgery (D_0+15).

8.4. Compliance

The drugs will be provided by the central pharmacy of Clermont-Ferrand Hospital and stored for the duration of the study in the unit involved in the trial. Any unused product and empty blisters will be accounted at the end of the study and returned to the central pharmacy of Clermont-Ferrand Hospital.

Empty blisters will be collected by the clinical research associate in order to account them and to evaluate the treatment administration.

8.5. Drugs permitted and prohibited during the trial

Permitted treatment during the trial:
All long-term treatment of the disease is allowed, no therapeutic changes will be generated by the protocol, patients will be treated with memantine or usual care.

Prohibited treatment during the trial:

Contra-indication associated with

- Non-selective IMAO, selective IMAO, linézolide,
- Amantadine,
- Ketamine, dextrometorphan,
- L-Dopa, agonistes dopaminergiques, anticholinergiques,
- Barbiturics (phénobarbital, butobarbital, amylobarbital, quinalbarbital, pentobarbital, hexobarbital, thiopental, méthoxytal),
- Neuroleptics (phénothiazines (chlorpromazine, fluphénazine, perphénazine, prochlorpérazine), thioridazine, trifluopérazine, halopéridol + décanoate (action prolongée), pimozide, cyamémazine), clozapine, olanzapine, rispéridone, quétiapine, ziprasidone, aripiprazole,
- antispastic agents, dantrolen or baclofen
- Phenytoin,
- Cimétidine,
- Ranitidine,
- Procaïnamide,
- Quinidine, quinine, nicotine,
- Hydrochlorothiazide,
- Warfarine.

This list of unauthorized drugs during the trial will be given to the subject.

9. COLLECTED DATA

All data of the interrogation, clinical examination, questionnaires and tests of cognitive parameters will be reported in the medical record that will constitute the data sources of the protocol. These data should be reported in a specific CRF protocol or as anonymised copies attached to the case report form for the questionnaires.

The data will be entered by the Clinical research associate in computer files using a double entry procedure meeting the standards set by good clinical practice. After comparison of double entry, data computer files and any corrections to be made to them will be retained and retrievable on demand.

10. STATISTICAL CONSIDERATIONS

10.1. Number of patients to be included

The number of subjects required is 40 chronic pain patients (20 in each group). The minimum δ difference in NS pain between memantine and placebo groups at 3 months is estimated at 1.6 and σ standard deviation at 1.5, with $\alpha = 0.05$ two-sided situation and $\beta = 0.10$.

10.2. Data analyses

Statistical analyses will be performed with Stata software (version 13; StataCorp, College Station, US). Concerning the primary objective, comparison between the randomized groups will be performed using the Student test or the Mann and Whitney test (if the conditions for validity of the Student test are not respected, normality verified by Shapiro-Wilk and homoscedasticity by Fisher-Snedecor test). Then, to study the evolution of several parameters and to compare randomization groups, data analyses will be performed using mixed models which allow to consider, on the one hand, time, group and interaction time x group as fixed effects and on the other hand, the within and between subject variability. Residual normality will be checked for all considered models. When appropriate, anticancer chemotherapy (yes/no) will be studied as fixed effect in these models before considering sub-groups analyses. The comparison between the treatment groups will be performed systematically: (1) without adjustment and, (2) by adjusting other factors whose repartition could be, despite the

randomization, unbalanced between the treatment groups. The tests will be two-sided, with a type I error set at $\alpha=0.05$. A sensitivity analysis of missing data will be performed, to ensure the pertinence of the longitudinal data (MAR or MCAR).

10.3. Method for missing, unused or invalid data

In case of premature discontinuation, the patient will be replaced with the same treatment allocation according to an allocation of alternative batches predetermined in the randomization plan.
No replacement of missing data. Indeed, the mixed model approach is used to manage unbalanced protocols and / or reduced and to jointly study the evolution of all patients even if some have few measures. A sensitivity analysis for missing data will be made in particular to ensure the relevance of longitudinal data (MAR or MCAR).

11. ASSESSMENT OF SAFETY

11.1 Definitions

The following definitions are to be applied, in accordance with ICH[1] guidelines on the reporting of safety data.

• **Adverse Event/Experience (AE):**	Any untoward medical occurrence in a patient or clinical investigation patient administered a pharmaceutical product and which does not necessarily have to have a causal relationship with this treatment.<br>An AE can therefore be any unfavourable and unintended sign (including an abnormal laboratory finding) symptom or disease temporally associated with the use of a medicinal product, whether or not considered related to the medicinal product.

[1] ICH Harmonised Tripartite Guideline E2A: Note for Guidance on Clinical Safety Data Management - Definitions and Standards for Expedited Reporting (CPMP/ICH/337/95) Step 5, adopted by CPMP November 1994.

• **Serious Adverse Event/Reaction (SAE):**	Any untoward medical occurrence that at any dose:<br>• results in death;<br>• is life-threatening;<br>• requires in-patient hospitalization or prolongation of existing hospitalization;<br>• results in persistent or significant disability/ incapacity;<br>• results in a congenital anomaly/birth defect in the offspring of a patient who received the study drug;<br>• results from intentional or accidental drug overdose;<br>• or, results in cancer.
• **Unexpected Adverse Event:**	An AE, the nature or severity of which is not consistent with the applicable product information (e.g. Investigator Brochure content or the Summary of Product Characteristics).
• **Intensity:**	**Mild:** symptoms do not alter patient's/patient's normal functioning.<br>**Moderate:** symptoms produce some degree of impairment to function, but are not hazardous, uncomfortable or embarrassing to patient / patient.<br><br>**Severe:** symptoms definitely hazardous to well-being, significant impairment of functioning or incapacitation.
• **Causality:**	**Probable:** Reports including good reasons and sufficient documentation to assume a causal relationship in the sense of it being plausible, conceivable, likely, but not highly probable.<br>**Possible:** Reports containing sufficient information to indicate the possibility of a causal relationship in the sense of it being not impossible and not unlikely, although the connection may be uncertain or doubtful (e.g., because of missing data or insufficient evidence).<br>**Not Related:** Reports excluding the possibility of a relationship between the event and the drug treatment.<br>**Not Assessable:** Reports where causality is for one reason or another not assessable because of insufficient evidence, conflicting data or poor documentation.

11.2. Adverse events and reported procedures

It is the responsibility of the Investigator to document all AEs that occur during the study. At the beginning of each visit, and regularly during the visit, the subject will be questioned about any symptoms or unexpected occurrences since the previous visit, as follows: "Since the last

assessment, have you felt unwell or different from usual in any way?". All adverse events, regardless of severity or relationship to the study drug will be recorded on the AE Forms. All AEs must be reported regardless of whether or not they are considered to be drug-related.

Each event must be individually reported on the CRF, not combined (e.g. nausea and vomiting). The nature of each event, date and time of onset (where appropriate), duration of effect, severity and causality assessment should be established. Any consequent changes to the dosage schedule or any corrective therapy should be recorded.

AEs already recorded and designated as "continuing", should be reviewed at each subsequent assessment. If resolved, the details in the CRF should be completed. If an AE changes for the worse, in frequency of attacks/symptoms or in severity, a new record of the event must be filled (i.e., distinct reports are required for differing frequencies and/or severity of the same event to enable comprehensive safety reports and subsequent analysis to be made).

11.3. Investigator reporting requirements for serious adverse events

It is the responsibility of the Investigator to urgently notify the Sponsor representative of an event that is definitely or possibly classed as an SAE, regardless to the relationship or presumed relationship to the study medication, either by telephone or fax with all available details. The minimum information required is:

- patient initials and number,
- patient sex,
- protocol number,
- nature and seriousness of the event,
- causality assessment,
- reason for the serious classification,
- onset date,
- details of previous study medication dosing,
- outcome of the event (if available) and any action taken.

The Sponsor contact will provide instructions as to what documentation should be completed, but the initial written report (signed and dated) must be submitted (faxed), within 24 hours of SAE identification to the monitoring office responsible (as indicated on the front page of Protocol).

If additional details subsequently become available, they shall be submitted as soon as available, as an update of the initial report.

In the case of a SAE, the sponsor must be contacted immediately, preferably by the investigator. Refer to the emergency number(s) on the Cover Page of this protocol. If usual sponsor/monitoring contacts are not available, callers should specifically indicate that it is an emergency and speak to another member of the clinical research department.

All SAEs that are unexpected, and are either possibly or probably related to the investigational product will be promptly reported to the Ethical Committee which approved the protocol. Copies of all reports and associated correspondence need to be maintained with the study records.

11.4. Type and duration of the follow-up of subjects after adverse events

In all cases, the investigator must ensure that the patient receives medical follow-up as necessary until the condition has stabilised or returned to a normal state, even if the trial has concluded.

The Investigator should be able to supply the Sponsor representative (on request) with copies of all relevant results of examinations/treatments, etc., related to the medical follow-up of the adverse event.

In case of the occurrence of an adverse event, tests performed during the pre-inclusion visit may be repeated.

The concerned patient’s follow-up will continue until the complete resolution of the problem.

12. DIRECT ACCESS TO SOURCE DATA DOCUMENT

12.1. Access to source data

The sponsor shall be permitted to inspect the investigator center prior to commencement and during the course of the study to satisfy itself that the center is suitable and has the necessary facilities, staff and capacity to conduct the study. The investigator will ensure that his center has the necessary facilities, time and staff for conducting the study, and that these will be maintained for the duration of the study. The investigator will co-operate with the sponsor and any affiliated person to monitor or supervise the conduct of the study.

The study may be subjected to auditing by representatives of the sponsor and/or to inspection(s) by authorized representatives of local and/or foreign health authorities. In case of an audit or inspection, the investigator will be informed in advance.

The CRFs are to be made available for review by the clinical monitor or auditor or national regulatory inspectors. The investigators are required to give access to all source documents and study data in accordance with laws and regulations (articles L.1121-3 and R.5121-13 of the code of public health). The sponsor will not require the investigator or any member of their staff to take any action or be a party to any action which is contrary to the laws of the country in which the study is being carried out or to medical ethics.

12.2. Source document

The investigator agrees to allow direct access to source data of the study during monitoring visits, audits or inspections by authorized representatives of local and/or foreign health authorities.

Source documents (medical records, the original results of laboratory test …) are defined as any document proving the existence or the accuracy of a data or a registered event during the clinical study. It will be archived for 15 years by the investigator.

12.3. Confidentiality

In accordance with GCP and with the national data protection laws, all information concerning the subjects in the study must be treated as strictly confidential by all persons involved in the study including the clinical, medical and statistical monitor.

The investigators acknowledge that any information acquired from the sponsor or developed or acquired in connection with the study are strictly confidential and that they will not be disclosed to any third party nor use them for any purpose without first obtaining the written consent of the sponsor.

Such consent shall be deemed to have been given for disclosure to any person for whom the investigator is responsible at his center, but only so far as required for the purposes of the study, and, in the case of disclosures to staff, only if such staff is bound by obligations of confidentiality no less strict than those set out herein.

12.4. Archiving

The investigator must retain the subject identification codes for at least 15 years after completion or discontinuation of the study. Subject files and other source data must be kept for the maximum period of time permitted by the hospital, institution or private practice, but not less than 10 years, to meet international registration requirements. The investigator must

produce them or supply copies thereof to the sponsor or to the regulatory authorities upon demand, whilst ensuring subject confidentiality at all times.

12.5. Registration on the national register of volunteers

The healthy volunteers will be recorded on the national database of volunteers who participate to clinical researches, VRB (Volunteers for Biomedical Research) in accordance with the French regulations.

13. QUALITY CONTROL AND QUALITY ASSURANCE

The CRFs, containing all the clinical information, will be carefully checked both by the Investigators and the Monitors against the source documents according to the Good Clinical Practices Guideline (CPMP/ICH/135/95).

13.1. Commitment of the investigators and the sponsor

Investigators agree that the study is conducted in accordance with the Public Health law N° 2004-806 - 9 August 2004 on biomedical research, the implementing decree N° 2006-477 from 26/04/2006 amending chapter I of title II of book I of the first part of the code of Public Health related to biomedical research and the applicable orders.

The Good Clinical Practice (GCP) for biomedical research involving human drugs, referred to the Article L.1121-3 of the Code of public Health and the order of November 24, 2006 will also apply.

The study will be conducted in accordance with the ethical principles that have their origins in the Declaration of Helsinki (Somerset West, South Africa, 1996).

13.2. Quality insurance

The Clinical research assistant commissioned by the sponsor is responsible for inspection of the case report form at regular intervals, according to the monitoring plan of the study, throughout the study to ensure adherence to the protocol, compliance with the source documents, data consistency, and adherence to regulations on the conduct of clinical research.

The Clinical research assistant must have access to subject's medical file and other records related to the study required to verify the case report forms of the study.

Controls of consistency will be made by computer according to predefined rules. Requests for information or queries will be generated when possible errors are detected.

13.3. Quality control

The investigator is responsible for the authenticity of collected data as part of the study and accepts the legal provisions allowing the sponsor of the study to develop a quality control.
The investigator and coordinator agree to make themselves available for the monitoring visits. During these visits, the following documents will be reviewed:

- Informed consent
- Compliance with the protocol and procedures defined therein
- Quality of collected data in the case report forms: accuracy, missing data, data consistency with the source documents
- Product management

14. ETHICS CONSIDERATIONS

14.1. Independent Ethics Committee

The protocol and the Subject information form and consent will be submitted to The Independent Ethics Committee (CPP Sud-Est VI) and written approval from the Chair of the Ethics Committee is required before the initiation of the study.
The notification of the approval will be forwarded to the French authority AFSSAPS. A request for authorization will be sent by the sponsor to AFSSAPS before the start of the study.

14.2. Subject information and consent

Subjects will be informed fully and fairly, in understandable terms, about the objectives, the constraints of the study, the potentials risks involved, and monitoring measures, security, their rights to refuse to participate in the study and the possibility to withdraw at any time.

The investigator must also inform the subjects of the approval of the ethic committee.
All this information must be listed on the informed consent given to the subjects.
The informed and written consent of the subjects will be collected by the investigator. These documents are approved by the competent ethic committee and no other version shall be used.

Three copies will be co-signed by the investigator and the subject. A copy will be given to the subject, the original retained by the investigator and the third copy retained by the sponsor in a sealed envelope maintaining the confidentiality. This envelope will be archived during the legal period of 15 years.

14.3. Amendments to the protocol

There will be no alterations or changes to the protocol without agreement of all investigators and sponsor.
If such an agreement, the planned changes will constitute an amendment that will be attached to the protocol.
Any amendment must be notified to the ethic committee if the planned changes affect the ethical or medical-scientific study (evaluation criteria, addition of a new center ….). Minor modifications do not require a review of the ethic committee.

15. DATA HANDLING AND RECORD KEEPING

15.1. Case Report Forms (CRFs)

CRFs are records of data on each subject as defined by the study protocol. Entries on CRFs shall be made complete, legible and correct using a ball-point pen. Any mistakes shall be corrected by drawing a line over the old entry and by initialling and dating next to the correction. The last page of each visit shall be signed and dated by the investigator to indicate the overall responsibility.
Original completed CRFs will be collected by the sponsor for data entry and further data management and statistical applications. A copy of each CRF will be archived by the investigator. At each visit the clinical monitor will review all completed CRFs (since last visit) for completeness including signature, and will compare selected data with the subject’s records.

15.2. Entry and data processing

The data will be computerised in accordance with Data Protection. The coded data will be provided by the Clinical Investigation Center of Clermont-Ferrand.

15.3. CNIL

The data will be processed in accordance with the provisions of the Act of 6 August 2004 about protection of individual data with regard to the processing of personal data and amending Act of 6 January 1978 relating to computers, files and liberties.

16. FINANCING AND INSURANCE

The study will be covered under a liability insurance policy n°135372 in accordance with the Article L209-7 of the French Code of public health subscribed by the sponsor to SHAM society, in the case of an adverse event in relation to the study.

The sponsor will support the additional costs of any supplies or tests specifically required by the protocol.

17. COMMUNICATION – PUBLICATION RULES

The publications will be authorized after the end of the trial and the publication shall be agreed between the investigators and the partner.

Sponsor’s representative(s) have the option to be a named co-author(s) of any such publications, presentations or news releases. Sponsor retains the exclusive rights over the data resulting from the study for any purpose, including data from other participating institutions.

18. FEASABILITY OF THE STUDY

This clinical trial will be conducted by physician of Oncology departments and physicians of the Clinical Pharmacology center / Inserm-1405, University Hospital, Clermont-Ferrand, France.

The number of subjects required for each group (n=20) seems quite realistic in terms of recruitment capabilities of the teams involved in this project.

19. LIST OF ANNEXES

Annex 1 & 2: Informed consent

Annex 3: Study design

Annex 4: Table of evaluation for one patient

Annex 5: Summary of product characteristics / file experimental drug

Annex 6: Pharmacy authorization (Clermont-Ferrand University hospital)

Annex 7: Curriculum Vitae

Annex 8: Collection of serious adverse event file

Annex 9: Authorization of research places

Annex 10: CNIL

Annex 11: COMVAL authorization

Annex 12: Questionnaires

Annex 13: Booklet monitoring

20. ANNEXE 1 - INFORMED CONSENT DOCUMENT

PREVENTION DU DEVELOPPEMENT DE DOULEUR NEUROPATHIQUE POST-MASTECTOMIE / TUMORECTOMIE PAR L'ADMINISTRATION DE MEMANTINE EN PRE ET POST-CHIRURGIE

Promoteur
C.H.U. de Clermont-Ferrand
58, rue Montalembert - 63000 Clermont-Fd

Investigateur principal :
Dr Gisèle Pickering
CPC – CIC/INSERM 1405 - C.H.U. de Clermont-Fd
58, rue Montalembert - 63000 Clermont-Fd

Le Docteur …………………….. vous a proposé de participer à un protocole de recherche clinique, dont le CHU de Clermont-Ferrand est promoteur. Toutes les consultations que vous aurez à effectuer, dans le cadre de cette étude, se feront au sein du Centre de lutte contre le Cancer Jean Perrin et du Centre de Pharmacologie Clinique / Centre d'Investigation Clinique du CHU de Clermont-Ferrand.

Dans une étude clinique antérieure, il a été montré que certaines chirurgies étaient pourvoyeuses de douleurs chroniques appelées douleurs neuropathiques. Dans le cas de la chirurgie du sein, la survenue de douleur neuropathique est fréquente c'est-à-dire 37% des femmes ayant subi une mastectomie ressentent 3 à 6 mois après la chirurgie une douleur neuropathique.

La douleur neuropathique, quelles que soient son origine et sa localisation, présente des caractéristiques cliniques telles que perte de sensitivité, douleurs à type de brûlure, de coup de poignard, de décharge électrique, d'hyperalgésie (douleur accrue pour un stimulus douloureux). Les mécanismes responsables observés au cours de la douleur neuropathique, ne sont pas totalement clarifiés. Toutefois des arguments indiquent que les récepteurs au N-méthyl-D-aspartate (NMDA) jouent un rôle important dans ce mécanisme et représentent une cible primordiale pour le traitement de la douleur neuropathique.

Les récepteurs NMDA peuvent être bloqués de différentes manières par des molécules comme la mémantine qui en les bloquant permettraient de limiter ou même d'éviter les phénomènes douloureux.

L'objectif de cette étude est d'évaluer si la mémantine donnée pendant deux semaines avant la chirurgie (mastectomie pour tumorectomie avec ou sans curage) et maintenue pendant deux semaines après la chirurgie diminue l'intensité douloureuse à 3 mois et 6 mois après la chirurgie.

Paraphe du Médecin : **Paraphe du Sujet :**

Modalité de recrutement :

Ce protocole sera proposé à quarante patientes âgées de plus de 18 ans, ayant une mastectomie programmée répondant aux critères d'inclusion et d'exclusion. Les patientes seront incluses après signature du consentement éclairé.

Traitements administrés dans le cadre de cette étude:

Les Traitements administrés dans le cadre de cette étude sont la mémantine Ebixa® ou le Lactose.

Dans le cadre du protocole nous vous demanderons de :
- Réaliser des cotations de votre douleur grâce à une échelle numérique notée de 0 à 10 (0 = pas de douleur, 10 = maximum de douleur),
- Réaliser des cotations de la douleur neuropathique par les questionnaires NPSI et DN4,
- Compléter des questionnaires de qualité de vie,
- Effectuer des tests d'évaluations des fonctions cognitives

Echelle numérique : Cette échelle vous permet de coter votre douleur sur une graduation dont la note minimale est 0 et la note maximale est 10.

Questionnaire Neuropathic Pain Symptoms Inventory (N.P.S.I) : Auto-questionnaire spécialement conçu pour évaluer les différents symptômes de la douleur neuropathique.

Questionnaire Douleur Neuropathique (DN4) : Il permet d'estimer l'intensité de votre douleur neuropathique par le biais de 4 domaines définis en 10 items à cocher. L'investigateur vous interroge ou vous examine et remplit le questionnaire lui-même. Il note une réponse « oui » ou « non » à chaque item. Chaque « oui » vaut la note 1 et chaque « non » vaut la note 0.

Questionnaire concis sur les douleurs : Le Questionnaire Concis sur les Douleurs permet d'évaluer le retentissement de la douleur sur votre comportement quotidien en fonction des domaines suivants : humeur, capacité à marcher, travail habituel, relation avec les autres, sommeil, et goût de vivre.

Questionnaire Douleur de Saint Antoine : Ce questionnaire permet de qualifier les douleurs ressenties.

Echelle d'Anxiété Dépression (HAD) : Il s'agit d'un auto-questionnaire de 14 items complétés par vous-même qui permet de percevoir les émotions que vous éprouvez.

Questionnaire d'évaluation du sommeil de Leeds : Ce questionnaire est un auto-questionnaire qui traite quatre aspects de l'efficacité du sommeil : la qualité de l'endormissement, la qualité du sommeil, la qualité du réveil, la qualité de l'état suivant le réveil.

Paraphe du Médecin : **Paraphe du Sujet :**

Questionnaire qualité de vie SF36 : Cette échelle permet d'évaluer la santé physique et mentale en fonction de différents aspects de la santé (activités physiques, activités sociales, résistance morale, physique et émotionnelle pour accomplir les tâches quotidiennes, douleur physique, santé mentale générale, vitalité, perception de l'état de santé en général).

Impact cognitif des traitements grâce aux tests :

Trail Making Test A et B :
Ce test cognitif évalue les capacités de vitesse, les fonctions exécutives, l'attention, la concentration, la rapidité perceptive visuelle. Le TMT B fournit en plus une estimation de la flexibilité mentale (facilité de passer d'une série numérique à une série alphabétique).

Digit Symbol Substitution Test :
Le DSST est un test neuropsychologique qui évalue les déficits des fonctions cognitives, les atteintes cérébrales liées à l'âge et/ou la dépression. Il évalue également les capacités d'apprentissage (capacité à intégrer des associations nouvelles), la concentration, l'attention et les facultés de réalisation.

Déroulement de l'étude
La durée totale de votre participation à cette étude est de 7 mois maximum.
Dans le cadre de cette étude vous aurez à participer à 5 consultations auprès du médecin de l'essai et de 1 période d'hospitalisation postopératoire sur une période de 7 mois.
Un carnet journalier de suivi sera à compléter à partir du jour de votre opération.
L'un des traitements à l'étude (mémantine ou lactose) vous sera prescrit pour une durée de 4 semaines. La liste des traitements non-autorisés vous sera remise et vous devrez la conserver afin de la communiquer en cas de besoin.

Il vous est demandé de ne prendre aucun traitement antidépresseurs de type IMAO tout au long de l'étude. Lors de la première visite, vous devrez énoncer de manière exhaustive au médecin les traitements que vous prenez au long cours. En cas de nécessité pendant le protocole, vous devez tenir au courant le médecin des éventuels traitements pris.

Visite 1 : Visite d'annonce (Jour -15) : au Centre Jean Perrin
Suite à votre entretien avec votre médecin cancérologue, vous venez de planifier une chirurgie de mastectomie dans 2 semaines.
Lors de cet entretien, le médecin vous expliquera les objectifs de l'étude ainsi que les différents questionnaires et tests à effectuer de sorte que vous puissiez donner votre accord éclairé de participation. Vous êtes en droit de refuser de participer à ce protocole sans avoir à vous justifier et sans aucune conséquence sur la prise en charge de votre pathologie.

Si vous acceptez de participer à ce protocole et après avoir donné votre consentement, un examen clinique sera réalisé puis vous devrez compléter les questionnaires suivants : Questionnaire d'évaluation du sommeil de Leeds, Questionnaire qualité de vie SF36, le Trail Making Test A et B, et le Digit Symbol Substitution Test. Ces questionnaires et tests évaluent votre qualité de vie d'un point de vue anxiété, vie sociale, sommeil, et explorent certaines de vos fonctions cognitives telles que vos fonctions exécutives, votre attention, votre concentration, vos facultés de réalisation.

Paraphe du Médecin : **Paraphe du Sujet :**

Le médecin vous remettra un des deux traitements à l'étude (Mémantine ou Lactose) de façon aléatoire pour une période de deux semaines avant chirurgie et deux semaines après chirurgie soit au total 4 semaines de traitement. Le traitement à l'étude sera à prendre dès le lendemain matin, tous les jours pendant 4 semaines selon la date fixée de votre chirurgie.
Enfin, le médecin vous remettra un carnet de suivi journalier dans lequel vous aurez à coter quotidiennement votre douleur (à partir du jour de votre opération, c'est-à-dire le Jour 0, et jusqu'à votre visite à 3 mois) sur une échelle numérique notée de 0 à 10 (0 = pas de douleur, 10 = maximum de douleur), vous reporterez également les éventuels traitements antidouleur pris pendant cette durée.
Il est à noter que durant votre hospitalisation les professionnels de santé du service se chargeront de compléter votre carnet de suivi journalier. Une fois rentrée chez vous, vous le compléterez seule.

Tous les trois jours et ce jusqu'à la date de chirurgie, une personne de l'équipe projet (médecin ou infirmière ou attaché de recherche clinique), vous contactera par téléphone afin de vérifier que le traitement n'engendre aucun effet indésirable. Lors de ce contact, vous aurez à notifier tout événement anormal survenu depuis le début de prise du produit à l'essai. Si vous déclarez des effets et en fonction de leur intensité et de leur durée, le médecin pourra décider d'arrêter votre participation à l'essai ; dans le cas contraire, vous poursuivrez l'essai.

Enfin, un courrier reprenant les termes de l'essai, la liste des traitements non-autorisés, la liste des contacts des médecins CJP / CIC sera envoyé à votre médecin traitant afin de le tenir au courant de votre inclusion dans l'essai et de la marche à suivre en cas d'effet indésirable.

Visite 2 : Visite Anesthésie / Douleur (Jour -4 +/-3 jours) : au Centre Jean Perrin
Au maximum une semaine avant l'opération chirurgicale, vous devrez venir au CJP en consultation pré-anesthésique qui sera couplée avec une consultation Douleur.
Lors de cette consultation, un bilan clinique sera réalisé par un anesthésiste en prévision de l'anesthésie générale que vous subirez le jour de l'opération et un bilan de votre statut douloureux avant opération. Il sera vérifié la présence (ou non) de douleurs existantes ainsi que leurs caractéristiques.

Visite 3 : Hospitalisation pour Chirurgie de Mastectomie - Jour 0 : au Centre Jean Perrin
Le Jour 0 correspond au jour de votre opération chirurgicale de mastectomie pour tumorectomie.
Après l'opération, vous continuerez à prendre votre traitement pendant 2 semaines (du Jour 0 jusqu'au Jour 15).

Lors de votre hospitalisation, avec l'aide des professionnels de santé vous évaluerez plusieurs fois par jour votre douleur par cotation sur une échelle numérique notée de 0 à 10 (0 = pas de douleur, 10 = maximum de douleur), les données seront reportées sur votre carnet de suivi journalier.
Votre consommation en traitement antidouleur sur cette période sera elle aussi reportée sur votre carnet de suivi.
Au cinquième ou sixième jour après chirurgie, une consultation Douleur sera effectuée afin d'évaluer votre statut douloureux (apparition de nouvelles douleurs ou évolution de douleurs préexistantes).

Paraphe du Médecin : **Paraphe du Sujet :**

Visite 4 : Visite de suivi - Jour 16 : au Centre Jean Perrin ou Centre d'Investigation Clinique

Au Jour 16 vous devrez compléter les questionnaires DN4 et NPSI et réaliser le Trail Making Test A et B, et le Digit Symbol Substitution Test.

Période de Jour 17 à 3mois : A domicile

Pendant cette période, vous évaluerez quotidiennement votre douleur par cotation sur une échelle numérique de 0 à 10 (0 = pas de douleur, 10 = maximum de douleur), vous reporterez les données sur votre carnet de suivi journalier. Vous reporterez également sur votre carnet de suivi journalier votre consommation en traitement antidouleur.

A partir de votre retour au domicile et jusqu'à la fin de l'essai, un contact téléphonique hebdomadaire sera réalisé par le CIC afin de relever d'éventuels effets indésirables. Vous devrez en dehors de ces appels téléphoniques programmés, contacter le CIC et/ou votre médecin traitant si des effets apparaissent entre deux appels.

Visite 5 – Visite de suivi à 3 mois de votre opération : Au Centre d'Investigation Clinique

Le jour de la visite vous ramènerez les traitements vides utilisés et non-utilisés ainsi que le carnet de suivi journalier au Centre de Pharmacologie Clinique, ce qui permettra au médecin investigateur d'observer l'évolution de l'échelle numérique, ainsi que la prise d'antidouleur depuis votre opération (Jour 0).

Après un examen clinique réalisé par votre médecin investigateur, vous devrez coter votre douleur moyenne ressentie sur les 5 jours précédant cette visite sur une échelle numérique de 0 à 10 (0 = pas de douleur, 10 = maximum de douleur).

Ensuite vous devrez compléter les questionnaires suivants : Douleurs Neuropathiques DN4, Questionnaire concis sur les douleurs QCD, Questionnaire de Saint-Antoine QDSA, échelle HAD, Questionnaire d'évaluation du sommeil de Leeds, Questionnaire qualité de vie SF 36, Questionnaire Neuropathic Pain Symptom Inventory NPSI.

Et de la même façon, vous devrez réaliser les tests cognitifs Trail Making Test A et B, et Digit Symbol Substitution Test.

Visite 6 - Visite de suivi à 6 mois de votre opération : Au Centre d'Investigation Clinique

Cette visite est identique à la précédente hormis que vous n'aurez rien à rapporter, puisque aucun carnet de suivi journalier n'est à compléter entre le visite à 3 mois et la visite à 6 mois. Cette visite représente la visite de fin d'étude.

Les bénéfices / Risques attendus

Si vous acceptez de participer, ce protocole vous apportera un suivi amélioré de votre douleur, avec des visites trimestrielles régulières et une évaluation de votre état de santé globale et de votre qualité de vie avec le traitement prescrit.

Paraphe du Médecin : **Paraphe du Sujet :**

La mémantine est prescrite dans la maladie d'Alzheimer et la posologie choisie dans cet essai est celle de cette pathologie, à 20 mg/jour, la posologie suivra une augmentation par palier de la dose tous les 3 à 5 jours pendant les deux ou trois premières semaines afin d'arriver à la dose souhaitée de 20 mg/jour. La littérature à ce sujet rapporte chez des patients souffrant de douleur neuropathique des doses beaucoup plus élevées (à 55 mg/jour) et sans que des effets indésirables graves aient été rapportés.
Les effets indésirables attendus de cette molécule sont d'intensité légère à modérée : vertiges, céphalées, somnolence, nausées, vomissement, constipation, réactions allergiques, hypertension.
Aucun effet indésirable n'a été rapporté pour l'administration de lactose à la dose utilisée dans ce protocole. Cependant, certaines intolérances au lactose ont été décrites mais à des doses fortement supérieure à celle utilisée ici.

Indemnisation
Vous recevrez une indemnisation de 200€ pour la totalité de l'essai. Dans le cas d'un arrêt prématuré de l'essai, un calcul de l'indemnisation sera effectué au prorata des visites effectuées (50€ si arrêt en visite 2, 100€ si arrêt en visite 4, 150€ si arrêt en visite 5).

Période d'exclusion pour participer à une autre recherche biomédicale
Vous n'êtes pas autorisé à participer à un autre essai clinique durant toute la durée du protocole.
De plus, vous ne pourrez participer à un autre protocole de recherche clinique pendant 1 semaine après la fin de ce protocole.

Généralités
Le CHU de Clermont-Ferrand, qui organise cette recherche biomédicale en qualité de promoteur, a contracté une assurance conformément aux dispositions législatives, garantissant sa responsabilité civile et celle de tout intervenant auprès de la Société Hospitalière d'Assurances Mutuelles (SHAM, contrat n°135372). Dans le cas où votre état de santé serait altéré du fait de votre participation à l'étude, conformément à la loi de Santé Publique n°2004-806 du 9 août 2004, vous seriez en droit de recevoir des dédommagements dans le cadre de ce contrat d'assurance spécifique.

Cette recherche a reçu l'avis favorable du Comité de Protection des Personnes Sud Est VI le 13/12/2011 ainsi que l'autorisation préalable de l'autorité compétente de santé datée du 07/12/2011. Il est possible que cette recherche soit interrompue, si les circonstances le nécessitent, par le promoteur ou à la demande de l'autorité de santé.

Dans le cadre de la recherche biomédicale à laquelle le CHU de Clermont-Ferrand vous propose de participer, un traitement informatique de vos données personnelles va être mis en œuvre pour permettre d'analyser les résultats de la recherche au regard de l'objectif de cette dernière qui vous a été présenté.

Les informations relatives à l'étude recueillies par l'investigateur sont traitées confidentiellement. En accord avec la loi Informatique et Liberté, le nom des sujets est automatiquement remplacé par un numéro de code dont la correspondance est connue des seuls médecins investigateurs.

Paraphe du Médecin : **Paraphe du Sujet :**

Les données feront l'objet d'un traitement informatisé anonyme et leur consultation sera autorisée aux collaborateurs participant à la recherche, désignés par le promoteur et éventuellement au représentant des autorités de santé.

Conformément aux dispositions de la loi relative à l'informatique, aux fichiers et aux libertés, vous disposez d'un droit d'accès et de rectification auprès du médecin qui vous suit dans le cadre de la recherche. Vous disposez également d'un droit d'opposition à la transmission des données couvertes par le secret professionnel susceptibles d'être utilisées dans le cadre de cette recherche et d'être traitées.

Vous pouvez également accéder directement ou par l'intermédiaire d'un médecin de votre choix à l'ensemble de vos données médicales en application des dispositions de l'article L. 1111-7 du code de la santé publique. Ces droits s'exercent auprès du médecin qui vous suit dans le cadre de la recherche et qui connaît votre identité.

Conformément à l'article L 1121-16 du Code de la Santé Publique, vous serez inscrit dans le Fichier National des personnes qui se prêtent à des recherches biomédicales.

Conformément à l'article L1111-6 de la loi n°2002-303 du 04 mars 2002 relative aux droits des malades et à la qualité du système de Santé, vous avez la possibilité de nommer une personne de confiance qui peut être un parent, un proche ou votre médecin traitant afin que cette personne vous accompagne tout au long de la Recherche dans vos démarches et assiste aux entretiens médicaux afin de vous aider dans vos décisions.
Nom et qualité de la personne de confiance (le cas échéant) : ____

Vous êtes libre d'accepter ou de refuser de participer à cette recherche. De plus vous pouvez exercer à tout moment votre droit de retrait de cette recherche. Vous pouvez demander à tout moment des explications complémentaires sur l'étude à l'équipe soignante.
Par ailleurs, vous pourrez être tenu informé des résultats globaux de cette recherche à la fin de l'étude.

Lorsque vous aurez lu cette note d'information et obtenu les réponses aux questions que vous vous posez en interrogeant le médecin investigateur, il vous sera proposé, si vous en êtes d'accord, de donner votre consentement écrit en signant le document préparé à cet effet.

Paraphe du Médecin

Date : ……/……/……

Signature du Sujet
Précédée de la mention « Lu et compris »

21. ANNEXE 2 - FORMULAIRE DE CONSENTEMENT DE PARTICIPATION A UNE RECHERCHE BIOMÉDICALE

PREVENTION DU DEVELOPPEMENT DE DOULEUR NEUROPATHIQUE POST-MASTECTOMIE / TUMORECTOMIE PAR L'ADMINISTRATION DE MEMANTINE EN PRE ET POST-CHIRURGIE

Investigateur principal:
Dr Gisèle Pickering
CPC – CIC/INSERM 1405
C.H.U. de Clermont-Ferrand
58, rue Montalembert - 63000 Clermont-Ferrand

Je soussigné(e) M. (*nom, prénom*)……………………………………Né(e) le _ _ / _ _ / _ _ _ _
Demeurant……………………………………………………………………………………….
Déclare : que le Docteur (*nom, prénom, téléphone*) ………………………………………………
m'a proposé de participer à l'étude sus nommée, qu'il m'a expliqué en détail le protocole, qu'il m'a notamment fait connaître :

- l'objectif, la méthode et la durée de l'étude
- les contraintes et les risques potentiels encourus
- mon droit de refuser de participer et de retirer mon consentement à tout moment sans avoir à me justifier
- mon obligation d'inscription à un régime de sécurité sociale
- que, si je le souhaite, à son terme, je serais informé(e) par le médecin investigateur de ses résultats globaux
- que je ne serai pas autorisé(e) à participer à d'autres études cliniques pendant une durée de une semaine.
- que le Comité de Protection des Personnes Sud Est VI a émis un avis favorable le 13/12/2011 ainsi que l'autorisation préalable de l'autorité compétente de santé datée du 07/12/2011.
- que dans le cadre de cette étude le promoteur, le CHU de Clermont-Ferrand, a souscrit à une assurance couvrant cette recherche.

Les informations relatives à l'étude recueillies par l'investigateur sont traitées confidentiellement. J'accepte que ces données puissent faire l'objet d'un traitement informatisé anonyme. J'ai bien noté que le droit d'accès prévu par la loi du 6 août 2004 relative à l'informatique, aux fichiers et aux libertés s'exerce à tout moment auprès du médecin qui me suit dans le cadre de la recherche et qui connaît mon identité. Je pourrai exercer mon droit de rectification et d'opposition auprès de ce même médecin, qui contactera le promoteur de la recherche.
J'accepte mon inscription dans le Fichier National des personnes qui se prêtent à des recherches biomédicales (Art. L 1121-16 du Code de la Santé Publique).

Après avoir discuté librement et obtenu réponse à toutes mes questions, j'accepte librement et volontairement de participer à cette recherche biomédicale dans les conditions précisées dans le formulaire d'information et de consentement.

Nom et prénom du sujet :………………………………..	Nom de l'investigateur : …………………
Date :……./……./…….	Date :……./……./…….
Signature *précédée de la mention « Lu et compris » :*	Signature :

Ce document est à réaliser en 3 exemplaires, dont l'original doit être conservé par l'investigateur, la première copie doit être remise à la personne donnant son consentement et la deuxième copie doit être conservée par le promoteur

22. ANNEXE 3 – SCHEMA DE L'ETUDE

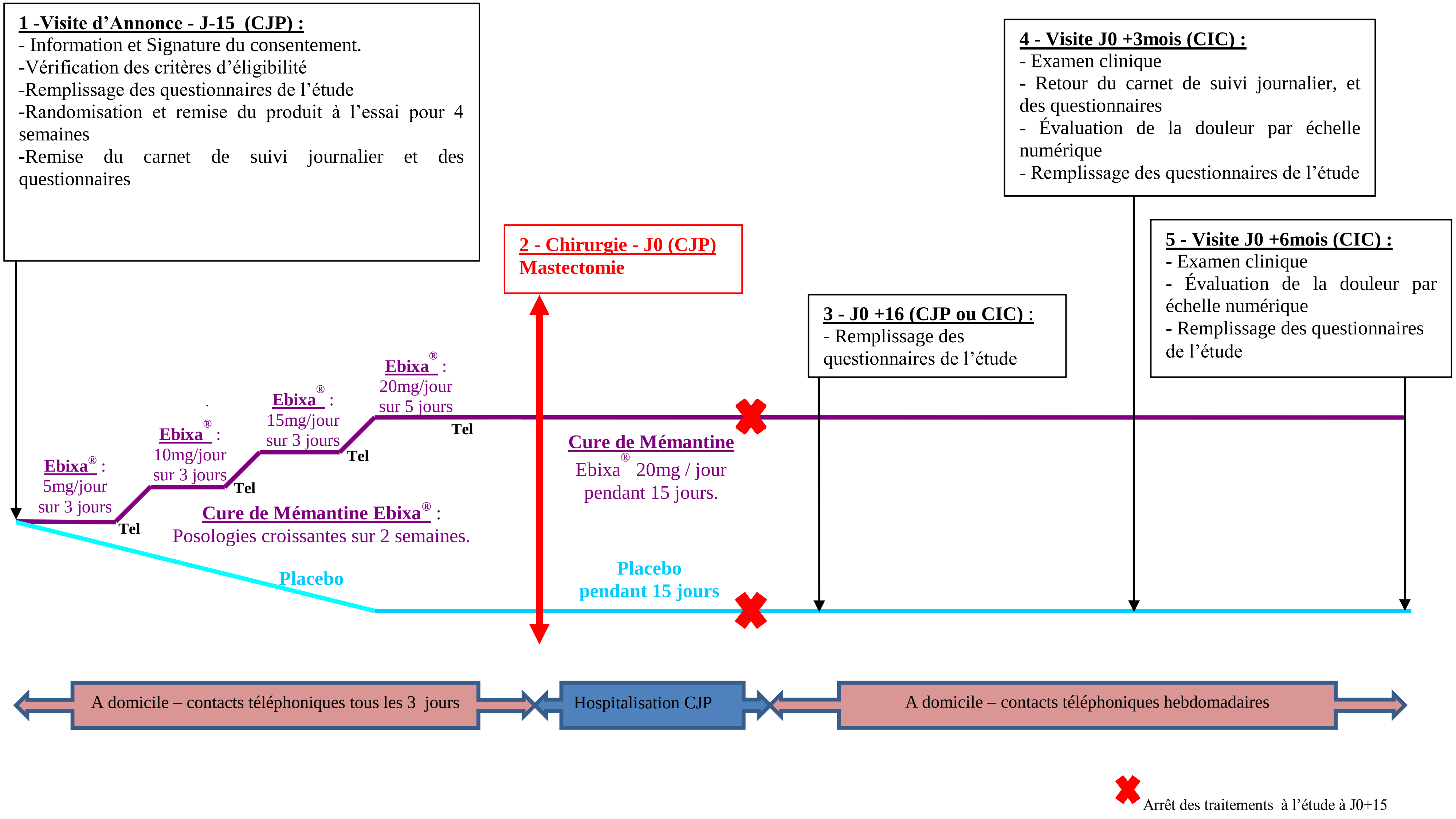

23. ANNEXE 4 – TABLEAU DES EVALUATIONS POUR UN SUJET

Visits	Annoucement	Surgery	Hospitalization	Follow-up	Follow-up	Follow-up
Days of visit	D0-15	D0	D0 to D0+15	D0+16	D0 +3months	D0 +6months
Center	CJP	CJP	CJP	CJP or CIC	CIC	CIC
Informed consent	+					
Checking inclusion and non-inclusion criteria	+					
Clinical examination	+	+			+	+
Mastectomy		+				
Hospitalization			+			
Filling out questionnaires:						
- Leeds, SF36, TMT A and B, DSST	+					
- DN4, NPSI, TMT A and B, DSST				+		
- DN4, QCD, QDSA, Leeds, SF36, NPSI, TMT A and B, DSST					+	+
Treatment pre and post-surgery	+					
Phone-call – adverse event collection	+	+		+	+	+

24. ANNEXE 5 - RÉSUMÉ DES CARACTÉRISTIQUES PRODUITS / DOSSIER DU MEDICAMENT EXPERIMENTAL

RÉSUMÉ DES CARACTÉRISTIQUES PRODUITS

MEMANTINE
EBIXA

Répertoire des spécialités EMEA

RCP EBIXA en pièce jointe.

Répertoire des spécialités AFSSAPS<br>Extrait – Liste des documents de référence listé pour EBIXA<br>RCP EBIXA Non disponible

Extrait - Liste des documents de référence mis à jour le 26/11/2010
CIS : 6 172 111 8
Dénomination de la spécialité
EBIXA 5 mg + 10 mg + 15 mg + 20 mg, comprimé pelliculé

Composition en substances actives

Comprimé de 5 mg
Composition pour un comprimé
MEMANTINE BASE : 4,15 mg sous forme de : chlorhydrate de mémantine : 5 mg

Comprimé de 10 mg
Composition pour un comprimé
MEMANTINE BASE : 8,31 mg sous forme de : mémantine (chlorhydrate de) : 10 mg

Comprimé de 15 mg
Composition pour un comprimé MEMANTINE BASE : 12,46 mg sous forme de : mémantine (chlorhydrate de) 15 mg

Comprimé de 20 mg
Composition MEMANTINE BASE : 16,62 mg sous forme de : mémantine (chlorhydrate de) 20 mg

Titulaire(s) de l'AMM
H. LUNDBECK A/S : Depuis le : **08/05/2008**

Données administratives
Date de l'AMM : **08/05/2008**
Procédure centralisée - **Site de l'EMEA**
Statut de l'AMM : **VALIDE**

Conditions de prescription et de délivrance
liste I
médicament nécessitant une surveillance particulière pendant le traitement
prescription initiale annuelle réservée à certains spécialistes
prescription réservée aux spécialistes autorisés RADIOPHARMACEUTIQUES
prescription réservée aux spécialistes et services GERIATRIE
prescription réservée aux spécialistes et services NEUROLOGIE
prescription réservée aux spécialistes et services PSYCHIATRIE

Présentations
491472-5
1 plaquette(s) thermoformée(s) aluminium polypropylène de 7 comprimé(s) - 1 plaquette(s) thermoformée(s) aluminium polypropylène de 7 comprimé(s) - 1 plaquette(s) thermoformée(s) aluminium polypropylène de 7 comprimé(s) - 1 plaquette(s) thermoformée(s) al

Déclaration de commercialisation non communiquée

VIDAL 2010
Résumé Caractéristiques Produits
EBIXA®
Mémantine

VIDAL 2010 Médicaments Mise à jour du 03/05/2010

FORMES et PRÉSENTATIONS

Comprimé pelliculé à 10 mg (oblong, conique selon l'axe central, biconvexe, avec une ligne de section unique sur les deux faces * *; blanc à blanc cassé) :* Boîte de 56, sous plaquettes thermoformées.
Comprimé pelliculé à 20 mg (oblong, ovale, portant l'inscription « 20 » sur une face et l'inscription « MEM » sur l'autre ; rouge pâle à gris-rouge) : Boîte de 28, sous plaquettes thermoformées.
Modèles hospitaliers : Boîtes de 98, sous plaquettes thermoformées prédécoupées (conditionnements unitaires).
Solution buvable en gouttes à 10 mg/g (transparente et incolore à légèrement jaunâtre) : Flacon de 50 g, avec pompe doseuse.
* Le comprimé de 10 mg peut être divisé en deux demi-doses égales.

COMPOSITION

Comprimé :		*p cp*
Mémantine (DCI) chlorhydrate		10 mg
	ou	20 mg
(soit en mémantine : 8,31 mg/cp à 10 mg ; 16,62 mg/cp à 20 mg)		

Excipients :

Comprimé à 10 mg : Noyau : lactose monohydraté, cellulose microcristalline, silice colloïdale anhydre, talc, stéarate de magnésium. *Pelliculage :* copolymère d'acide méthacrylique-acrylate d'éthyle (1 : 1), sulfate sodique de lauryle, polysorbate 80, talc, triacétine, émulsion de siméticone.

Comprimé à 20 mg : Noyau : cellulose microcristalline, croscarmellose sodique, silice colloïdale anhydre, stéarate de magnésium. *Pelliculage :* hypromellose, macrogol 400, dioxyde de titane (E 171), oxydes de fer rouge et jaune (E 172).

Teneur en lactose : 166 mg/cp à 10 mg.

Solution buvable :	*p pression* *
Mémantine (DCI) chlorhydrate	5 mg
(soit en mémantine : 4,16 mg/pression)	

Excipients : sorbate de potassium, sorbitol (E 420), eau purifiée.

Teneur en sorbitol : 100 mg/g.

Teneur en potassium : 0,5 mg/g.

* Chaque activation de la pompe (une pression) délivre 0,5 ml (0,5 g) de solution.

DC INDICATIONS

Traitement des patients atteints d'une forme modérée à sévère de la maladie d'Alzheimer.

DC POSOLOGIE ET MODE D'ADMINISTRATION

Le traitement doit être initié et supervisé par un médecin entraîné au diagnostic et au traitement de la maladie d'Alzheimer au stade démentiel. Le traitement ne doit commencer qu'avec l'assurance de la disponibilité d'un auxiliaire de soins qui surveillera régulièrement la prise du médicament par le patient. Le diagnostic doit être établi selon les critères en vigueur.
Ebixa doit être pris une fois par jour, à la même heure chaque jour. Les comprimés pelliculés ou la solution peuvent être pris pendant ou en dehors des repas. La solution ne doit pas être versée ou pompée directement dans la bouche à partir du flacon ou de la pompe mais elle doit être dosée dans une cuillère ou dans un verre d'eau en utilisant la pompe (pour des instructions détaillées sur la préparation et la manipulation de la solution buvable, cf Modalités manipulation/élimination).
Adultes :

Progression posologique :
La dose maximale recommandée est de 20 mg par jour. Pour réduire le risque d'effets indésirables, cette dose est atteinte par une progression posologique de 5 mg par semaine au cours des trois premières semaines, en procédant comme suit :

- Semaine 1 (jours 1-7) : le patient doit prendre la moitié d'un comprimé pelliculé de 10 mg ou 0,5 ml de solution (soit 5 mg, équivalant à 1 pression) par jour pendant 7 jours.
- Semaine 2 (jours 8-14) : le patient doit prendre un comprimé pelliculé de 10 mg ou 1 ml de solution (soit 10 mg, équivalant à 2 pressions) par jour pendant 7 jours.
- Semaine 3 (jours 15-21) : le patient doit prendre un comprimé pelliculé et demi de 10 mg ou 1,5 ml de solution (soit 15 mg, équivalant à 3 pressions) par jour pendant 7 jours.
- À partir de la semaine 4 : le patient doit prendre deux comprimés pelliculés de 10 mg (ou un comprimé pelliculé de 20 mg) ou 2 ml de solution (soit 20 mg, équivalant à 4 pressions) par jour.
- Dose d'entretien : la dose d'entretien recommandée est de 20 mg par jour.

Coût du traitement journalier : 3,17 euro(s) (cp à 10 mg et à 20 mg) ; 3,19 euro(s) (sol buv).

Personnes âgées :
Sur la base des études cliniques, la dose recommandée pour les patients de plus de 65 ans est de 20 mg par jour (2 comprimés de 10 mg ou 1 comprimé de 20 mg) ou de 2 ml de solution (équivalant à 4 pressions), comme décrit ci-dessus.
Enfants et adolescents :
Ebixa ne doit pas être utilisé chez l'enfant de moins de 18 ans en raison d'un manque de données concernant la sécurité et l'efficacité.
Insuffisance rénale :
Chez les patients présentant une insuffisance rénale légère (clairance de la créatinine comprise entre 50 et 80 ml/min), aucune adaptation posologique n'est requise. Chez les patients présentant une insuffisance rénale modérée (clairance de la créatinine comprise entre 30 et 49 ml/min), la dose quotidienne doit être de 10 mg ou de 1 ml de solution (équivalant à 2 pressions). Si la tolérance est bonne après au moins 7 jours de traitement, la dose pourra être augmentée jusqu'à 20 mg par jour en suivant le schéma de progression posologique habituel. Chez les patients présentant une insuffisance rénale sévère (clairance de la créatinine comprise entre 5 et 29 ml/min), la dose quotidienne doit être de 10 mg ou de 1 ml de solution (équivalant à 2 pressions).
Insuffisance hépatique :
Chez les patients présentant une insuffisance hépatique légère à modérée (Child-Pugh A et Child-Pugh B), aucune adaptation posologique n'est nécessaire. Aucune donnée concernant l'utilisation de la mémantine chez les patients présentant une insuffisance hépatique sévère n'est disponible. L'administration d'Ebixa n'est pas recommandée chez ce type de patients.

DC CONTRE-INDICATIONS

Hypersensibilité à la substance active ou à l'un des excipients.

DC MISES EN GARDE et PRÉCAUTIONS D'EMPLOI

- La prudence est recommandée chez les patients épileptiques, ayant des antécédents de convulsions, ou chez les patients présentant des facteurs de risque d'épilepsie.
- L'association aux antagonistes NMDA (N-méthyl-D-aspartate) tels que l'amantadine, la kétamine ou le dextrométhorphane doit être évitée. Ces composés agissent au niveau des mêmes récepteurs que la mémantine et, par conséquent, les effets indésirables (essentiellement liés au système nerveux central [SNC]) peuvent être plus fréquents ou plus prononcés (cf Interactions).
- Certains facteurs susceptibles d'augmenter le pH de l'urine (cf Pharmacocinétique : Élimination) peuvent exiger une surveillance étroite du patient. Ces facteurs incluent des modifications radicales du régime alimentaire, par exemple le passage d'un régime carné à un régime végétarien, ou l'ingestion massive de tampons gastriques alcalinisants. Le pH de l'urine peut également être élevé lors d'états d'acidose tubulaire rénale (ATR) ou d'infection urinaire sévère à Proteus.
- Dans la majorité des essais cliniques, les patients avec infarctus du myocarde récent, insuffisance cardiaque congestive non compensée (NYHA III-IV) ou hypertension artérielle non contrôlée étaient exclus. Par conséquent, les données disponibles sont limitées et les patients présentant ces pathologies doivent être étroitement surveillés.
- Excipients :

- Les comprimés à 10 mg contiennent du lactose monohydraté. Les patients présentant une intolérance au galactose, un déficit en lactase de Lapp ou un syndrome de malabsorption du glucose et du galactose (maladies héréditaires rares) ne doivent pas prendre ce médicament.

- La solution buvable contient du sorbitol. Les patients présentant une intolérance au fructose (maladie héréditaire rare) ne doivent pas prendre ce médicament.

DC INTERACTIONS

Étant donné les effets pharmacologiques et le mode d'action de la mémantine, les interactions suivantes sont possibles :

- Le mode d'action suggère que les effets de la L-dopa, des agonistes dopaminergiques et des anticholinergiques peuvent être augmentés par un traitement associé avec des antagonistes NMDA tels que la mémantine. Les effets des barbituriques et des neuroleptiques peuvent être diminués. L'association de mémantine aux agents antispastiques, dantrolène ou baclofène, peut modifier leurs effets et un ajustement posologique de ces produits peut s'avérer nécessaire.

- L'association de mémantine et d'amantadine doit être évitée en raison du risque de psychose pharmacotoxique. Les deux composés sont des antagonistes NMDA chimiquement proches. C'est peut-être également le cas de la kétamine et du dextrométhorphane (cf Mises en garde/Précautions d'emploi). Il existe un cas publié concernant aussi un risque possible d'interaction lié à l'association mémantine et phénytoïne.

- D'autres substances actives telles que la cimétidine, la ranitidine, le procaïnamide, la quinidine, la quinine et la nicotine, qui utilisent le même système de transport cationique rénal que l'amantadine, pourraient également interagir avec la mémantine, entraînant une possible augmentation des taux plasmatiques.

- Il existe un risque de réduction des taux plasmatiques d'hydrochlorothiazide (HCT) lorsque la mémantine est administrée avec l'HCT ou toute association en contenant.

- Lors du suivi de pharmacovigilance après commercialisation, des cas isolés d'augmentation de l'INR (Rapport Normalisé International) ont été rapportés chez des patients traités de façon concomitante avec la warfarine. Bien qu'aucun lien de causalité n'ait été établi, une surveillance étroite du taux de prothrombine ou de l'INR est recommandée chez les patients traités de façon concomitante avec des anticoagulants oraux.

Dans des études de pharmacocinétique (PK) à dose unique chez des sujets sains jeunes, aucune interaction significative entre substances actives n'a été observée entre la mémantine et l'association glibenclamide/metformine ou le donépézil.
Dans une étude clinique chez des sujets sains jeunes, aucun effet significatif de la mémantine sur la pharmacocinétique de la galantamine n'a été observé.
In vitro, la mémantine n'a pas inhibé les CYP 1A2, 2A6, 2C9, 2D6, 2E1, 3A, la flavine mono-oxygénase, l'époxyde hydrolase ou la sulfatation.

DC GROSSESSE et ALLAITEMENT

Pour la mémantine, aucune donnée clinique sur les grossesses exposées n'est disponible. Les études chez l'animal indiquent un potentiel de réduction du développement intra-utérin à des niveaux d'exposition identiques ou légèrement supérieurs à l'exposition humaine (cf Sécurité préclinique). Le risque demeure inconnu pour l'être humain. La mémantine ne doit pas être utilisée durant la grossesse, sauf cas de nécessité absolue.
Il n'a pas été établi si la mémantine est excrétée dans le lait maternel humain mais, étant donné la lipophilie de la substance, le passage est probable. Il est déconseillé aux femmes prenant de la mémantine d'allaiter.

DC CONDUITE et UTILISATION DE MACHINES

Une maladie d'Alzheimer modérée à sévère a généralement un impact important sur l'aptitude à conduire des véhicules et à utiliser des machines. De plus, Ebixa exerce une influence mineure à modérée sur l'aptitude à conduire des véhicules et à utiliser des machines ; les patients ambulatoires doivent donc être avertis de prendre des précautions particulières.

DC EFFETS INDÉSIRABLES

Au cours des essais cliniques dans la démence légère à sévère ayant inclus 1784 patients traités par Ebixa et 1595 patients sous placebo, la fréquence globale des événements indésirables pour Ebixa ne différait pas de celle du placebo ; les événements indésirables étaient en général d'intensité légère à modérée. Les événements indésirables les plus fréquents avec une incidence supérieure dans le groupe Ebixa par rapport au groupe placebo ont été : sensations vertigineuses (6,3 % vs 5,6 %, respectivement), céphalée (5,2 % vs 3,9 %), constipation (4,6 % vs 2,6 %), somnolence (3,4 % vs 2,2 %) et hypertension (4,1 % vs 2,8 %).
Les effets indésirables dans le tableau ci-dessous ont été recueillis au cours des essais cliniques avec Ebixa et depuis sa commercialisation. Au sein de chaque groupe de fréquence, les effets indésirables sont présentés

suivant un ordre décroissant de gravité.
Les effets indésirables sont classés par classes systèmes organes en appliquant les conventions suivantes : très fréquent (>= 1/10), fréquent (>= 1/100, < 1/10), peu fréquent (>= 1/1000, < 1/100), rare (>= 1/10 000, < 1/1000), très rare (< 1/10 000), fréquence indéterminée (ne peut être estimée sur la base des données disponibles).

Infections et infestations	Peu fréquent	Infections fongiques
Affections psychiatriques	Fréquent	Somnolence
	Peu fréquent	Confusion, hallucinations*
	Fréquence indéterminée	Réactions psychotiques**
Affections du système nerveux	Fréquent	Sensations vertigineuses
	Peu fréquent	Troubles de la marche
	Très rare	Convulsions
Affections cardiaques	Peu fréquent	Insuffisance cardiaque
Affections vasculaires	Fréquent	Hypertension
	Peu fréquent	Thrombose veineuse/thromboembolisme
Affections respiratoires, thoraciques et médiastinales	Fréquent	Dyspnée
Affections gastro-intestinales	Fréquent	Constipation
	Peu fréquent	Vomissements
	Fréquence indéterminée	Pancréatite**
Troubles généraux et anomalies au site d'administration	Fréquent	Céphalée
	Peu fréquent	Fatigue

* Les hallucinations ont été observées principalement chez les patients au stade sévère de la maladie d'Alzheimer.
** Cas isolés rapportés au cours du suivi de pharmacovigilance.
La maladie d'Alzheimer a été associée à des cas de dépression, d'idées suicidaires et de suicide. Lors du suivi de pharmacovigilance après commercialisation, ces événements ont été rapportés chez des patients traités par Ebixa.

DC SURDOSAGE

Les données concernant le surdosage au cours des essais cliniques et du suivi de pharmacovigilance après commercialisation sont limitées.
Symptômes :

Des surdosages relativement importants (200 mg et 105 mg par jour pendant 3 jours, respectivement) ont été associés soit aux seuls symptômes fatigue, faiblesse et/ou diarrhée, soit à l'absence de symptômes. Dans les cas de surdosages inférieurs à 140 mg ou dont la dose est inconnue, les patients ont présenté des troubles du système nerveux central (confusion, sensation ébrieuse, somnolence, vertige, agitation, agressivité, hallucination et troubles de la marche) et/ou des troubles gastro-intestinaux (vomissement et diarrhée).
Dans le cas le plus extrême de surdosage, le patient a survécu à la prise orale totale de 2000 mg de mémantine et présenté des troubles du système nerveux central (10 jours de coma suivis d'une diplopie et d'une agitation). Le patient a reçu un traitement symptomatique et des plasmaphérèses. Le patient a guéri sans séquelles permanentes.
Dans un autre cas de surdosage important, le patient a également survécu et guéri. Le patient avait reçu 400 mg de mémantine par voie orale. Le patient a présenté des troubles du système nerveux central tels qu'hyperactivité motrice, psychose, hallucinations visuelles, état proconvulsif, somnolence, stupeur et perte de connaissance.

Traitement :

En cas de surdosage, le traitement doit être symptomatique. Aucun antidote spécifique en cas d'intoxication ou de surdosage n'est disponible. Des moyens de prise en charge habituels pour éliminer la substance active tels que lavage gastrique, charbon activé (interruption d'un potentiel cycle entérohépatique), acidification des urines, diurèse forcée doivent être utilisés en fonction des besoins.
En cas de signes et de symptômes d'hyperstimulation générale du système nerveux central (SNC), un traitement symptomatique sous étroite surveillance doit être envisagé.

PP PHARMACODYNAMIE

Classe pharmacothérapeutique : autres médicaments antidémence (code ATC : N06DX01).

Il apparaît de plus en plus clairement que le dysfonctionnement de la neurotransmission glutamatergique, en particulier au niveau des récepteurs NMDA, contribue à la fois à l'expression des symptômes et à la progression de la maladie dans la démence neurodégénérative.

La mémantine est un antagoniste voltage-dépendant non compétitif des récepteurs NMDA d'affinité modérée. Elle module les effets de taux élevés pathologiques de glutamate qui pourraient aboutir à un dysfonctionnement neuronal.

Études cliniques :

Une étude pivot en monothérapie dans une population de patients atteints de maladie d'Alzheimer au stade modéré à sévère (score total du Mini Mental Test [MMSE] de 3 à 14 au début de l'étude) a inclus un total de 252 patients ambulatoires. L'étude a montré le bénéfice du traitement par la mémantine par rapport au placebo à 6 mois (analyse des cas observés pour la Clinician's Interview Based Impression of Change [CIBIC-plus] : p = 0,025 ; l'Alzheimer's Disease Cooperative Study-Activities of Daily Living [ADCS-ADLsev] : p = 0,003 ; la Severe Impairment Battery [SIB] : p = 0,002).

Une étude pivot en monothérapie dans le traitement de la maladie d'Alzheimer au stade léger à modéré (score total MMSE de 10 à 22 au début de l'étude) a inclus un total de 403 patients. Les patients traités par la mémantine ont présenté, de façon statistiquement significative, un effet supérieur à celui observé chez les patients sous placebo sur les critères primaires : Alzheimer's Disease Assessment Scale ([ADAS-cog] ; p = 0,003) et CIBIC-plus (p = 0,004) à la semaine 24 (LOCF - dernière observation reportée). Dans une autre étude de monothérapie dans la maladie d'Alzheimer au stade léger à modéré, un total de 470 patients (score total MMSE de 11 à 23 au début de l'étude) ont été randomisés. L'analyse primaire définie de façon prospective n'a pas permis de conclure à une différence statistiquement significative sur le critère primaire d'efficacité à la semaine 24.

Une méta-analyse des patients atteints d'une maladie d'Alzheimer au stade modéré à sévère (score total MMSE < 20) issue de 6 études de phase III versus placebo sur une durée de 6 mois (incluant les études en monothérapie et les études chez des patients traités par inhibiteurs de l'acétylcholinestérase à posologie stable) a montré un effet statistiquement significatif en faveur de la mémantine pour les domaines cognitif, global et fonctionnel. Chez les patients pour lesquels une aggravation concomitante sur les trois domaines était identifiée, les résultats ont montré un effet statistiquement significatif de la mémantine sur la prévention de l'aggravation, puisque 2 fois plus de patients sous placebo ont montré une aggravation dans les trois domaines par rapport à ceux traités par la mémantine (21 % vs 11 %, p < 0,0001).

PP PHARMACOCINÉTIQUE

Absorption :

La mémantine présente une biodisponibilité absolue d'environ 100 %. Le Tmax se situe entre 3 et 8 heures. Rien n'indique que la prise de nourriture influe sur l'absorption de mémantine.

Distribution :

Des doses quotidiennes de 20 mg aboutissent à des concentrations plasmatiques de mémantine à l'état d'équilibre comprises entre 70 et 150 ng/ml (0,5 à 1 µmol) avec d'importantes variations interindividuelles. Avec des doses quotidiennes de 5 à 30 mg, on a calculé un rapport moyen de 0,52 entre le liquide céphalorachidien (LCR) et le sérum. Le volume de distribution se situe autour de 10 l/kg. Environ 45 % de la mémantine est liée aux protéines plasmatiques.

Métabolisme :

Chez l'homme, environ 80 % de la dose est présente sous forme inchangée. Les principaux métabolites chez l'homme sont le N-3,5-diméthyl-gludantan, le mélange isomère de 4- et 6-hydroxy-mémantine et le 1-nitroso-3,5-diméthyl-adamantane. Aucun de ces métabolites ne présente d'activité antagoniste NMDA. Aucun métabolisme catalysé par le cytochrome P450 n'a été détecté in vitro.

Dans une étude avec administration par voie orale de ^{14}C-mémantine, 84 % de la dose, en moyenne, a été retrouvée dans les 20 jours, dont plus de 99 % par excrétion rénale.

Élimination :

La mémantine est éliminée de manière mono-exponentielle avec un $t_{½}$ terminal de 60 à 100 heures. Chez les volontaires présentant une fonction rénale normale, la clairance totale (Cl_{tot}) s'élève à 170 ml/min/1,73 m^2, et une partie de la clairance rénale totale se fait par sécrétion tubulaire.

La clairance rénale fait également intervenir une réabsorption tubulaire, probablement par l'intermédiaire des protéines assurant le transport des cations. Le taux d'élimination rénale de la mémantine dans des urines alcalines peut être réduit d'un facteur de 7 à 9 (cf Mises en garde/Précautions d'emploi).

L'alcalinisation de l'urine peut résulter de modifications radicales du régime alimentaire, par exemple du passage d'un régime carné à un régime végétarien, ou de l'ingestion massive de tampons gastriques alcalinisants.

Linéarité :

Les études chez des volontaires ont montré une pharmacocinétique linéaire dans l'intervalle de dose allant de 10 à 40 mg.

Relation pharmacocinétique/pharmacodynamie :

Avec une dose de mémantine de 20 mg par jour, les taux dans le LCR correspondent à la valeur k_i (k_i = constante d'inhibition) de la mémantine, soit 0,5 µmol dans le cortex frontal humain.

PP SÉCURITE PRÉCLINIQUE

Les études à court terme chez le rat ont montré que la mémantine, comme les autres antagonistes NMDA, induisait une vacuolisation neuronale et une nécrose (lésions d'Olney) uniquement à des doses aboutissant à de très fortes concentrations sériques. Une ataxie et d'autres signes précliniques ont précédé la vacuolisation et la nécrose. Étant donné que ces effets n'ont pas été observés dans des études au long cours, ni chez les rongeurs ni chez les non-rongeurs, la pertinence clinique de ces observations est inconnue.

Des modifications oculaires ont été observées de manière inconstante lors d'études de toxicité à doses répétées chez les rongeurs et le chien, mais pas chez le singe. Les examens ophtalmologiques spécifiques réalisés durant les études cliniques de la mémantine n'ont révélé aucune modification oculaire.

Une phospholipidose dans les macrophages pulmonaires causée par l'accumulation de mémantine dans les lysosomes a été observée chez les rongeurs. Cet effet est connu pour d'autres substances actives dotées de propriétés amphiphiliques cationiques. Il existe peut-être un lien entre cette accumulation et la vacuolisation observée dans les poumons. Cet effet a uniquement été observé à de fortes doses chez les rongeurs. La pertinence clinique de ces observations est inconnue.

Aucune génotoxicité n'a été observée suite aux études standard de la mémantine. Aucun effet carcinogène n'a été observé lors d'études vie entière chez la souris et le rat. La mémantine ne s'est pas avérée tératogène chez le rat et le lapin, même à des doses maternotoxiques, et aucun effet indésirable de la mémantine sur la fertilité n'a été relevé. Chez le rat, un retard de croissance du foetus a été constaté à des niveaux d'exposition identiques ou légèrement supérieurs à ceux utilisés chez l'homme.

DP MODALITÉS DE CONSERVATION

Comprimés pelliculés :

Durée de conservation :
4 ans.
Pas de précautions particulières de conservation.

Solution buvable :

Durée de conservation :
4 ans.
A conserver à une température ne dépassant pas 30 °C.
Conserver et transporter le flacon avec la pompe fixée uniquement en position verticale.
Après ouverture :
Utiliser le contenu du flacon sous 3 mois.

DP MODALITÉS MANIPULATION/ÉLIMINATION

Avant la première utilisation, la pompe doseuse doit être vissée sur le flacon. Pour enlever le bouchon à vis du flacon, celui-ci doit être tourné dans le sens inverse des aiguilles d'une montre et dévissé complètement (figure 1).

Mise en place de la pompe doseuse sur le flacon :

La pompe doseuse doit être retirée de son emballage plastique (figure 2) et insérée dans la partie haute du flacon, en glissant avec précaution le tube en plastique creux dans le flacon. Ensuite, maintenir la pompe doseuse avec l'encolure du flacon et visser la pompe dans le sens des aiguilles d'une montre jusqu'à ce qu'elle soit fermement fixée (figure 3). Pour son emploi, la pompe doseuse est vissée une fois seulement en début d'utilisation et ne doit jamais être dévissée.

Utilisation de la pompe doseuse pour administration :

La tête de la pompe doseuse a deux positions et est facile à tourner soit dans le sens inverse des aiguilles d'une montre (position déverrouillée) soit dans le sens des aiguilles d'une montre (position verrouillée). En position verrouillée, la tête de la pompe doseuse ne peut être actionnée vers le bas. La solution ne peut être délivrée qu'en position déverrouillée. Pour ce faire, tourner la tête de la pompe doseuse dans le sens de la flèche approximativement d'un huitième de tour, jusqu'à sentir une résistance (figure 4). La pompe doseuse est ainsi prête à l'emploi.

Préparation de la pompe doseuse :

Quand elle est actionnée pour la première fois, la pompe doseuse ne délivre pas la quantité correcte de solution buvable. De ce fait, la pompe doit être préparée (amorcée) en actionnant complètement la tête de la pompe doseuse vers le bas cinq fois de suite (figure 5). La solution ainsi délivrée doit être jetée. A la prochaine pression de la tête de la pompe doseuse vers le bas et de façon complète, la dose adéquate est dispensée (1 dose unitaire/pression est équivalente à 0,5 ml de solution buvable et contient environ 5 mg de chlorhydrate de mémantine, la substance active) : figure 6.

Utilisation correcte de la pompe doseuse :

Placer le flacon sur une surface plane et horizontale, par exemple sur une table, et ne l'utiliser qu'en position verticale. Tenir un verre contenant un peu d'eau ou une cuillère sous le bec verseur et presser la tête de la pompe doseuse d'un mouvement ferme, délicat et continu (pas trop lentement) vers le bas jusqu'à la butée (figures 7 et 8).
La tête de la pompe doseuse peut alors être relâchée et la pompe est prête pour la pression suivante.
La pompe doseuse ne peut être utilisée qu'avec la solution de chlorhydrate de mémantine contenue dans le flacon fourni et pas avec d'autres substances ou d'autres flacons. Si la pompe ne fonctionne pas

comme décrit au cours de son utilisation ni en suivant les instructions, le patient doit consulter son médecin traitant ou un pharmacien. La pompe doseuse doit être verrouillée après utilisation.

PRESCRIPTION/DÉLIVRANCE/PRISE EN CHARGE

LISTE I
Surveillance particulière nécessaire pendant le traitement.
Prescription initiale annuelle réservée aux médecins spécialistes en neurologie, en psychiatrie, aux médecins spécialistes titulaires du diplôme d'études spécialisées complémentaires de gériatrie et aux médecins spécialistes ou qualifiés en médecine générale titulaires de la capacité de gérontologie.

AMM EU/1/02/219/008 ; CIP 34009**3595531**9 (2002, RCP rév 20.11.2009) 56 cp à 10 mg.
EU/1/02/219/015 ; CIP 34009**5707796**9 (2006, RCP rév 20.11.2009) 98 × 1 cp à 10 mg.
EU/1/02/219/024 ; CIP 34009**3874030**1 (2008, RCP rév 20.11.2009) 28 cp à 20 mg.
EU/1/02/219/046 ; CIP 34009**5737053**4 (2008, RCP rév 20.11.2009) 98 × 1 cp à 20 mg.
EU/1/02/219/005 ; CIP 34009**3595560**9 (2002, RCP rév 20.11.2009) sol buv.

Prix : 88.80 euros (56 comprimés à 10 mg).
88.80 euros (28 comprimés à 20 mg).
79.84 euros (flacon de 50 g).
Remb Séc soc à 65 %. Collect.
Modèle hospitalier : Collect.

Titulaire de l'AMM : H. Lundbeck A/S, Ottiliavej 9, DK-2500 Valby, Danemark.
LUNDBECK SAS - 37-45, quai du Président-Roosevelt - 92445 Issy-les-Moulineaux cdx
Tél : 01 79 41 29 00 - Info médic et Pharmacovigilance : Tél : 01 79 41 29 79

DOSSIER MEDICAMENT EXPERIMENTAL
PLACEBO LACTOSE

PREVENTION DU DEVELOPPEMENT
DE DOULEUR NEUROPATHIQUE
POST-MASTECTOMIE POUR TUMORECTOMIE AVEC OU SANS CURAGE PAR L'ADMINISTRATION DE MEMANTINE EN PRE ET POST-CHIRURGIE

Dossier du Médicament expérimental
PLACEBO LACTOSE GELULES

Date : Octobre 2011

1. Description et composition

Le placebo est uniquement composé de lactose selon les informations du tableau ci-après :

Nom du composant	Quantité centésimale	Fonction	Référence aux normes
Lactose	500 mg *<br>(gélule taille 0 : volume 0.68 ml)	Excipient	P.E 7ème éd.

* densité tassée du lactose en g/ml x 0.68

2. Développement pharmaceutique

L'essai clinique est réalisé en ouvert, le placebo est présenté comme du lactose couramment utilisé comme excipient à effet notoire.
L'apparence des traitements à l'étude est donc différente et décrite dans le tableau ci-après :

Traitement	Forme pharmaceutique	Apparence
Lactose	gélules	Taille 0, couleur bleue
Ebixa®<br>10 mg et 20 mg	comprimés pelliculés	10 mg : ovale, avec une ligne de sécabilité ; portant l'inscription « 10 » sur une face et « MM » sur l'autre face ; jaune pâle à jaune<br>20 mg : oblong, ovale, portant l'inscription « 20 » sur une face et l'inscription « MEM » sur l'autre ; rouge pâle à gris-rouge

3. Fabrication

3.1. Fabricant

La fabrication sera réalisée par la pharmacie du CHU de Clermont-Ferrand.

PHARMACIE - CHU CLERMONT-FERRAND
58 Rue de Montalembert - BP 69
63 003 CLERMONT-FERRAND Cedex 1

Chef de service : Pr Jean CHOPINEAU

Autorisation n°477 délivrée par la Préfecture du Puy de Dôme par arrêté portant autorisation de Pharmacie à Usage Intérieur intégrant certaines activités optionnelles.

3.2. Formule de fabrication

La quantité totale de gélules nécessaires pour cet essai est de 1120 gélules et 28 pour l'échantillothèque. La taille de lot maximale est de 300 gélules.
3 lots de 300 gélules et un lot de 248 gélules seront donc fabriqués.

La formule de fabrication pour 1 lot de 300 gélules est indiquée dans le tableau suivant.

Nom du composant	Quantité centésimale	Quantité pour 1 lot de 300 gélules	Fonction
Lactose	500 mg	150 g	Excipient

La formule de fabrication pour 1 lot de 248 gélules est indiquée dans le tableau suivant.

Nom du composant	Quantité centésimale	Quantité pour 1 lot de 248 gélules	Fonction
Lactose	500 mg	124 g	Excipient

3.3. Description du procédé de fabrication et de contrôle qualité

Etape	Matériel utilisé	Contrôles en cours	Description
1 : Pesée du lactose	Balance, bécher	Impression ticket pesée<br>Contrôle identité matière première	
2 : Remplissage et fermeture des gélules	Gélulier taille 0	Examen visuel après fermeture des gélules<br>Uniformité de masse	
3 : Mise sous blister	Système Medidose®	Contrôle des mentions de l'étiquetage	

3.4. Contrôle des étapes critiques et intermédiaires
Non applicable.

3.5. Validation et / ou évaluation du procédé
Non applicable.

4. Contrôle des excipients

4.1. Spécifications
Le lactose a une monographie de contrôle à la Pharmacopée européenne 7ème édition.

4.2. Procédures analytiques
Non applicable.

4.3. Validation des procédures analytiques
Non applicable.

4.4. Justification des spécifications
Non applicable.

4.5. Excipients d'origine humaine ou animale
Non applicable.

4.6. Nouvel excipient
Non applicable.

5. Contrôle du placebo

5.1. Spécifications
Spécifications pour la libération et la stabilité du placebo et méthode

**Spécifications**	**Critères**
**Caractères organoleptiques :**	
• aspect	Gélule taille 0
• couleur	Bleu
**Essais** :	
• Uniformité de masse	selon Pharmacopée européenne 7ème éd.

5.2. Procédures analytiques
Description des méthodes analytiques pour tous les tests inclus dans les spécifications.

5.3. Conditionnement
Description du conditionnement primaire. *Blister PVC/ Alu*

5.4. Stabilité
La durée de conservation du lactose étant de 2 ans et demi, une durée de conservation de 1 an été attribuée pour le placébo. En effet la mise en gélule du lactose n'entraine pas de modification des caractéristiques physiques de cet excipient ni de risque de dégradation.

ANNEXES

1 Installation et équipement
Non applicable.

2 Évaluation de l'innocuité des agents adventifs
Non applicable.

3 Nouveaux excipients
Non applicable.

4 Solvants pour reconstitution ou dilution
Non applicable.

25. ANNEXE 6 AUTORISATION DE FABRICATION D'UNITES THERAPEUTIQUE PAR LA PUI DU CHU DE CLERMONT-FERRAND

Liberté • Égalité • Fraternité
RÉPUBLIQUE FRANÇAISE

PREFECTURE DU PUY-DE-DÔME

ARRETE

LE PRÉFET DE LA RÉGION AUVERGNE
PRÉFET DU PUY-DE-DÔME
Chevalier de la Légion d'Honneur
Chevalier de l'Ordre National du Mérite

PORTANT AUTORISATION DE PHARMACIE À USAGE INTÉRIEUR
INTÉGRANT CERTAINES ACTIVITÉS OPTIONNELLES

AUTORISATION N° 477

VU le Code de la Santé Publique, notamment les articles L 5126.5, L 5126.7, L 6111.1, R 5104.9, R 5104.15 et R 5104.25 ;

VU la demande présentée par Monsieur le Directeur Général du Centre Hospitalier Universitaire de Clermont-Ferrand, en vue d'obtenir une autorisation de pharmacie à usage intérieur intégrant les activités optionnelles suivantes :

- stérilisation des dispositifs médicaux
- réalisation des préparations hospitalières
- réalisation des préparations pour essais cliniques

VU l'avis du Conseil Central de la section D de l'Ordre des Pharmaciens en date des 12 et 24 décembre 2002 ;

VU l'avis de Monsieur le Directeur Régional des Affaires Sanitaires et Sociales en date du 22 janvier 2003 ;

VU les arrêtés des 2 mai 1973, 25 août 1978 et 23 février 1993 portant création et modification de la licence n° 278 autorisant l'ouverture d'une pharmacie à usage intérieur au Centre Hospitalier Universitaire de Clermont-Ferrand ;

Sur proposition de Monsieur le Secrétaire Général de la Préfecture du Puy de Dôme ;

ARRETE

Article 1 - L'autorisation prévue à l'article L 5126.7 du Code de la Santé Publique est accordée à Monsieur le Directeur Général du Centre Hospitalier Universitaire de Clermont-Ferrand.

.../...

63033 CLERMONT-FERRAND CEDEX [illegible]
TEL. 04 73 98 63 63 - TELEX 990 264 F - FAX 04 73 98 61 00
http://www.auvergne.pref.gouv.fr

2

Article 2 - En application des dispositions prévues à l'article R 5104.15 du Code de la Santé Publique, cette autorisation intègre également les activités optionnelles suivantes :

- stérilisation des dispositifs médicaux
- réalisation des préparations hospitalières
- réalisation des préparations pour essais cliniques.

Article 3 - Les locaux concernés par ces activités sont situés sur les deux sites géographiques où sont implantées les pharmacies à usage intérieur, mais en des lieux différents, selon le descriptif présenté dans le dossier de demande.

Article 4 - Les activités concernées par l'autorisation doivent être réalisées en conformité avec les dispositions de l'arrêté du 22 juin 2001 relatif aux bonnes pratiques de pharmacie hospitalière.

Article 5 - Toute infraction aux dispositions de l'arrêté du 22 juin 2001 peut entraîner la suspension ou le retrait de tout ou partie de la présente autorisation.

Article 6 - La présente autorisation est enregistrée sous le numéro 477. Elle annule et remplace l'autorisation n° 276 accordée le 23 février 1993.

Article 7 - Le Secrétaire Général de la Préfecture du Puy de Dôme et le Directeur Régional des Affaires Sanitaires et Sociales d'Auvergne, sont chargés, chacun en ce qui le concerne, de l'exécution du présent arrêté.

Fait à Clermont-Ferrand, le 15 FEV. 2003

LE PRÉFET DE LA REGION AUVERGNE
PREFET DU PUY-DE-DOME

P/Le Préfet, et par délégation
Le Secrétaire Général,

Henri d'ABZAC

26.ANNEXE 7 CURRICULUM VITAE

CURRICULUM VITAE

Gisèle PICKERING
Née le 30 mai 1958 à Chamalières (63)
N° inscription Ordre des médecins 63 4083 N° ADELI 63 10 4083 9 RPPS : 10003167193
Email : gisele.pickering@u-clermont1.fr Tél :04 73 17 84 16

ACTIVITES PROFESSIONNELLES

2004-	Maître de Conférences des Universités/ Praticien Hospitalier. Service de Pharmacologie, Faculté de Médecine INSERM U766/ INSERM CIC501 CHU de Clermont-Ferrand
1999-2004	Assistante universitaire / Praticien attaché,
1996-99	Praticien attaché,
1991-93	Assistante Universitaire University College, Cork, Irlande
1984-85	Fellowship University College Cork, Irlande

TITRES UNIVERSITAIRES

2006	Habilitation à Diriger des Recherches
1997	Doctorat d'Université
1990	Doctorat de Médecine
1984	Diplôme d'Etat de Docteur en Pharmacie
2003	Module de Méthodologie statistique.
2001	Certificat de Maîtrise de Sciences Biologiques et Médicales
1997	Capacité de Médecine et Biologie du Sport
1983	DESS - Certificat d'Aptitude à l'Administration des Entreprises

AUTRES ACTIVITES

- Participation à des sociétés savantes : Société Française d'Evaluation et Traitement de la Douleur, International Association for the Study of Pain, Société des Neurosciences, Société Française de Pharmacologie,
- Participation à des actions ministérielles de santé publique (Migrel®, Mobiqual®).
- Membre du Comité technique du CIC, Clermont Ferrand.
- Membre du Comité scientifique de plusieurs revues scientifiques.
- Membre de l'Institut Upsa de la douleur.
- Reviewer de publications internationales.
- Membre du Comité international de pilotage du groupe « Douleur et personne âgée» créé en 2005 au IASP.
- Membre du Collectif français Doloplus®.

-Depuis 2005 :

- Investigateur principal et financement de 15 protocoles de recherche clinique
- 17 publications internationales avec comité de lecture, 20 publications sollicitées, 5 chapitres dans ouvrages, 10 conférences internationales invitées, 30 conférences nationales ; organisatrice de cours supérieurs et séminaires, présentation de posters au cours de congrès en Douleur et en Pharmacologie

- Coordinateur d'ouvrage : Douleur de la personne âgée, 2010, de numéros spéciaux : Journal of pain Management, Douleur et analgésie (2007 et 2011)
- Encadrement d'étudiants (thèse, M2, M1)

13/01/11

Claude DUBRAY
Né le 20 juin 1951 à Chamalières (Puy de Dôme)

N° inscription à l'Ordre des Médecins : 63/4062
N° ADELI : 631040623
N° RPPS : 10003169801

TITRES UNIVERSITAIRES

- Doctorat d'Etat en Médecine - 1982
- Certificat d'Etudes Spéciales de Pédiatrie et de Puériculture - 1982
- Diplôme d'Etudes et de Recherches en Biologie Humaine (DERBH) - 1985
- Doctorat d'Université (Décret 1984) en Pharmacologie - 1992.
- Habilitation à Diriger des Recherches - 1994

FONCTIONS UNIVERSITAIRES - Faculté de Médecine de Clermont Ferrand

- Chef de Clinique des Universités - 1983-1984
- Maître de Conférences des Universités - 1993-1997
- Professeur des Universités depuis septembre 1997

FONCTIONS HOSPITALIERES - C.H.U. de Clermont Ferrand

- Interne des Hôpitaux de 1978 à 1981
- Médecin Attaché des Hôpitaux de 1982 à 1983
- Assistant des Hôpitaux de 1983 à 1984
- Praticien Hospitalier depuis 1993 Service de Pharmacologie Clinique et Toxicologie
- Chef de Service Pharmacologie Clinique CHU de Clermont-Ferrand

AUTRES FONCTIONS

I.N.S.E.R.M.
- Médecin coordonnateur du CIC-501 depui janv 2005

INDUSTRIE PHARMACEUTIQUE (Centre de Rech. des Laboratoires SANDOZ France)
- Responsable du Service de Pharmacologie Clin. et Exp. - 1984-1991
- Directeur Adjoint du Centre de Recherches - SANDOZ France - 1989-1993
- Responsable du Département de Pharmacologie Humaine (regroupant les services de Pharmacologie Clinique, Pharmacologie Expérimentale et Pharmacocinétique) 1991-1993

PRESIDENT DE LA DRCI (Délégation Régionale à la Recherche et à l'Innovation) Région Auvergne, depuis Octobre 1999
Membre du bureau executif de la Délégation Inter-régionale à la recherche clinique Rhône-Alpes-Auvergne
VICE-PRESIDENT du DIRECTOIRE du CHU de Clermont-Ferrand en charge de la Recherche

SOCIETES SAVANTES
- Membre de la Société Française de Pharmacologie
- Membre de l'Association pour le Développement de la Pharmacologie Clinique
- Membre de l'International Association for the Study of Pain (IASP)
- Membre de la Société D'étude et de Traitement de la Douleur (SETD) (Trésorier 1995-2001
- Membre de la Société des Neurosciences

COMITE DE REDACTION DE REVUE
La Lettre du Pharmacologue
Reviewer dans une dizaine de revues à comité de lecture international

06/01/2011

Curriculum vitae

Personal

Name: **Xavier Durando, MD, PhD**
Date of birth : 09.04.1970
Nationality : French
Address : Centre Jean Perrin
58, rue Montalembert - B.P. 392, 63011 Clermont-Ferrand, Cedex 01, France
Phone : +33 473 278 000
E-mail xavier.durando@cjp.fr
N ° council of the National Order 63/4763

Education and medical training

1981-1988	Jeanne D'Arc College, Clermont Ferrand
1988-1996	Faculty of Medicine, Clermont Ferrand

Degree & Titles

1988	High School diploma
1988	National Olympiads of the chemistry
1991	Master n°1 Biology and medical Sciences<br>- Statistical, computer, and modeling<br>- Methods in clinical and epidemiological research
1992	Master n°2 Biology and medical Sciences<br>- General Immunology, immune-pathology and physiological mechanisms<br>- Biophysics of radiation and imaging
2000	MD thesis
2000-2002	Appointment as head assistant clinician and clinical instructor
2002	Appointment as head senior clinician in charge of the neuro-oncology and melanoma outpatient clinic
2008	PhD thesis
2009	HDR (French post-doctoral degree allowing its holder to supervise PhD students)

Teaching experience

2000-	Medical oncology training at the University of medicine, Clermont Ferrand
2002-	General oncology training, School of nurse of Clermont Ferrand
2002-2004	MD thesis co-supervision Long-term survival in advanced melanomas
2000-2003	PhD thesis co-supervision. Potentiation of nitrosourea
2004-2007	PhD thesis co supervision. Potentiation of melanoma treatment
2004-2006	MD thesis co-supervision. Retrospective analyze of testis tumors treatment
2006-	General oncology training, School of preparer pharmacy
2007	Master supervision. Medical communication

Research Activities

1997-2000	Clinical research for MD thesis. High-dose BCNU followed by autologous hematopoietic stem cell transplantation in supratentorial high-grade malignant gliomas.
2000-	Clinical research, phase I,II,III
2000-	Methionine dependency and cancer treatment
2000-	Potentiation strategies for treatment glioma and melanoma.

Current Positions

2002-	Head clinician in charge of the neuro-oncology and melanoma outpatient clinic of the Comprehensive Cancer Centre Jean Perrin, France
2010-	Co-chairman of the Department of Medical Oncology Jean Perrin Center

Society Memberships

2006	A.R.T.B.C.	Internationale, Chronotherapy Group
2008	ANOCEF	French communitie of neuro-oncology
2008	AACR, ASCO	Member

01 12 2010

1

21/06/2011.

MOURET-REYNIER Marie-Ange

Ordre National des Médecins : n° 63 04935

Née le 20/08/1971 à Chamalières (63)
Adresse : 1 Boulevard Bazin
63400 Chamalières
☎ 04 73 35 81 68

CURSUS PROFESSIONNEL

Externat :

Faculté de Médecine, Clermont-Fd

Internat (concours juin 97) :

- nov 97 à avril 98 : Dr Pauchard, Dr Bichoffe, Médecine Interne et Cancérologie, CHG Montluçon (Allier).
- mai 98 à oct 98 : Pr Verrelle, Radiothérapie, Centre J. Perrin, Clermont-Fd.
-
- nov 98 à avril 99 : Pr Plagne, Oncologie Médicale, Service d'Hospitalisation, Centre J. Perrin, Clermont-Fd.
- mai 99 à oct 99 : Pr Plagne, Oncologie Médicale, Service d'Hospitalisation, Centre J. Perrin, Clermont-Fd.
- nov 99 à avril 00 : Pr Chollet, Oncologie Médicale, Hôpital de Jour, Centre J. Perrin, Clermont-Fd.
- mai 00 à oct 00 : Pr Chollet, Oncologie Médicale, Hôpital de Jour, Centre J. Perrin, Clermont-Fd.
- nov 00 à avril 01 : Pr Chollet, Oncologie Médicale, Hôpital de Jour, Centre J. Perrin, Clermont-Fd.

TITRES

✓ ***Thèse de Docteur en Médecine*** soutenue le 8 octobre 2001 : Chimiothérapie d'induction des cancers du sein opérables : expérience générale du Centre Jean Perrin ; étude des protocoles FEC 100 et NET.

✓ ***Qualification D.E.S d'oncologie médicale*** le 06 octobre 2002

✓ Inscription n° 63 04935 au tableau de l'Ordre National des Médecins depuis le 20 décembre 2001

VILLATTE Christine
Née le 10 Janvier 1966 à Limoges (Haute-Vienne)

Adresse : 8 Rue des Moulins
Le Lot
63970 AYDAT
☎ : 04-73-79-31-30 / 06-09-13-31-46

Adresse professionnelle : Centre Régionale de Lutte Contre le Cancer Jean Perrin
58 rue Montalembert BP.392
63011 CLERMONT-FERRAND CEDEX
☎ : 04-73-27-80-80 (standard) ou 81-00 (consultations), poste 8876

N° d'inscription au conseil de l'Ordre : 63/4597
N° ADELI : 631045978
N° CPS : 2300149973

Etudes :

- 1983 : Baccalauréat Série D
- 1983 à 1995 : études de Médecine à la Faculté de Médecine de Limoges

Titres et diplômes :

- 1998 : Doctorat en médecine, Faculté de Médecine de Limoges
- 2002 : Capacité « Evaluation et traitement de la douleur », Faculté de Médecine Saint-Antoine, Université Paris VI (formation de Septembre 2000 à Juin 2002)
- 2009 : Diplôme d'Hypno-analgésie, Institut Français d'Hypnose (IFH) à Paris (formation d'Octobre 2007 à Juin 2009)

Cursus pendant le Résidanat (TCEM):

- de Novembre 1993 à Avril 1994 : résidente dans le service de Cancérologie (Docteur Leduc) au CHG de Brive
- de mai 1994 à Octobre 1994 : résidente dans le service de Psychiatrie (Docteur Gény) au CHG de Brive
- de Novembre 1994 à Avril 1995 : résidente dans le service de Cancérologie (Docteur Leduc) au CHG de Brive
- de Mai 1995 à Octobre 1995 : résidente dans le service des Urgences (Professeur Piva) au CHRU de Limoges

Expériences et activités professionnelles :

- de Novembre 1995 à Janvier 1996 : Faisant Fonction d'Interne (FFI) dans le service de Médecine Interne-Rhumatologie (Docteur Lambert de Cursay) au CHG de Brive
- de Novembre 1995 à Juillet 1996 : remplacements en Médecine Générale
- de Mars 1996 à Décembre 1999 : attachée en médecine au Centre de Lutte Contre le Cancer Jean Perrin à Clermont-ferrand (Mars à Avril 1996 : à l'hôpital de jour, Mai 1996 à Juin 1997 : dans le service d'hospitalisation d'oncologie médicale, Juin 1997 à Février 1999 : à l'hôpital de jour)

- à partir de Février 1999 : activité avec 2 temps partiel sur 2 établissements ayant des activités complémentaires :

  . de Février 1999 à Novembre 2009, médecin mi-temps au Centre Médical Les Sapins à Ceyrat, établissement de Soins de Suite et Réadaptation à orientation cancérologie : soins de support et soins palliatifs (avec des lits identifiés « palliatifs »). Activité réduite à 40% à partir de Mai 2008, Fin de l'activité début Novembre 2009,

  . de Novembre 2009 à Mars 2011, médecin temps partiel (3 demi-journées/semaine) à CliniDom, structure d'HAD (Hospitalisation à Domicile),

  . et médecin au CLCC Jean Perrin :

  - 5 vacations/semaine jusqu'en Décembre 2000 : à l'hôpital de jour de Février 1999 à Décembre 1999, dans le service d'hospitalisation de Radiothérapie de Juin 2000 à Décembre 2000,
  - CDI de Médecin Généraliste de Centre de Lutte Contre le Cancer mi-temps (dans le service d'hospitalisation de Radiothérapie de Janvier 2001 à Mars 2006, dans le service d'hospitalisation d'oncologie médicale de Avril 2006 à Mars 2007),
  - et **médecin « Référent Douleur »** (activité consacrée à la prise en charge de la douleur dans l'établissement) : mi-temps d'Avril 2007à Mars 2011,

- et à partir d'Avril 2011, activité exclusivement au CLCC Jean Perrin : **médecin douleur**, à temps partiel de 80%.

Le 13 OCTOBRE 2011

Docteur Christine VILLATTE

DOCTEUR VILLATTE CHRISTINE
MEDECIN DOULEUR
Evaluation et traitement de la douleur
CENTRE JEAN PERRIN
Tél : 04-73-27-84-77
Fax : 04-73-27-84.78
N° Adeli 631045978

27.ANNEXE 8- FORMULAIRE DE RECUEIL D'EVENEMENT INDESIRABLE GRAVE

A FAXER INPERATIVEMENT DANS LES 24 H AU PROMOTEUR AU 04.73.17.84.12

Date de déclaration : I__I__I I__I__I I__I__I__I__I

1. IDENTIFICATION DE LA RECHERCHE BIOMEDICALE

N° EudraCT : 2011-004030-33
Code Protocole attribué par le promoteur : RBHP 2011 PICKERING 3

Titre de l'essai clinique :
PREVENTION DU DEVELOPPEMENT DE DOULEUR NEUROPATHIQUE POST-MASTECTOMIE / TUMORECTOMIE PAR L'ADMINISTRATION DE MEMANTINE EN PRE ET POST-CHIRURGIE

2. INFORMATIONS SUR LE SUJET

Identification du sujet :

Nom (initiales) : I__I__I__I

Prénom : I__I__I

N° Sujet (CRF) : I__I__I__I__I__I

Date de naissance : I__I__I I__I__I I__I__I__I__I

Age : I__I__I__I

Poids (kg) : I__I__I__I

Taille (cm) : I__I__I__I

Sexe : F ☐ M ☐

Antécédents médicaux et familiaux pertinents :

3. INFORMATIONS SUR LE(S) MEDICAMENT(S)

Nom commercial ou DCI	Dosage	N° de lot	Voie d'adm.	Posologie (Dose / rythme)	Indication thérapeutique	Début de traitement (date et heure)	Fin de traitement (date et heure (✱)	Code levé ?
1								Oui ☐<br>Non ☐
2								Oui ☐<br>Non ☐
3								Oui ☐<br>Non ☐

✱ Noter « continue » si le traitement n'est pas arrêté.

Si levée de l'insu, résultat : ____________________________
Disparition de l'événement après arrêt du traitement ? Oui ☐ Non ☐

4. EVALUATION DU LIEN DE CAUSALITE

Selon le promoteur, l'événement semble plutôt lié :

Au(x) traitement(s) à l'essai ☐
Au(x) traitement(s) associés ☐
A une maladie intercurrente ☐
Au(x) procédure(s) de l'essai ☐
Autre, à préciser : ☐
____

Commentaires pertinents :
____
____

Selon l'investigateur, l'événement semble plutôt lié :

Au(x) traitement(s) à l'essai ☐
Au(x) traitement(s) associés ☐
A une maladie intercurrente ☐
Au(x) procédure(s) de l'essai ☐
Autre, à préciser : ☐
____

Commentaires pertinents :
____
____

5. INFORMATIONS SUR LES TRAITEMENTS ASSOCIES MEDICAMENTEUX OU NON (à l'exclusion de ceux utilisés pour traiter l'événement)

Nom commercial ou DCI	Dosage	N° de lot	Voie d'adm.	Posologie (Dose / rythme)	Indication thérapeutique	Début de traitement (date, heure)	Fin de traitement (date, heure)
4							
5							
6							
7							

6. INFORMATIONS SUR L'EVENEMENT INDESIRABLE GRAVE

☐ Décès
☐ Mise en jeu du pronostic vital
☐ Invalidité ou incapacité
☐ Hospitalisation ou prolongation d'hospitalisation
Date de début : I__I__I I__I__I I__I__I
Date de fin : I__I__I I__I__I I__I__I
☐ Anomalie congénitale

Lieu de survenue : ______________________
Date de survenue : I__I__I I__I__I I__I__I
Heure de survenue : I__I__I I__I__I

☐ Autre (préciser) :___________________________

Description de l'événement indésirable - Préciser les symptômes prédominants, la chronologie, éventuellement le diagnostic et les traitements de l'événement (joindre les comptes-rendus anonymisés d'hospitalisation d'examens et/ou résultats de laboratoire) :

____
____
____
____
____
____

Evolution : ☐ Amélioration ☐ Stabilité ☐ Aggravation ☐Survie avec séquelles
☐ Décès (cause : lié à l'événement ☐Oui ☐Non) ☐Evolution inconnue

Description (joindre les comptes-rendus anonymisés d'hospitalisation d'examens et/ou résultats de laboratoire) :
____
____
____

Un ou des produits ont-ils été réintroduits ? Réapparition de l'événement après réintroduction ?

Oui		N° N° N°		Non		Oui		N° N° N°		Non	

Si oui, date : I__I__I I__I__I I__I__I heure : I__I__I I__I__I

DIAGNOSTIC DIFFERENTIEL :
Autres étiologies envisagées:
____
____

Examens complémentaires réalisés et résultats :
____
____

7. INFORMATIONS SUR LE DECLARANT

Nom et adresse du centre investigateur :
____
____

Centre n° : ______________ Investigateur : ______________________________
Tél. : ____________________ Email : ______________________@______________________
Service : ________________

Nom et qualité du déclarant : ____________________________ Signature :

8. INFORMATIONS SUR LE PROMOTEUR (*cadre réservé au promoteur, ne pas remplir*)

Nom et adresse du promoteur :

____

____

Date de réception par le promoteur : I__I__I I__I__I I__I__I Type de rapport : ☐ initial

☐ suivi n°_________

Date de déclaration aux autorités : I__I__I I__I__I I__I__I

N° d'identification de l'événement par le promoteur : ______________________

Identification de l'autorisation de recherche : ______________________

Nom et qualité du représentant du promoteur:

____

Tél. : __________________ Email : ___________________@________________________

Fax : __________________ Signature :

28. ANNEXE 9 - AUTORISATION DE LIEUX

Fax émis par : 33 0155873642 ESSAIS CLINIQUES 27/05/02 17:19 Pg: 2/2

AGENCE FRANÇAISE DE SECURITE SANITAIRE DES PRODUITS DE SANTE

RÉPUBLIQUE FRANÇAISE

N° du lieu : 03046MHC

DECISION DU 27 MAI 2002

AUTORISANT UN LIEU

DE RECHERCHES BIOMEDICALES SANS BENEFICE INDIVIDUEL DIRECT

LE DIRECTEUR GENERAL DE L'AGENCE FRANCAISE

DE SECURITE SANITAIRE DES PRODUITS DE SANTE

Vu le code de la santé publique et notamment ses articles L.5311-1, L.1121-3, L.1124-6 et R.2021 à R.2027 ;
Vu la demande adressée au préfet de la région Auvergne le 21 janvier 2002, complétée le 11 avril 2002 ;
Vu le rapport d'enquête du médecin et du pharmacien inspecteurs de santé publique en date du 11 avril 2002, complété en date du 19 avril 2002 et du 22 avril 2002 ;

DECIDE:

ARTICLE 1er - L'autorisation mentionnée à l'article L.1124-6 du code de la santé publique est accordée, pour effectuer des recherches biomédicales sans bénéfice individuel direct au :

CENTRE DE PHARMACOLOGIE CLINIQUE ET
CENTRE DE RECHERCHE CLINIQUE ET VACCINOLOGIQUE (CPC / CRC & V)
Bâtiment 3C – 1er étage
Hôpital Gabriel Montpied - CHU
B.P. 69
63009 CLERMONT-FERRAND Cedex 1

placé sous la responsabilité de Monsieur Claude DUBRAY, dans les conditions prévues à l'article 2.
Les recherches ayant trait au médicament devront être conduites avec la participation de Monsieur DISSAIT, réanimateur.

ARTICLE 2 - Cette autorisation concerne les recherches biomédicales sans bénéfice individuel direct, conduites chez le volontaire majeur, ayant trait :

- au médicament dans le cadre d'études de tolérance, de pharmacodynamie et de pharmacocinétique incluant, notamment, la biodisponibilité et la bioéquivalence,

- aux dispositifs médicaux,

- aux produits cosmétiques,

- aux aliments diététiques destinés à des fins médicales spéciales qui, du fait de leur composition, sont susceptibles de présenter un risque pour les personnes auxquelles ils ne sont pas destinés.

Le Directeur Général

Philippe DUNETON

143/147, Boulevard Anatole France 93285 Saint-Denis Cedex - Tél. 01.55.87.30.00

29. ANNEXE 10 - Engagement à la méthodologie de référence de la CNIL

CNIL
Commission Nationale de l'Informatique et des Libertés

Récépissé de déclaration
de conformité à une méthodologie de référence

Numéro de déclaration
1223379

Monsieur Bernard BELAIGUES
CHU DE CLERMONT FERRAND
58 RUE MONTALEMBERT
BP 69
63003 CLERMONT FERRAND CEDEX

Informations enregistrées par la commission :

Organisme déclarant

Statut : Secteur public
N° SIREN ou SIRET : 266307461
Code NAF ou APE : 851A
Nom : CHU DE CLERMONT FERRAND
Adresse : 58 RUE MONTALEMBERT BP 69 63003 CLERMONT FERRAND CEDEX
Tél : 0473751195 Fax : 0473754730 Messagerie :

Contact

Nom : Bruno AUBLET CUVELIER CHU DE CLERMONT FERRAND DEPARTEMENT D'INFORMATION MEDICALE UNITE DE SOUTIEN METHODOLOGIQUE A LA RECHERCHE CLINIQUE
Adresse : 58 RUE MONTALEMBERT BP 69 63003 CLERMONT FERRAND CEDEX
Tél : 0473751195 Fax : 0473754730 Messagerie :

Traitement déclaré

Méthodologie de référence : MR-001
Finalité : Méthodologie de recherches biomédicales

Personne responsable de la déclaration

Nom : Bernard BELAIGUES CHU DE CLERMONT FERRAND
Fonction : Directeur Général Adjoint
Date de la déclaration : 15-03-2007

La délivrance du présent récépissé n'exonère le déclarant d'aucune de ses responsabilités.

Paris, le 16 mars 2007
Par délégation de la commission

Alex TÜRK
Président de la commission

Si votre déclaration a été établie sur un formulaire papier, celui-ci ne sera pas conservé par la CNIL au-delà d'un délai d'un mois à compter de ce jour.

8, rue Vivienne - CS 30223 - 75083 Paris Cedex 02 Tél : 01 53 73 22 22 Fax : 01 53 73 22 00 Site : http://www.cnil.fr
REPUBLIQUE FRANCAISE

30.ANNEXE 11 – Avis du Comité Technique COMVAL

CENTRE DE PHARMACOLOGIE CLINIQUE
CENTRE D'INVESTIGATION CLINIQUE
(Inserm CIC 501)
Bât 3 C – CHU de Clermont-Ferrand
BP 69 - 63003 – CLERMONT-FERRAND
Tel : 04.73.17.84.10 – Fax 04.73.17.84.12

Clermont-Ferrand, le 26/07/2011

Chère Madame,

Nous vous prions de prendre connaissance de l'évaluation de votre projet présenté au Comité de Validation des projets de recherche clinique.

Date de la réunion	25/07/2011
Membres du CT présents	Dualé C, Durando X, Laclautre L, Pereira B, Pickering G.
Membres du CT excusés	Creveaux I, Dubray C, Ughetto S.
Investigateurs présents	Barber-Chamoux N, Pickering G.
Titre du projet soumis	Prévention du développement de douleur neuropathique post-mastectomie par l'administration de mémantine en pré- et post-chirurgie (étude multicentrique, randomisée, en double aveugle, en groupes parallèles, contrôlée versus placebo).
Nom de l'investigateur principal	Pickering G.
Service	CPC-CIC
Pôle du CHU	Recherche Clinique
Collaborateurs destinataires du courrier	Macian N., Pereira B.

Cadre règlementaire de la recherche	
Recherche biomédicale (RBM)	X
Recherche sur les soins courants (RSC)	
Collection d'échantillons biologiques nouvelle ou existante avec changement de finalité (CEB)	
Recherche observationnelle hors RBM (RO)	

RBM : nécessite l'avis favorable d'un CPP et l'autorisation de l'AFSSAPS
RSC : nécessite l'avis favorable d'un CPP et une déclaration nominale de fichier à la CNIL
CEB : nécessite l'avis favorable d'un CPP et une déclaration au Ministère de la Recherche
RO : nécessite une déclaration nominale de fichier à la CNIL ; un avis éthique hors CPP est recommandé pour la publication des résultats

Type de recherche			
Etude de physiologie		Etude en santé publique	
Etude de physiopathologie		Evaluation des technologies	
Etude de pharmacologie clinique	X	Recherche sur dispositifs médicaux	
Essai thérapeutique	X	Recherche portant sur les soins courants	
Evaluation de méthode(s) diagnostique(s)		Autre (préciser) :	
Evaluation médico-économique			
Etude épidémiologique			

Avec nos sentiments les meilleurs,

Dr Christian DUALÉ, Médecin Délégué

Pr Claude DUBRAY, Médecin Coordonnateur

**CRITERES D'EVALUATION DU PROJET**	**A**	**B**	**C**	**D**	**NA**	**N°**
**Argumentation scientifique**						
Originalité	X				/	
Qualité de l'hypothèse	X					
Utilité pour les malades ou la connaissance médicale	X				/	
**Faisabilité**						
Potentiel de recrutement		X				1
Validité des actes ou des pratiques cliniques	X					
Maîtrise des méthodes	X				/	
Organisation pratique		X			/	1, 2
Faisabilité pharmaceutique / biomédicale	X					
Ethique et protection des personnes		X			/	2
Adéquation des moyens humains, matériels et financiers		X			/	3
**Méthodologie**						
Objectif principal	X					
Objectifs secondaires	X					
Méthodes et plan expérimental	X					
Constitution des groupes	X					
Critères de sélection des patients		X				1
Méthode de randomisation		X				1, 4
Critère(s) de jugement principal (-aux)	X					
Critères de jugement secondaires		X				5
Calcul de la taille des échantillons	X					
Règles d'arrêt	X				/	
Procédure de notification des évènements indésirables graves	X					
Stratégie d'analyse statistique	X					
**Informations générales**						
Qualité rédactionnelle et présentation	X				/	
Prise en compte des aspects réglementaires		X				2
Description des sources de financement		X				3
Adaptation des sources de financement		X			/	3
**Décision du Comité Technique**		X			/	

NA : non applicable

A. Validé sans modification
B. Validé avec modifications mineures ne donnant pas lieu à une évaluation ultérieure du CT
C. Réserves de modifications notables devant être vérifiées par le Coordonnateur ou le Médecin Délégué avant validation
D. Réserves de modifications majeures nécessitant re-soumission à une réunion ultérieure du CT.

Commentaires en page suivante

☞

1. Sélection des patients, potentiel de recrutement, éventuelles conséquences sur l'organisation pratique et la randomisation. La chirurgie du cancer du sein s'inscrit dans une démarche de soins complexe et multimodale, d'autres thérapeutiques pouvant avoir un effet sur le critère principal : notamment le type de chirurgie (mastectomie partielle ou totale, et surtout avec ou sans curage axillaire ; chimiothérapie adjuvante par taxanes ; reconstruction mammaire immédiate, voire à distance avant le 3ème mois postopératoire). Afin de contrôler au mieux ces facteurs, deux options sont possibles :
    a. sélectionner une sous-population homogène, bénéficiant du même plan thérapeutique, pour autant que la chirurgie soit génératrice de douleur chronique (ce qui est peu probable pour une mastectomie partielle sans curage) ; ceci aurait pour effet de ralentir le recrutement et de conduire à l'ouverture de plusieurs centres ;
    b. accepter la diversité, en faisant l'hypothèse que la randomisation équilibrera tous les facteurs entre les deux bras ; une stratification sur les thérapeutiques prévues au moment de l'inclusion est à étudier.

    A noter qu'il est préférable d'avoir une technique d'anesthésie standardisée, notamment pour ce qui concerne l'usage des produits ou techniques suivants : anesthésie loco-régionale, kétamine, gabapentine.
2. La procédure de recrutement et de suivi des patients est à préciser, notamment :
    a. identification des personnes susceptibles d'être incluses (réunion de concertation pluridisciplinaire, consultation chirurgicale…) ;
    b. délivrance de l'information orale et écrite ;
    c. recueil du formulaire de consentement après délai de réflexion ;
    d. délivrance du traitement d'étude ;
    e. lieu(x) de visite au 3ème et au 6ème mois après chirurgie ;
    f. médecins investigateurs impliqués dans ces différentes procédures.
3. Lister au chapitre 16 l'ensemble des surcoûts et leur(s) source(s) de financement, le cas échéant.
4. Décrire la méthode de randomisation (taille des blocs, liste complémentaire le cas échéant).
5. Critères secondaires de jugement. D'autres types d'évaluations peuvent être proposées, laissées au libre choix de l'investigateur selon leur pertinence (cf. références jointes) :
    a. Douleur chronique : *Brief pain inventory* (Gjeilo & coll., Mcdermott & coll., Skljarevski & coll.) ;
    b. Douleur neuropathique (Hampaa & coll.) ;
    c. Interaction entre le sommeil et la douleur neuropathique (Van Seventer & coll.) ;
    d. Qualité de vie adaptée aux personnes porteuses de cancer : EORTC-QLQ-C30 (Schwenk & coll.).

31. ANNEXE 12 – Questionnaires

QUESTIONNAIRE DN4 : un outil simple pour rechercher les douleurs neuropathiques

Pour estimer la probabilité d'une douleur neuropathique, le patient doit répondre à chaque item des 4 questions ci dessous par « oui » ou « non ».

QUESTION 1 : la douleur présente-t-elle une ou plusieurs des caractéristiques suivantes ?

	Oui	Non
1. Brûlure	☐	☐
2. Sensation de froid douloureux	☐	☐
3. Décharges électriques	☐	☐

QUESTION 2 : la douleur est-elle associée dans la même région à un ou plusieurs des symptômes suivants ?

	Oui	Non
4. Fourmillements	☐	☐
5. Picotements	☐	☐
6. Engourdissements	☐	☐
7. Démangeaisons	☐	☐

QUESTION 3 : la douleur est-elle localisée dans un territoire où l'examen met en évidence :

	Oui	Non
8. Hypoesthésie au tact	☐	☐
9. Hypoesthésie à la piqûre	☐	☐

QUESTION 4 : la douleur est-elle provoquée ou augmentée par :

	Oui	Non
10. Le frottement	☐	☐

OUI = 1 point **NON = 0 point** **Score du Patient : /10**

MODE D'EMPLOI

Lorsque le praticien suspecte une douleur neuropathique, le questionnaire DN4 est utile comme outil de diagnostic.

Ce questionnaire se répartit en 4 questions représentant 10 items à cocher :

- ✓ Le praticien interroge lui-même le patient et remplit le questionnaire
- ✓ A chaque item, il doit apporter une réponse « oui » ou « non »
- ✓ A la fin du questionnaire, le praticien comptabilise les réponses, 1 pour chaque « oui » et 0 pour chaque « non ».
- ✓ La somme obtenue donne le Score du Patient, noté sur 10.

Si le score du patient est égal ou supérieur à 4/10, le test est positif
(sensibilité à 82,9 % ; spécificité à 89,9 %)

D'après Bouhassira D *et al. Pain* 2004 ; 108 (3) : 248-57.

Questionnaire NPSI

(Neuropathic Pain Symptom Inventory - 2004)

Intensité des douleurs spontanées

Q1. Douleur ressentie comme une brûlure ?
Q2. Douleur ressentie comme un étau ?
Q3. Douleur ressentie comme une compression ?
Q4. Douleurs spontanées présentes : en permanence / 8 à 12h / 4 à 7h / 1 à 3h / <1h

Intensité des crises douloureuses

Q5. Crises douloureuses ressenties comme des décharges électriques ?
Q6. Crises douloureuses ressenties comme des coups de couteau ?
Q7. Crises douloureuses présentées ? >20 / 11 à 20 / 6 à 10 / 1 à 5 / aucune

Intensité des douleurs provoquées

Q8. Douleurs provoquées ou augmentées par le frottement sur la zone douloureuse ?
Q9. Douleurs provoquées ou augmentées par la pression sur la zone douloureuse ?
Q10. Douleurs provoquées ou augmentées par le contact avec un objet froid sur la zone douloureuse ?

Intensité des sensations anormales

Q11. A type de picotements ?
Q12. A type de fourmillements ?

10 descripteurs (EN 0 à 10) - Score global : 100
2 items : durée douleurs spontanées / fréquence paroxysmes douloureux
(score / item plus sensible que score global)

(Bouhassira et al. Pain 2004)

Détails pages suivantes

Inventaire des symptômes de la douleur neuropathique : questionnaire NPSI

- Liste de 12 questions élaborée à partir de symptômes (descripteurs subjectifs) considérés comme évocateurs de la douleur neuropathique (validés comme tels au cours de l'élaboration de DN4).
- Auto-questionnaire
  Nous voudrions savoir si vous avez des douleurs spontanées, c'est-à-dire des douleurs en l'absence de toute stimulation. Pour chacune des questions suivantes, entourez le chiffre qui correspond le mieux à l'intensité de vos douleurs spontanées en moyenne au cours des 24 dernières heures. Entourez le chiffre 0 si vous n'avez pas ressenti ce type de douleur. Veuillez n'entourez qu'un seul chiffre.
- Cotation de l'intensité des symptômes en 11 points ou estimation de leur fréquence :

Q1. Votre douleur est-elle ressentie comme une brûlure ?

Aucune Brûlure	0	1	2	3	4	5	6	7	8	9	10	Brûlure maximale imaginable

- Intensité des douleurs spontanées :
  - Q1. Douleur ressentie comme une brûlure ?
  - Q2. Douleur ressentie comme un étau ?
  - Q3. Douleur ressentie comme une compression ?
  - Q4. Douleur spontanées présentes : en permanence / 8 à 12 h / 4 à 7 h / 1 à 3 h / < 1 h

- Intensité des crises douloureuses :
  - Q5. Crises douloureuses ressenties comme des décharges électriques ?
  - Q6. Crises douloureuses ressenties comme des coups de couteau ?
  - Q7. Crises douloureuses présentées ? > 20 / 11 à 20 / 6 à 10 / 1 à 5 / aucune

- Intensité des douleurs provoquées :
  - Q8. Douleurs provoquées ou augmentées par le frottement sur la zone douloureuse ?

  - Q9. Douleurs provoquées ou augmentées par la pression sur la zone douloureuse ?
  - Q10. Douleurs provoquées ou augmentées par le contact avec un objet froid sur la zone douloureuse ?

- Intensité des sensations anormales :
  - Q11. A type de picotements ?
  - Q12. A type de fourmillements ?

- Calcul des scores
  - Score total (10 questions cotées) sur 100,
  - Sous scores :
    - Brûlures (douleurs spontanées superficielles) :
      - Q1 = ……………………../10
    - Constriction (douleurs spontanées profondes) :
      - (Q2 + Q3) / 2 = …………../10
    - Douleurs paroxystiques :
      - (Q5 + Q6) / 2 = …………../10
    - Douleurs évoquées :
      - (Q8 + Q9 + Q10) / 3 = …………../10
    - Paresthésies / dysesthésies :
      - (Q11 + Q12) / 2 = …………../10

Figure 2.

Questionnaire concis sur les douleurs (version courte) – modifié

Nom____ Date ________________________________

Indiquez sur ce schéma où se trouve votre douleur en noircissant la zone. Mettez un « X » à l'endroit où vous ressentez la douleur la plus intense. *(V = vive / en coup de poignard, B = à type de brûlure, E = engourdissement, P = picotements, S = sourde et persistante, flèches = élancements. Utilisez des couleurs différentes si vous ressentez plus d'un type de douleur.)*

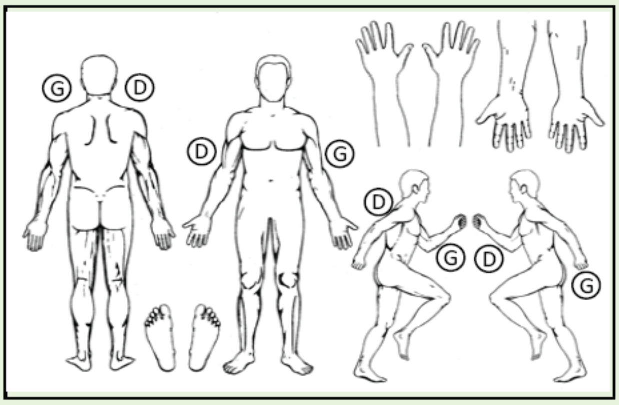

Qu'est-ce qui intensifie votre douleur?

Qu'est-ce qui atténue votre douleur?

Quels traitements ou médicaments recevez-vous actuellement contre la douleur?

Veuillez encercler le chiffre qui décrit le mieux la douleur la plus **INTENSE** que vous avez ressentie dans les 24 dernières heures.

Pas de douleur	0	1	2	3	4	5	6	7	8	9	10	Douleur la + horrible que vous puissiez imaginer

Veuillez encercler le chiffre qui décrit le mieux la douleur la plus **FAIBLE** que vous avez ressentie dans les 24 dernières heures.

Pas de douleur	0	1	2	3	4	5	6	7	8	9	10	Douleur la + horrible que vous puissiez imaginer

Veuillez encercler le chiffre qui décrit le mieux votre douleur **EN GÉNÉRAL**.

Pas de douleur	0	1	2	3	4	5	6	7	8	9	10	Douleur la + horrible que vous puissiez imaginer

Veuillez encercler le chiffre qui décrit le mieux votre douleur **EN CE MOMENT**.

Pas de douleur	0	1	2	3	4	5	6	7	8	9	10	Douleur la + horrible que vous puissiez imaginer

Dans les 24 dernières heures, quel soulagement les traitements ou les médicaments que vous prenez vous ont-ils apporté? Pouvez-vous indiquer le pourcentage d'**AMÉLIORATION** obtenue?

Aucune amélioration	0 %	10 %	20 %	30 %	40 %	50 %	60 %	70 %	80 %	90 %	100 %	Amélioration complète

Échelle de gêne

Encerclez le chiffre qui décrit le mieux comment, dans les 24 dernières heures, la douleur a gêné :

A. votre activité générale

Ne gêne pas	0	1	2	3	4	5	6	7	8	9	10	Gêne complètement

B. votre humeur

Ne gêne pas	0	1	2	3	4	5	6	7	8	9	10	Gêne complètement

C. votre capacité à marcher

Ne gêne pas	0	1	2	3	4	5	6	7	8	9	10	Gêne complètement

D. votre travail habituel (y compris à l'extérieur de la maison et les travaux domestiques)

Ne gêne pas	0	1	2	3	4	5	6	7	8	9	10	Gêne complètement

E. vos relations avec les autres

Ne gêne pas	0	1	2	3	4	5	6	7	8	9	10	Gêne complètement

F. votre sommeil

Ne gêne pas	0	1	2	3	4	5	6	7	8	9	10	Gêne complètement

G. votre goût de vivre

Ne gêne pas	0	1	2	3	4	5	6	7	8	9	10	Gêne complètement

Source : Adapté de Pain Research Group, 1997[12]. Réimpression autorisée.

Echelle HAD*

Echelle du retentissement émotionnel : HAD

Les médecins savent que les émotions jouent un rôle important dans la plupart des maladies. Si votre médecin est au courant des émotions que vous éprouvez, il pourra mieux vous aider. Ce questionnaire a été conçu de façon à permettre à votre médecin de se familiariser avec ce que vous éprouvez vous-même sur le plan émotif.

Ne faites pas attention aux chiffres et aux lettres imprimés à gauche du questionnaire. Lisez chaque série de questions et soulignez la réponse qui exprime le mieux ce que vous avez éprouvé au cours de la semaine qui vient de s'écouler.

Ne vous attardez pas sur la réponse à faire, votre réaction immédiate à chaque question fournira probablement une meilleure indication de ce que vous éprouvez, qu'une réponse longuement méditée.

D	A	
		**Je me sens tendu ou énervé**
	3	la plupart du temps
	2	souvent
	1	de temps en temps
	0	jamais
		**Je prends plaisir aux mêmes choses qu'autrefois :**
0		oui, tout autant
1		pas autant
2		un peu seulement
3		presque plus
		**J'ai une sensation de peur comme si quelque chose d'horrible allait m'arriver :**
	3	oui, très nettement
	2	oui, mais ce n'est pas grave
	1	un peu, mais cela ne m'inquiète pas
	0	pas du tout
		**Je ris facilement et vois le bon côté des choses :**
0		autant que par le passé
1		plus autant qu'avant
2		vraiment moins qu'avant
3		plus du tout
		**Je me fais du souci**
	3	très souvent
	2	assez souvent
	1	occasionnellement
	0	très occasionnellement
		**Je suis de bonne humeur**
3		jamais
2		rarement
1		assez souvent
0		la plupart du temps

*Source : Evaluation et suivi de la douleur chronique chez l'adulte en médecine ambulatoire. ANAES, Février 1999.

Echelle HAD*

D	A	
		**Je peux rester tranquillement assis à ne rien faire et me sentir décontracté :**
	0	oui, quoi qu'il arrive ☐
	1	oui, en général ☐
	2	rarement ☐
	3	jamais ☐
		**J'ai l'impression de fonctionner au ralenti :**
3		presque toujours ☐
2		très souvent ☐
1		parfois ☐
0		jamais ☐
		**J'éprouve des sensations de peur et j'ai l'estomac noué :**
	0	jamais ☐
	1	parfois ☐
	2	assez souvent ☐
	3	très souvent ☐
		**Je ne m'intéresse plus à mon apparence :**
3		plus du tout ☐
2		je n'y accorde pas pas autant d'attention que je le devrais ☐
1		il se peut que je n'y fasse plus autant attention ☐
0		j'y prête autant d'attention que par le passé ☐
		**J'ai la bougeotte et n'arrive pas à tenir en place :**
	3	oui, c'est tout à fait le cas ☐
	2	un peu ☐
	1	pas tellement ☐
	0	pas du tout ☐
		**Je me réjouis d'avance à l'idée de faire certaines choses**
0		autant qu'auparavant ☐
1		un peu moins qu'avant ☐
2		bien moins qu'avant ☐
3		presque jamais ☐
		**J'éprouve des sensations soudaines de panique :**
	3	vraiment très souvent ☐
	2	assez souvent ☐
	1	pas très souvent ☐
	0	jamais ☐
		**Je peux prendre plaisir à un bon livre ou à une bonne émission radio ou de télévision :**
0		souvent ☐
1		parfois ☐
2		rarement ☐
3		très rarement ☐

*Source : Evaluation et suivi de la douleur chronique chez l'adulte en médecine ambulatoire. ANAES, Février 1999.

Echelle du retentissement émotionnel (HAD : Hospital Anxiety and Depression scale)*

Note explicative pour le praticien.

- *Mode de passation :*
  - Il est recommandé de demander au patient de bien lire le préambule avant de remplir l'échelle.

- *Cotation :*
  - Chaque réponse est cotée de 0 à 3 sur une échelle évaluant de manière semi-quantitative l'intensité du symptôme au cours de la semaine écoulée.
  - L'intervalle des notes possibles s'étend donc pour chaque échelle de 0 à 21, les scores les plus élevés correspondent à la présence d'une symptomatologie plus sévère. La présentation de l'échelle, avec dans les colonnes de gauche des notes correspondant à chaque item, facilite un calcul rapide des scores.
  - Les notes de la dépression (reconnaissables par la lettre « D ») se situent dans la première colonne de gauche. Les notes de la colonne « dépression » doivent être additionnées ensemble pour obtenir le score de la dépression.
  - Les notes de l'anxiété (reconnaissables par la lettre « A ») se situent dans la seconde colonne de gauche. Les notes de la colonne « anxiété » doivent être additionnées ensemble pour obtenir le score de l'anxiété.
  - Pour les deux scores (dépression et anxiété), des valeurs seuils ont été déterminées :

- Un score inférieur ou égal à 7 = absence de cas ;
- Un score entre 8 et 10 = cas douteux ;
- Un score supérieur ou égal à 11 = cas certain .

*Source : Evaluation et suivi de la douleur chronique chez l'adulte en médecine ambulatoire. ANAES, Février 1999.

Questionnaire de santé SF36

Date |__|__| |__|__| |__|__|

Comment répondre

Les questions qui suivent portent sur votre santé, telle que vous la ressentez. Ces informations nous permettront de mieux savoir comment vous vous sentez dans votre vie de tous les jours.

Veuillez répondre à toutes les questions en entourant le chiffre correspondant à la réponse choisie, comme il est indiqué. Si vous ne savez pas très bien comment répondre, choisissez la réponse la plus proche de votre situation.

Identification

1. Dans l'ensemble, pensez-vous que votre santé est : *(entourez la réponse de votre choix)*

Excellente	1
Très bonne	2
Bonne	3
Médiocre	4
Mauvaise	5

2. Par rapport à l'année dernière à la même époque, comment trouvez-vous votre état de santé en ce moment ? *(entourez la réponse de votre choix)*

Bien meilleur que l'an dernier	1
Plutôt meilleur	2
À peu près pareil	3
Plutôt moins bon	4
Beaucoup moins bon	5

3. Au cours de ces 4 dernières semaines, et en raison de votre état physique
(entourez la réponse de votre choix, une par ligne)

	Oui	Non
a. Avez-vous réduit le temps passé à votre travail ou à vos activités habituelles ?	1	2
b. Avez-vous accompli moins de choses que vous auriez souhaité ?	1	2
c. Avez-vous dû arrêter de faire certaines choses ?	1	2
d. Avez-vous eu des difficultés à faire votre travail ou toute autre activité ? (par exemple, cela vous a demandé un effort supplémentaire)	1	2

4. Au cours de ces 4 dernières semaines, et en raison de votre état émotionnel (comme vous sentir triste, nerveux(se) ou déprimé(e))
(entourez la réponse de votre choix, une par ligne)

	Oui	Non
a. Avez-vous réduit le temps passé à votre travail ou à vos activités habituelles	1	2
b. avez-vous accompli moins de choses que vous auriez souhaité	1	2
c. avez-vous eu des difficultés à faire ce que vous aviez à faire avec autant de soin et d'attention que d'habitude	1	2

5. Au cours de ces 4 dernières semaines dans quelle mesure votre état de santé, physique ou émotionnel, vous a-t-il gêné(e) dans votre vie sociale et vos relations avec les autres, votre famille, vos amis, vos connaissances
(entourez la réponse de votre choix)

Pas du tout	1
Un petit peu	2
Moyennement	3
Beaucoup	4
Enormément	5

6. Au cours de ces 4 dernières semaines, quelle a été l'intensité de vos douleurs (physiques) ?
(entourez la réponse de votre choix)

Nulle	1
Très faible	2
Faible	3
Moyenne	4
Grande	5
Très grande	6

7. Au cours de ces 4 dernières semaines, dans quelle mesure vos douleurs physiques vous ont-elles limité(e) dans votre travail ou vos activités domestiques? *(entourez la réponse de votre choix)*

Pas du tout	1
Un petit peu	2
Moyennement	3
Beaucoup	4
Enormément	5

8. Au cours de ces 4 dernières semaines. y a-t-il eu des moments où votre état de santé. physique ou émotionnel, vous a gêné(e) dans votre vie et vos relations avec les autres, votre famille, vos amis, vos connaissances ?
(entourez la réponse de votre choix)

En permanence	1
Une bonne partie du temps	2
De temps en temps	3
Rarement	4
Jamais	5

9. Voici une liste d'activités que vous pouvez avoir à faire dans votre vie de tous les jours. Pour chacune d'entre elles indiquez si vous êtes limité(e) en raison de votre état de santé actuel.
(entourez la réponse de votre choix, une par ligne)

	Liste d'activités	Oui, beaucoup limité(e)	Oui, un peu limité(e)	Non, pas du tout limité(e)
a.	Efforts physiques importants tels que courir, soulever un objet lourd, faire du sport	1	2	3
b.	Efforts physiques modérés tels que déplacer une table, passer l'aspirateur, jouer aux boules	1	2	3
c.	Soulever et porter les courses	1	2	3
d.	Monter plusieurs étages par l'escalier	1	2	3
e.	Monter un étage par l'escalier	1	2	3
f.	Se pencher en avant, se mettre à genoux, s'accroupir	1	2	3
g.	Marcher plus d'un km à pied	1	2	3
h.	Marcher plusieurs centaines de mètres	1	2	3
i.	Marcher une centaine de mètres	1	2	3
j.	Prendre un bain, une douche ou s'habiller	1	2	3

10. Les questions qui suivent portent sur comment vous vous êtes senti(e) au cours de ces 4 dernières semaines. Pour chaque question, veuillez indiquer la réponse qui vous semble la plus appropriée. Au cours de ces 4 dernières semaines, y a-t-il eu des moments où :
(entourez la réponse de votre choix, une par ligne)

	En permanence	Très souvent	Souvent	Quelque fois	Rarement	Jamais
a. vous vous êtes senti(e) dynamique?	1	2	3	4	5	6
b. vous vous êtes senti(e) très nerveux(se)?	1	2	3	4	5	6
c. vous vous êtes senti(e) si découragé(e) que rien ne pouvait vous remonter le moral?	1	2	3	4	5	6
d. vous vous êtes senti(e) calme et détendu(e)?	1	2	3	4	5	6
e. vous vous êtes senti(e) débordant(e) d'énergie?	1	2	3	4	5	6
f. vous vous êtes senti(e) triste et abattu(e)?	1	2	3	4	5	6
9. vous vous êtes senti(e) épuisé(e)?	1	2	3	4	5	6
h. vous vous êtes senti(e) heureux(se)?	1	2	3	4	5	6
i. vous vous êtes senti(e) fatigué(e)?	1	2	3	4	5	6

11. Indiquez pour chacune des phrases suivantes dans quelle mesure elles sont vraies ou fausses dans votre cas :
(entourez la réponse de votre choix , une par ligne)

	Totalement vrai	Plutôt vrai	Je ne sais pas	Plutôt fausse	Totalement fausse
a. Je tombe malade plus facilement que les autres	1	2	3	4	5
b. Je me porte aussi bien que n'importe qui	1	2	3	4	5
c. Je m'attends à ce que ma santé se dégrade	1	2	3	4	5
d. Je suis en excellent santé	1	2	3	4	5

Veuillez vérifier que vous avez bien fourni une réponse pour chacune des questions. Merci de votre collaboration.
copyright © New England Medical Center Hospitals, Inc., 1993 All rights reserved. (IQOLA SF-36 French (France) Version 1 3)

Questionnaire d'évaluation du sommeil de Leeds

Le questionnaire d'évaluation du sommeil de Leeds (QESL) est un auto-questionnaire normalisé composé de dix échelles visuelles analogiques qui ont trait à quatre aspects de l'efficacité du sommeil :
- la qualité de l'endormissement, degré de somnolence (1,2,3),
- la qualité du sommeil (4,5),
- la qualité du réveil : (6,7,8)
- la qualité de l'état suivant le réveil , performances (9, 10)

En pratique, on l'utilise pour évaluer l'efficacité et la tolérance des produits psychotropes (susceptibles de modifier le niveau de vigilance) au cours des essais thérapeutiques, ou a l'initiation d'un nouveau traitement.
Il peut servir à mesurer les progrès réalisés grâce à *l'autogestion non médicamenteuse* de l'insomnie, que nous proposons sur ce site.
Adapté du *Leeds Sleep Evaluation Questionnaire* pour *Sommeil et médecine générale.* (Référence : Parrott A.C., Hindmarch I., The Leeds Sleep Evaluation Questionnaire in Psychopharmacological Investigations - a Rewiew, Psychopharmacology, II, 123-179, 1980).
Indiquez votre réponse à chaque question par une croix sur la ligne horizontale.
La position indique le degré de modification que vous observez.
Si vous ne notez aucune modification ; placez le trait au milieu de la ligne.

Comment estimez-vous la manière dont vous vous *endormez* par rapport à d'habitude ?

difficile <---5---4---3---2---1---0---1---2---3---4---5-> facile

plus long <---5---4---3---2---1---0---1---2---3---4---5-> moins long

pas sommeil <—5---4---3---2---1---0---1---2---3---4---5—> beaucoup sommeil

Comment jugez-vous votre sommeil par rapport à d'habitude :

moins reposant <—5---4---3---2---1---0---1---2---3---4---5-> plus reposant

plus fragmenté <—5---4---3---2---1---0---1---2---3---4---5—> moins fragmenté

Comment estimez-vous la manière dont vous vous *réveillé(e)* par rapport à d'habitude ?

difficilement <—5---4---3---2---1---0---1---2---3---4---5—> facilement

lentement <---5---4---3---2---1—0---1---2---3---4---5—> rapidement

Comment estimez-vous votre niveau de forme au réveil ?

très fatigué <—5---4---3---2---1---0---1---2---3---4---5—> bien en forme

Comment vous sentez-vous en ce moment dans la journée ?

très fatigué<---5---4---3---2---1---0---1---2---3---4---5-> bien en forme

Que diriez-vous de votre niveau d'équilibre et de coordination au réveil ?

très perturbé <---5---4---3---2---1---0---1---2---3---4---5-> normal

Le score pour chaque item est la somme des distances séparant le repère central.

Les distances sont négatives à gauche, et positive à droite du trait.

Questionnaire QDSA

Questionnaire Douleur de Saint Antoine : QDSA

Qualificatif des douleurs

Afin de préciser la douleur que vous ressentez actuellement (depuis les dernières 48h), nous vous demandons de répondre au questionnaire ci-après.

Il vous aidera à préciser :

les mots qui décrivent votre douleur,
les changements dans vos activités
votre état d'humeur et de tension

➔ N'oubliez pas de répondre à toutes les questions. Ce questionnaire aidera à mieux définir votre traitement et à suivre l'évolution de vos progrès.

Vous trouverez ci-dessous une liste de mots pour décrire votre douleur.

➔ Afin de préciser la douleur que vous ressentez depuis les dernières 48h, entourez pour chaque mot la note correspondante.

Cotations
0 = absent 1 = faible 2 = modéré 3 = fort 4 = extrêmement fort

➔ Ensuite, indiquez en face de chaque mot l'endroit concerné par la douleur (la localisation).

➔ Pensez à apporter ce questionnaire rempli à la consultation. Merci.

Commencer le test en vous demandant avant chaque terme :
« est-ce que pour mes douleurs je ressens… » (des battements),
« est-ce que pour mes douleurs je ressens… » (des pulsations) …

Nom : Prénom : Date de naissance : Date :

Cotations 0 = absent 1 = faible 2 = modéré 3 = fort 4 = extrêmement fort **LOCALISATION**

A	Battements	0	1	2	3	4
	Pulsations	0	1	2	3	4
	Elancements	0	1	2	3	4
	En éclairs	0	1	2	3	4
	Décharges électriques	0	1	2	3	4
	Coups de marteau	0	1	2	3	4
B	Rayonnante	0	1	2	3	4
	Irradiante	0	1	2	3	4
C	Piqûre	0	1	2	3	4
	Coupure	0	1	2	3	4
	Pénétrante	0	1	2	3	4
	Transperçante	0	1	2	3	4
	Coups de poignard	0	1	2	3	4
D	Pincement	0	1	2	3	4
	Serrement	0	1	2	3	4
	Compression	0	1	2	3	4
	Ecrasement	0	1	2	3	4
	En étau	0	1	2	3	4
	Broiement	0	1	2	3	4
E	Tiraillement	0	1	2	3	4
	Etirement	0	1	2	3	4
	Distension	0	1	2	3	4
	Déchirure	0	1	2	3	4
	Torsion	0	1	2	3	4
	Arrachement	0	1	2	3	4
F	Chaleur	0	1	2	3	4
	Brûlure	0	1	2	3	4
G	Froid	0	1	2	3	4
	Glace	0	1	2	3	4

Nom : Prénom : Date de naissance : Date :

Cotations 0 = absent 1 = faible 2 = modéré 3 = fort 4 = extrêmement fort **LOCALISATION**

H	Picotements	0	1	2	3	4	
	Fourmillement	0	1	2	3	4	
	Démangeaisons	0	1	2	3	4	
I	Engourdissement	0	1	2	3	4	
	Lourdeur	0	1	2	3	4	
	Sourde	0	1	2	3	4	
J	Fatigante	0	1	2	3	4	
	Epuisante	0	1	2	3	4	
	Ereintante	0	1	2	3	4	
K	Nauséeuse	0	1	2	3	4	
	Suffocante	0	1	2	3	4	
	Syncopale	0	1	2	3	4	
L	Inquiétante	0	1	2	3	4	
	Oppressante	0	1	2	3	4	
	Angoissante	0	1	2	3	4	
M	Harcelante	0	1	2	3	4	
	Obsédante	0	1	2	3	4	
	Cruelle	0	1	2	3	4	
	Torturante	0	1	2	3	4	
	Suppliciante	0	1	2	3	4	
N	Gênante	0	1	2	3	4	
	Désagréable	0	1	2	3	4	
	Pénible	0	1	2	3	4	
	Insupportable	0	1	2	3	4	
O	Enervante	0	1	2	3	4	
	Exaspérante	0	1	2	3	4	
	Horripilante	0	1	2	3	4	
P	Déprimante	0	1	2	3	4	
	Suicidaire	0	1	2	3	4	

TRAIL MAKING TEST (TMT)
PARTIE A

Consigne :

Montrer la feuille-exemple (Sample) au sujet et lui dire : « Sur cette feuille se trouvent huit cercles numérotés de 1 à 8. Je vous demande de relier ces huit cercles avec ce crayon, en suivant l'ordre numérique, c'est-à-dire en allant de 1 à 2, de 2 à 3, puis de 3 à 4…Vous avez compris ? ». Si le sujet semble ne pas comprendre, lui montrer ce qu'il doit faire avec le crayon, puis lui demander de refaire l'exemple avec une autre feuille. Lorsque le sujet a compris, lui présenter la feuille de test et lui dire : « Maintenant, nous allons faite la même chose avec cette feuille. Cette fois, il y a vingt-cinq cercles. Le premier, le numéro 1 est ici (le montrer) et le dernier, le numéro 25 se trouve là (le montrer). Vous allez relier les 25 cercles en suivant l'ordre numérique et en essayant d'aller le plus vite possible. Vous y êtes ? Allez y, partez du numéro 1 (le montrer) et essayer d'aller le plus vite possible mais sans oublier de numéros ».
On déclenche le chronomètre dès que le sujet commence à relier le cercle 1 au cercle 2. Lorsque le sujet commet une erreur, on l'arrête aussitôt et on stoppe le chronomètre. On fait prendre conscience de son erreur au sujet et dès qu'il est reparti « sur la bonne piste », on relance le chronomètre.

Trail Making Test Part A – *SAMPLE*

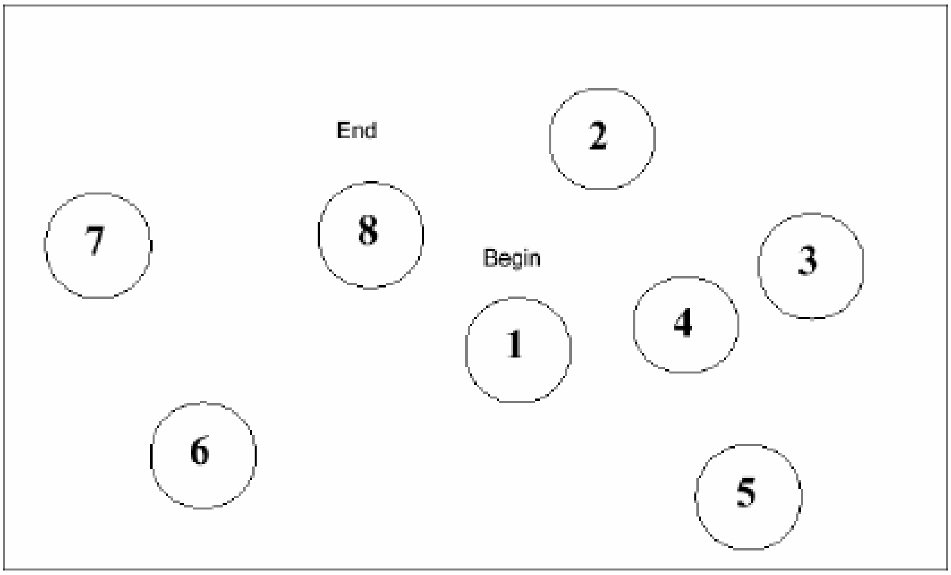

TMT PARTIE A

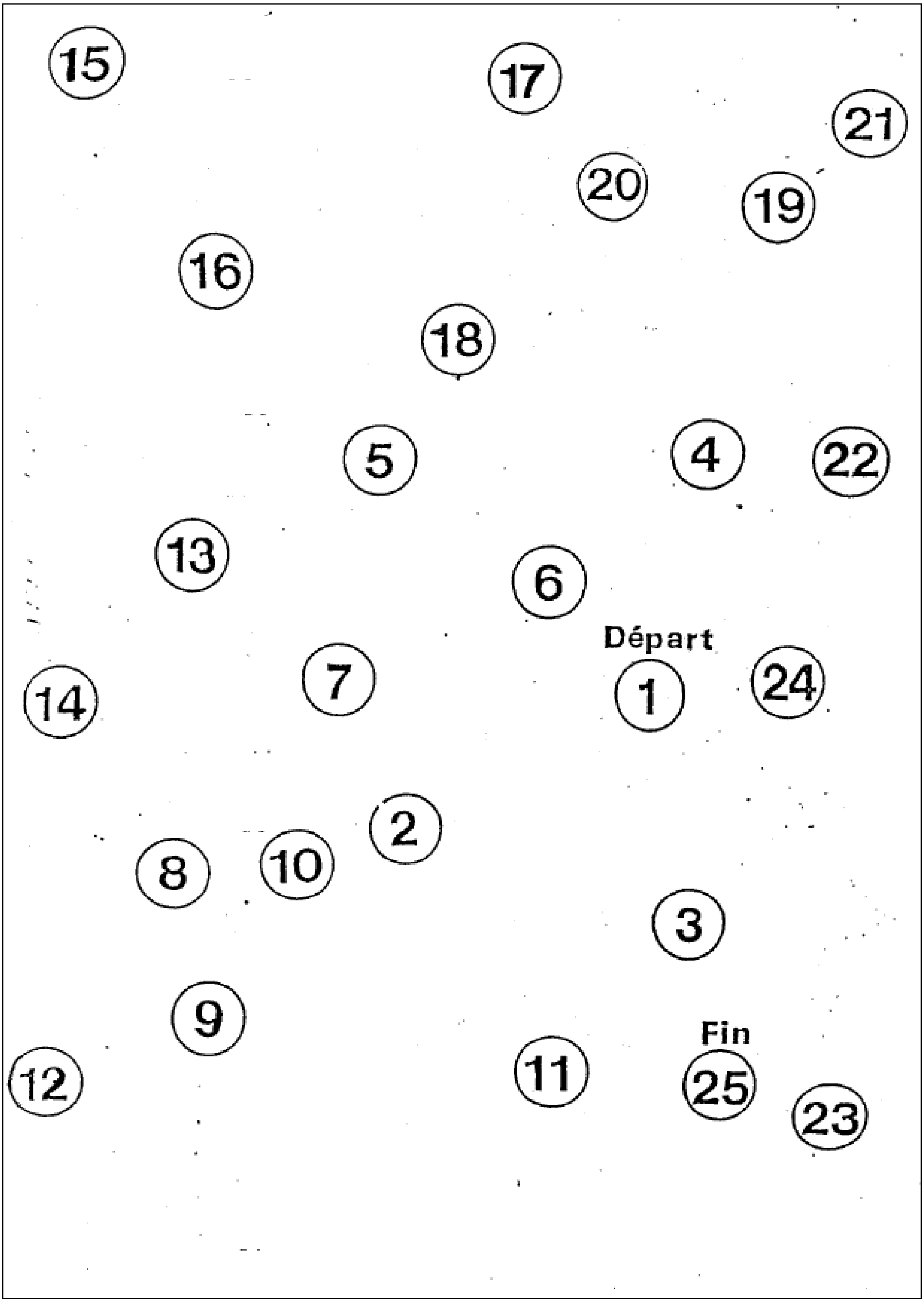
15
17
21
20
19
16
18
5
4
22
13
6
Départ
1
24
14
7
2
8
10
3
9
Fin
12
11
25
23

PARTIE B

Consigne :

Montrer la feuille-exemple (Sample) au sujet et lui dire : « Sur cette feuille se trouvent huit cercles numérotés de 1 à 4 et des cercles contenant des lettres de A à D. Je vous demande de relier ces huit cercles avec ce crayon, en alternant les chiffres et les lettres, c'est-à-dire en allant de 1 à A, de A à 2, puis de 2 à B…Vous avez compris ? ». Si le sujet semble ne pas comprendre, lui montrer ce qu'il doit faire avec le crayon, puis lui demander de refaire l'exemple avec une autre feuille. Lorsque le sujet a compris, lui présenter la feuille de test et lui dire : « Maintenant, nous allons faite la même chose avec cette feuille. Cette fois, il y a vingt-cinq cercles. Le premier numéro, le 1 est ici (le montrer), la première lettre, le A (la montrer) est ici, le dernier numéro, le 13, se trouve là (le montrer), et la dernière lettre le L se trouve là (la montrer). Vous allez relier les 25 cercles en alternant les chiffres et les lettres et en essayant d'aller le plus vite possible. Vous y êtes ? Allez y, partez du numéro 1 (le montrer) et essayer d'aller le plus vite possible mais sans oublier de numéros ou de lettres ».
On déclenche le chronomètre dès que le sujet commence à relier le cercle 1 au cercle A. Lorsque le sujet commet une erreur, on l'arrête aussitôt et on stoppe le chronomètre. On fait prendre conscience de son erreur au sujet et dès qu'il est reparti « sur la bonne piste », on relance le chronomètre.

Trail Making Test Part B – *SAMPLE*

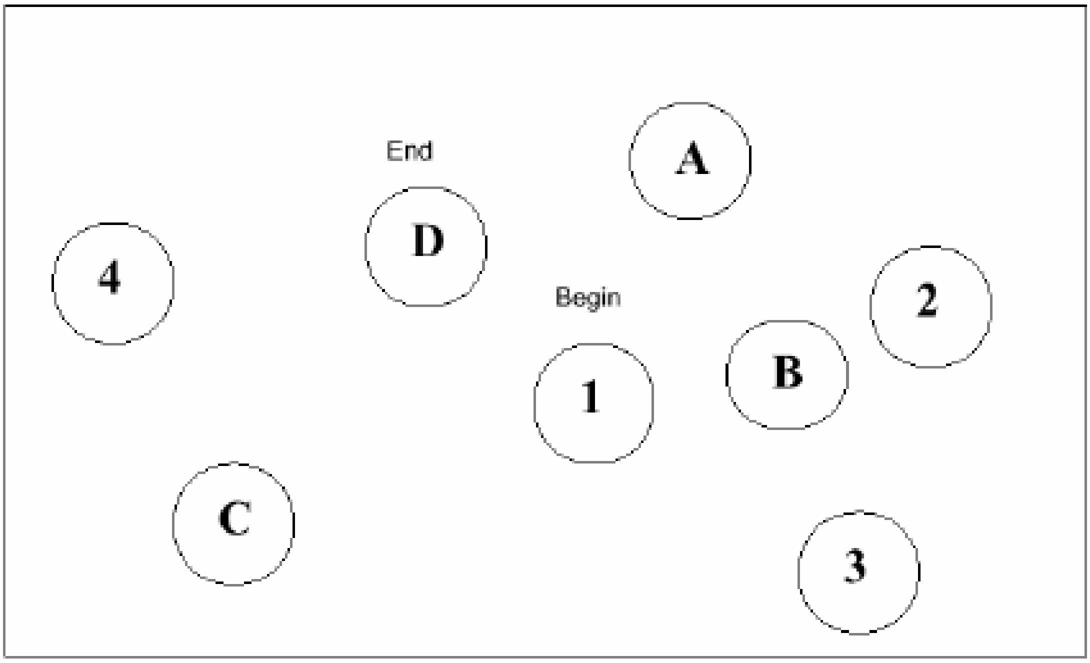

TMT PARTIE B

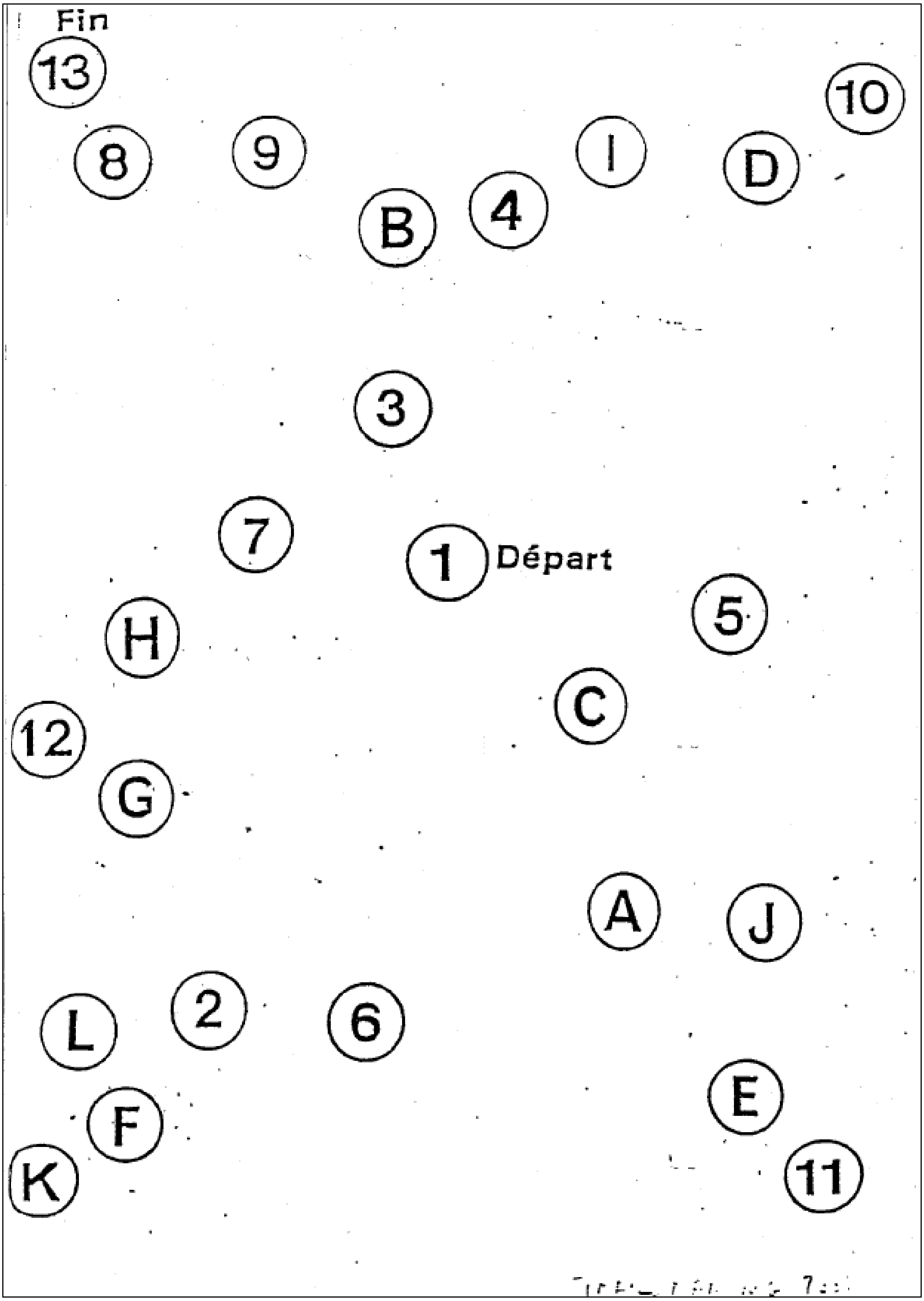

DIGIT SYMBOL SUBSTITUTION TEST

10. DIGIT SYMBOL

1	2	3	4	5	6	7	8	9	SCORE
—	⊥	⊐	L	⊔	O	∧	X	=	

SAMPLES

2	1	3	7	2	4	8	2	1	3	2	1	4	2	3	5	2	3	1	4	5	6	3	1	4

1	5	4	2	7	6	3	5	7	2	8	5	4	6	3	7	2	8	1	9	5	8	4	7	3

6	2	5	1	9	2	8	3	7	4	6	5	9	4	8	3	7	2	6	1	5	4	6	3	7

9	2	8	1	7	9	4	6	8	5	9	7	1	8	5	2	9	4	8	6	3	7	9	8	6

32.ANNEXE 13 – Carnet de suivi journalier

**CAHIER DE SUIVI JOURNALIER**<br>**SUR 3 MOIS POST OPERATOIRE**

Jour 0 à Jour 16:

JOUR 0	*DATE*	**CODE SUJET:**
	____ / ____ / _______	**I__I__I__I / I__I__I / I__I__I__I**

- **Evaluation pluriquotidienne de votre douleur par échelle numérique (avec l'aide des professionnels de santé),** entourez une seule note de 0 à 10 **:**

Matin : ____ H ____

Aucune douleur	0	1	2	3	4	5	6	7	8	9	10	Douleur maximale imaginable

Midi :____ H ____

Aucune douleur	0	1	2	3	4	5	6	7	8	9	10	Douleur maximale imaginable

Soir : ____ H ____

Aucune douleur	0	1	2	3	4	5	6	7	8	9	10	Douleur maximale imaginable

Nuit ____ H ____

Aucune douleur	0	1	2	3	4	5	6	7	8	9	10	Douleur maximale imaginable

Avez-vous pris des antidouleurs aujourd'hui ?	*OUI ☐*	*NON ☐*
Si oui veuillez compléter le tableau ci-dessous :		
Traitement	***Raison***	***Dose***

Jour 17 à 3 mois :

JOUR 17	*DATE*<br>____ / ____ / _______	**CODE SUJET:**<br>**I__I__I__I / I__I__I / I__I__I__I**

- **Evaluation de votre douleur moyenne sur la journée par échelle numérique** entourez une seule note de 0 à 10 **:**

Aucune douleur	0	1	2	3	4	5	6	7	8	9	10	Douleur maximale imaginable

Avez-vous pris des antidouleurs aujourd'hui ?	*OUI* ☐	*NON* ☐
Si oui veuillez compléter le tableau ci-dessous :		
Traitement	***Raison***	***Dose***
